# Total synthesis of palau'amine

**DOI:** 10.1038/ncomms9731

**Published:** 2015-11-04

**Authors:** Kosuke Namba, Kohei Takeuchi, Yukari Kaihara, Masataka Oda, Akira Nakayama, Atsushi Nakayama, Masahiro Yoshida, Keiji Tanino

**Affiliations:** 1 Department of Pharmaceutical Science, Tokushima University, 1-78 Shomachi, Tokushima 770-8505, Japan; 2 Graduate School of Chemical Sciences and Engineering, Hokkaido University, Sapporo 060-0810, Japan; 3 Department of Chemistry, Faculty of Science, Hokkaido University, Kita-ku, Sapporo 060-0810, Japan; 4 Graduate School of Medical and Dental Sciences, Niigata University, Chuo-ku, Niigata 951-8514, Japan; 5 Catalysis Research Center, Hokkaido University, Sapporo 001-0021, Japan

## Abstract

Palau'amine has received a great deal of attention in the past two decades as an attractive synthetic target by virtue of its intriguing molecular architecture and significant immunosuppressive activity. Here we report the total synthesis of palau'amine characterized by the construction of an ABDE tetracyclic ring core including a *trans*-bicylo[3.3.0]octane skeleton at a middle stage of total synthesis. The ABDE tetracyclic ring core is constructed by a cascade reaction of a cleavage of the N–N bond, including simultaneous formation of imine, the addition of amide anion to the resulting imine (D-ring formation) and the condensation of pyrrole with methyl ester (B-ring formation) in a single step. The synthetic palau'amine is confirmed to exhibit excellent immunosuppressive activity. The present synthetic route has the potential to help elucidate a pharmacophore as well as the mechanistic details of immunosuppressive activity.

## Abstract (web summary)

Thanks to its immunosuppressive activity and complex molecular architecture, the natural product palau'amine has attracted continuous attention over a number of years. Here, the authors report a new synthetic strategy which includes the formation of multiple rings in a single key step.

## Graphical abstract

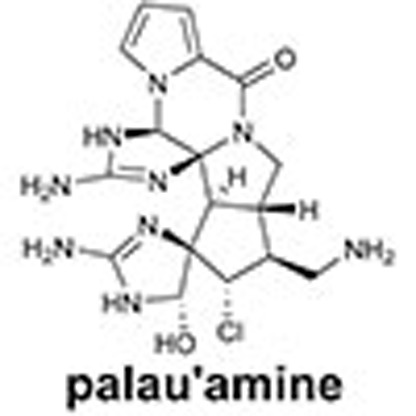

The pyrrole-imidazole alkaloids comprise a large family of natural products and have received a great deal of attention for their potent biological activities and tremendous structural diversity^1^,^2^. Palau'amine (**1**) was originally isolated from a sponge, *Stylotella agminata*, in 1993 by Scheuer and colleagues^3^,^4^ as a novel class of pyrrole-imidazole alkaloids and the proposed structure was revised in 2007 (refs 5, 6, 7, 8, 9). Since the initial disclosure of a proposed structure and the later structural revision, palau'amine (**1**) has been an attractive synthetic target because of its intriguing molecular architecture and significant biological properties, which include antifungal, antitumour and immunosuppressive activities. The immunosuppressive activity has been of particular interest and two studies on its mode of action have been reported^10^,^11^. Thus, development of **1** as molecular probes is required for further elucidation of the potential of palau'amine as an immunosuppressive agent. In addition, investigations into the structure–activity relationship will also be needed for the development of novel lead compound of immunosuppressive agent. However, palau'amine has been a well-known natural product and proven to be the most challenging synthetic target. The noteworthy structural features of palau'amine include its two guanidine moieties, fused polycyclic system with a spiro cycle, fully substituted complex cyclopentane ring, eight contiguous stereogenic centres including a nitrogen-substituted quaternary carbon centre and *trans*-bicyclo[3.3.0]octane skeleton (D/E ring junction). Not surprisingly, many attempts to synthesize palau'amine^9^,^12^,^13^,^14^,^15^,^16^,^17^,^18^,^19^,^20^,^21^,^22^,^23^,^24^,^25^,^26^,^27^,^28^,^29^,^30^ and related compounds^31^,^32^,^33^,^34^,^35^,^36^,^37^ have been reported, and numerous reviews of these different approaches have also been published^38^,^39^,^40^,^41^,^42^,^43^. To date, however, there has been only one report of a total synthesis, by Baran and colleagues^44^ in 2010, which was followed by the development of an asymmetric version in 2011 (ref. 45). The most difficult challenge in the total synthesis of **1** would be the construction of a *trans*-bicyclo[3.3.0]octane system that corresponds to a D/E ring junction and Baran and colleagues^44^ has addressed this by adopting the transannular reaction of nine-membered lactam **2**, named macro-palau'amine, leading to **1** at the final step (Fig. 1). Despite their short and elegant synthetic route to **1**, however, the total synthesis of palau'amine is still a challenging undertaking. In particular, as the basic structure of **1** remains to be established, there is need of an efficient method for constructing an ABDE tetracyclic ring system that includes a *trans*-bicyclo[3.3.0]octane skeleton and, although the development of such a system will be extremely difficult, it is also absolutely critical for not only the field of synthetic organic chemistry but also the elucidation of pharmacophores and the development of palau'amine probes.

Herein we report the successful establishment of an alternative synthetic route to **1** based on the efficient construction of an ABDE tetracyclic ring core at the middle stage of total synthesis, in which many analogues of **1** possessing various ring core systems are first obtained.

## Results

### Synthetic plan

**Figure 1:**
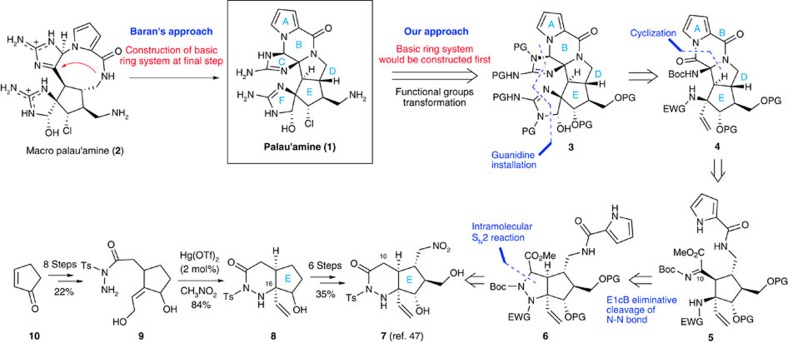
Structure of palau'amine and its synthetic approach. Palau'amine **1** should be obtained by functional group transformations from the hexacyclic ring core **3**, which would be constructed from the previously reported E-ring core **7**. Hg(OTf)_2_, mercury(II) trifluomethanesulfonate; PG, protecting group; EWG, electron-withdrawing group.

Our synthetic plan of **1** is outlined in Fig. 1. In this synthesis, palau'amine **1** would be obtained by the transformation of functional groups after the construction of a hexacyclic ring core **3**. The two cyclic guanidines corresponding to the C and F rings of **3** would be built on amino and carbonyl groups of ABDE tetracyclic ring core **4**. The B and D rings of **4** would be formed by a sequential cyclization reaction of amide and pyrrole nitrogen with the imine and methyl esters of **5**, respectively. As the acylimine moiety of **5** is highly electron deficient, the nucleophilic addition of amide anion to the C10 carbon centre would occur to form a D ring over the steric strain of the *trans*-bicyclo[3.3.0]octane skeleton (D/E ring junction). The iminoester moiety of **5** would be generated from hydrazide **6** by a N–N bond cleavage and a formation of imine at the C10 position. Overman *et al*.^12^ have also employed a hydrazine fragmentation in their efforts towards the palau'amine core and the reductive cleavage of the N–N bond occurred spontaneously due to the high strain of the pyrazolidine ring. On the other hand, we planned to adopt an E1cB (E-elimination, 1cB-first order with respect to conjugate base) eliminative cleavage of the N–N bond of **6**, to obtain iminoester **5** directly, although, unlike in the case of reductive cleavage, there have been very few examples of E1cB eliminative cleavage of N–N bonds^35^,^46^. The direct formation of **5** under basic conditions was expected to induce further cascade cyclization reactions leading to **4**, that is, the ABDE tetracyclic ring core **4** might be obtained from **6** in a single step. The pyrazolidine ring of **6** would be obtained from **7** by the introduction of an electron-withdrawing group and ring contraction via an intramolecular S_N_2 reaction. In 2009, we reported an efficient synthesis of the cyclopentane core **7** (ref. 47). The synthesis of **7** began with commercially available cyclopentenone **10**, which was converted into acyltosylhydrazide **9** in 22% overall yield after eight steps. The mercury(II) trifluomethanesulfonate (Hg(OTf)_2_)-catalysed cyclization reaction^48^ of **9** proceeded smoothly to yield a tetra-substituted carbon centre corresponding to the C16 position of **1** and piperazine **8** was obtained in 84% yield. Compound **8** was subsequently converted into **7** as a single diastereomer in 35% yield by a sequence consisting of oxidation to enone, Morita–Baylis–Hillman reaction, 1,4-addition of nitromethane and reduction of ketone (see Supplementary Fig. 1). First, therefore, we attempted to synthesize the E1cB eliminative cleavage precursor **6** from the compound **7**.

### Synthesis of the cascade cyclization precursor **6**

**Figure 2:**
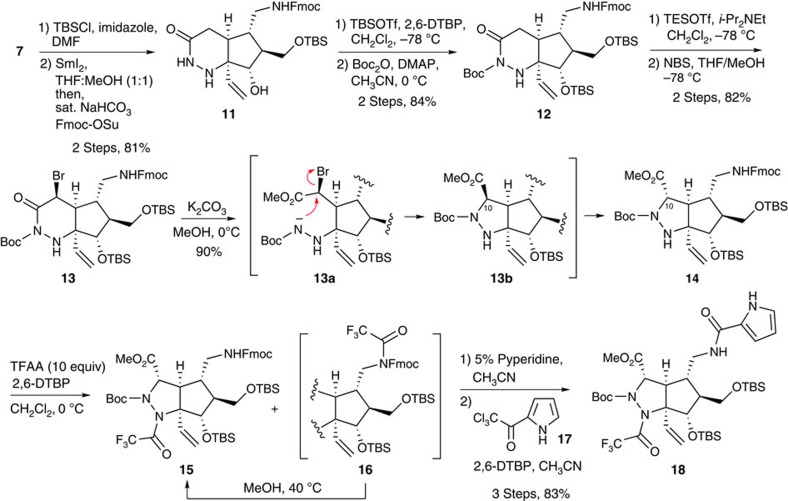
Synthesis of precursor 18 for the cascade reaction. The precursor **18** was synthesized from **7** via changes of the protecting group, bromination at the C10 position, metholysis followed by intramolecular S_N_2 reaction and introductions of trifluoroacetyl group and pyrrole amide. TBSCl, *tert*-butylchlorodimethylsilane; DMF, dimethylformamide; Fmoc-OSu, *N*-(9-fluorenylmethoxycarbonyloxy)succinimide; TBSOTf, *tert*-butyldimethylsilyl trifluoromethanesulfonate; 2,6-DTBP, 2,6-Di-*tert*-butylpyridine; Boc_2_O, di-*tert*-butyl dicarbonate; DMAP, 4-dimethylaminopyridine; TESOTf, triethylsilyl trifluoromethanesulfonate; NBS, *N*-bromosuccinimide; TFAA, trifluoroacetic anhydride.

After introduction of the *tert*-butylchlorodimethylsilyl (TBS) group into the primary alcohol of **7** to increase hydrophobicity, the tosyl group was reductively removed and the nitro group was reduced to the amino group by treatment with SmI_2_. The resulting primary amine was protected by the 9-fluorenylmethyloxycarbonyl (Fmoc) group to afford **11** in 81% yield from **7**. Further protections of secondary alcohol and acylhydrazide proceeded smoothly to give **12** by treatment with *tert*-butyldimethylsilyl trifluoromethanesulfonate as a silylation reagent followed by di-*tert*-butyl dicarbonate in the presence of a catalytic amount of 4-dimethylaminopyridine. As we expected, Boc protection occurred on the nitrogen possessing the acyl group due to the acidity of NH protons and steric hindrance^49^. Treatment of **12** with triethylsilyl trifluoromethanesulfonate and 2,6-di-*tert*-butyl pyridine at −78 °C readily afforded silyl ketene aminal, and the crude product was treated with *N*-bromosuccinimide in a mixed solvent of tetrahydrofurane (THF) and methanol to give bromide **13** in an 82% two-step yield along with 7–14% of starting material **12**. The stereochemical outcome of **13** was determined by a nuclear Overhauser effect spectroscopy experiment (Supplementary Fig. 2), which indicated the β-configuration of bromide. It is likely to be that the attack of bromide on the concave face was derived from the steric hindrance of the vinyl group. Subsequent methanolysis of **13** afforded an amide anion **13a** that immediately induced an intramolecular S_N_2 reaction, leading to **13b**. At this stage, **13b** was considered to readily epimerize to **14**, to avoid the steric repulsion of a tighter concave face. The stereochemistry of **14** was determined by a nuclear Overhauser effect spectroscopy experiment (Supplementary Fig. 3). Next, we attempted to introduce a strong electron-withdrawing group to nitrogen on a tetra-substituted carbon centre. After various examinations, we found that only a trifluoroacetyl group could be introduced to afford **14** by treatment with an excess amount of trifluoroacetic anhydride. Although overacetylated product **16** was also obtained, the extra trifluoroacetyl group on the carbamate was automatically removed in methanol at 40 °C in a quantitative yield. Finally, removal of the Fmoc group and condensation of the resulting primary amine with pyrrole trichloromethyl ketone **17** afforded **18** as a precursor of the key cascade reaction in an 83% three-step yield (Fig. 2).

### Construction of the ABDE tetracyclic ring core

**Figure 3:**
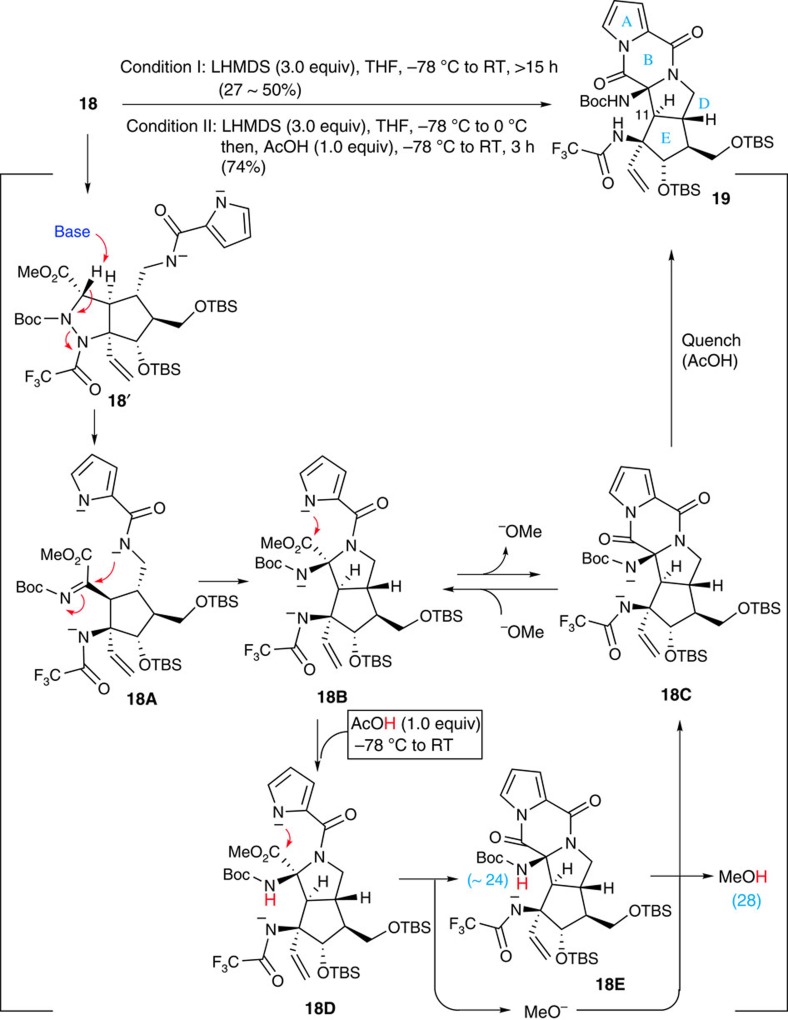
Construction of ABDE ring system and comparison of the relative basicity of nitrogen anions. The ABDE tetracyclic ring core was constructed by a cascade reaction of a cleavage of the N–N bond, including simultaneous formation of imine, the addition of amide anion to the resulting imine (D-ring formation) and the condensation of pyrrole with methyl ester (B-ring formation) in a single step. LHMDS, lithium hexamethyldisilazide.

Having prepared the cleavage precursor **18**, we next attempted the single-step construction of an ABDE tetracyclic ring system. To induce an E1cB eliminative cleavage of the N–N bond, **18** was treated with 3.0 equiv of lithium hexamethyldisilazide (LHMDS) as a strong base at −78 °C and warmed to room temperature (condition I). The reaction afforded the expected tetracyclic compound **19** that corresponds to the ABDE ring of palau'amine, including the *trans*-bicyclo[3.3.0]octane skeleton, in modest yield (Fig. 3). The transfused D/E ring junction and the stereochemistry of **19** were confirmed by a nuclear Overhauser effect experiment and by comparison of the coupling constant with the natural product (see Supplementary Figs 4 and 5). It was noteworthy that the coupling constant at the C11 proton indicated the characteristic value (*J*=14.5 Hz) of a *trans*-bicyclo[3.3.0]octane skeleton. The reaction pathway leading to the ABDE ring system **19** is explained below. Treatment of **18** with 3.0 equiv of a strong base would abstract two NH protons and hydrogen at the C10 position, thereby inducing a β-elimination of nitrogen possessing an electron-withdrawing group of **18'**, as shown in Fig. 3. This would lead simultaneously to both the cleavage of the N–N bond and the imine formation at the C10 position to give **18A**. The nucleophilic addition of amide anion to the electron-deficient C10 carbon centre would occur immediately to give **18B** possessing a *trans*-bicyclo[3.3.0]octane skeleton (D/E ring junction). Furthermore, the cascade reaction would not stop at **18B** due to the remaining pyrrole anion and subsequent condensation of pyrrole with methyl ester formed a B ring to give **19** (Fig. 3). The results encouraged us to scale up this reaction from a few milligrams to 100 mg with the goal of completing the total synthesis of palau'amine. However, the reaction was found to suffer from poor reproducibility, often leading to poor to low yields even after a prolonged reaction time. After careful consideration of the reaction mechanism, we established an improved method involving the partial protonation of the anionic intermediates with acetic acid (condition II). On treatment with 3.0 equiv of LHMDS, substrate **18** undergoes stepwise lithiation of the two NH protons at −78 °C and then the C10-proton at around 0 °C. Once the C10 proton is abstracted, the formation of **18A** and the subsequent cyclization of amide anion leading to **18B** would proceed rapidly and completely. Indeed, only **18B** was detected as its protonated form by thin layer chromatography (TLC) (see Supplementary Fig. 6), indicating the fast conversion of **18A** to **18B** and the slow formation of **18C** from **18B**. The smaller p*K*a value (in dimethyl sulfoxide) of the pyrrole moiety (should be <23 of pyrrole) compared with that of methanol (28) predicted that the equilibrium between **18B** and **18C** favoured the former, which led us to remove the methoxide ion without quenching the pyrrole anion. Thus, after checking the conversion of **18** into **18B** by TLC, the mixture was cooled to −78 °C again and an exact 1.0 equiv of acetic acid was slowly added. On warming to room temperature, the desired product **19** was obtained in up to 74% yield with good reproducibility in acceptable scales for total synthesis (Fig. 3).

The mechanism underlying the cascade reaction under ‘condition II' is explained as follows. The intermediate **18B** possesses three nitrogen anions of Boc-carbamate (∼24), pyrrole (<23) and trifluoroacetoamide (∼17), according to the order of basicity by comparison of the p*K*a values of protonated NH functional groups. Thus, an exact 1.0 equiv of acetic acid would protonate only the anion of Boc-carbamate and trianion **18B** would be converted into dianion **18D**. Although the mixture was warming to room temperature, the remaining pyrrole anion induced condensation with methyl ester, simultaneously generating methoxide as in the case with trianion. However, as the condensation product **18E** possesses an active NH proton, unlike in the case with trianion, methoxide does not attack the pyrrole amide but extracts the active Boc-NH proton to give the same dianion **18C** with a quenching methoxide anion. Among various acids, including phenol and imide derivatives, acetic acid was found to be the best protonating reagent. In addition, precise amounts of LHMDS and acetic acid are very important for this p*K*a game reaction. As stated above, we succeeded in establishing an efficient method for constructing the basic core structure of palau'amine.

### Theoretical calculations on the coordination effect of lithium

**Figure 4:**
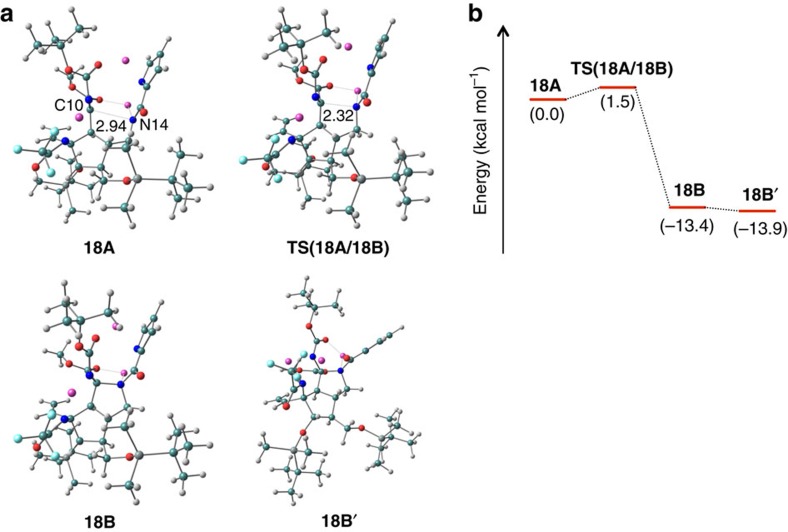
Optimized structures and energy diagram of 18. (**a**) Optimized structures of **18A**, **18B**, **TS(18A/18B)** and **18B′**. **TS(18A/18B)** is a transition-state structure connecting **18A** and **18B**. **18B′** represents a structure after the translocation of a lithium atom. The atoms were colour coded as follows: grey, H; pink, Li; green, C; blue, N; red, O; light blue, F; dark grey, Si. (**b**) Potential energy profile for the cyclization reaction (**18A**→**18B**). Calculations were performed by the density functional theory (DFT) using the modern functionals of M06-2X with the 6-31G* basis set. The solvent effects are taken into account by the self-consistent reaction field (THF). The potential energies (in kcal mol^−1^) relative to **18A** are shown in parentheses. All calculations were performed by the Gaussian 09 package^58^.

The remaining question in this cascade reaction is how the amide anion of **18A** got close to the C10 carbon centre, to overcome the steric strain of the *trans*-bicyclo[3.3.0]octane skeleton, although the Boc-imine moiety of **18A** was highly electron deficient. Thus, we focused on the coordination effect of lithium ion. If the lithium countercation of amide anion formed a coordination bond with the carbonyl group of methyl ester, the amide nitrogen (N14) and the C10 carbon centre would be located at transannular positions that are close to each other due to the strain of the eight-membered ring. To investigate this coordination effect, theoretical calculations were carried out by the density functional theory method. The optimized structure of **18A** including lithium ions is shown in Fig. 4a (the effects of coordination of solvent THF molecules to lithium ions are discussed in the Supplementary Data; see the Supplementary Discussion and Supplementary Figs 7 and 8). Interestingly, the calculation actually indicated not only the expected coordination of lithium amide to the carbonyl group of methyl ester but also the unpredictable coordination of lithium salt of pyrrole anion to the carbonyl oxygen of the Boc group. Therefore, the two *trans*-oriented side chains were quite close to each other by the chelation to two lithium ions and the distance between N14 and C10 at the transannular positions was calculated to be only 2.94 Å. Owing to the short distance between the two reaction points, the energy barrier of the cyclization reaction (**18A**→**18B**) was estimated to be only 1.5 kcal mol^−1^, allowing the reaction to proceed smoothly (see Fig. 4b for the potential energy profile of the cyclization reaction). Thus, the nucleophilic addition of the amide anion (N14) occurred before the rotation of the single bond of C10–C11, which was also restricted by the coordination effect of lithium ion, to afford **19** as a single diastereomer possessing the β-configuration of the NHBoc group at the C10 position. From the calculated result, it was concluded that the chelation effect of lithium ion played a significant role in the formation of the *trans*-bicyclo[3.3.0]octane skeleton. In fact, this cascade reaction in the presence of 3.6 equiv of hexamethylphosphoric triamide as a lithium ion scavenger afforded a complex mixture and the desired cyclized (D/E ring) products and related intermediates were not detected. Furthermore, the use of other bases, such as sodium hexamethyldisilazide (NaHMDS) and potassium hexamethyldisilazide (KHMDS), also did not give any desired cyclization products. In addition, the yield of tetracyclic **19** was dramatically decreased to 10∼20%, when the initial treatment of 3.0 equiv of LHMDS was directly conducted at 0 °C. This result clearly suggested that the coordination of lithium ions to the two carbonyl groups must be formed first at −78 °C before the extraction of the C10 proton occurs at 0 °C.

### Total synthesis of palau'amine

**Figure 5:**
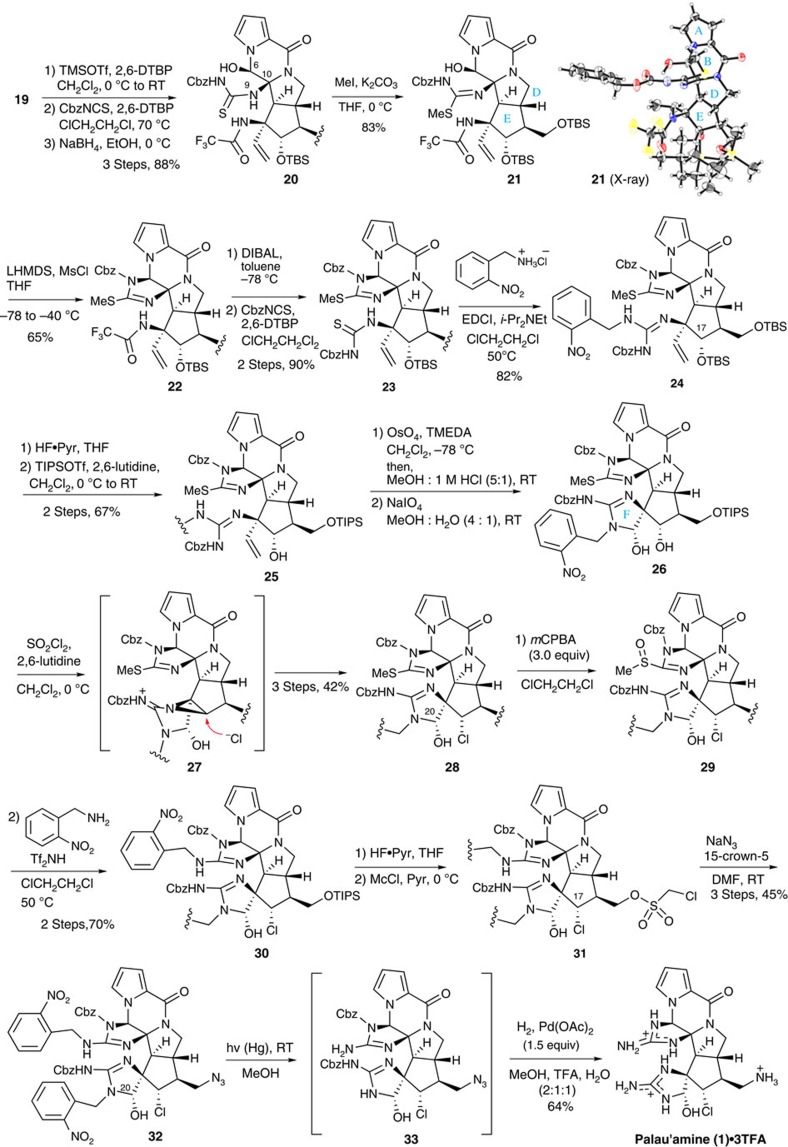
Total synthesis of palau'amine 1. The ABDE tetracyclic ring core **19** was successfully converted into **1** by the sequential operations of a C-ring formation, a F-ring formation, a substitution of chloride at C17 for secondary alcohol, a guanidine formation on the C-ring, substitution of an amine equivalent for the primaly alcohol and deprotections. CbzNCS, *N*-benzyloxycarbonyl isothiocyanate; MsCl, methanesulfonyl chloride; DIBAL, diisobutylaluminium hydride; EDCI, 1-ethyl-3-(3-dimethylaminopropyl)carbodiimide; TIPSOTf, triisopropylsilyl trifluoromethanesulfonate; TMEDA, tetramethylethylenediamine; *m*CPBA, m-chloroperoxybenzoic acid; McCl, chloromethylsulfonyl chloride.

Having established an efficient method for constructing an ABDE ring system that overcame the most difficult barrier to the total synthesis of palau'amine, we attempted a total synthesis from **19** (Fig. 5). We first tried to construct a C-ring by using an amino group (N9) on C10 and a carbonyl group at C6 as a foothold. The amino group (N9) of **19** was converted into thiourea by removal of the Boc group according to Ohfune's method^50^ followed by treatment with *N*-benzyloxycarbonyl isothiocyanate (CbzNCS) and the pyrrole amide at C6 was selectively reduced to hemiaminal **20** as an isolable compound in 88% overall yield after three steps from **19**. The removal of the Boc group did not proceed after the reduction of the pyrrole amide, indicating that the planar configuration of the *sp*^2^ carbon centre reduced the steric hindrance around the N9 amino group. Furthermore, the use of carbodiimide derivatives for the direct formation of guanidine did not proceed and only CbzNCS as a small and reactive reagent reacted with the sterically hindered resulting N9 amine. As the thiourea moiety of **20** could not be directly converted into guanidine due to the predominant cyclization from hemiaminal oxygen to activated thiourea, the thiourea moiety was converted into isothiourea **21**. At this stage, the structure of the synthetic intermediate including the *trans*-bicyclo[3.3.0]octane skeleton was unambiguously confirmed by an X-ray diffraction study (Supplementary Method and Supplementary Data 1). Treatment of **21** with LHMDS and methanesulfonyl chloride at −78 to −40 °C induced a sequential reaction of mesylation, the elimination of mesylate and the addition of nitrogen to the isothiourea moiety to give pentacyclic **22** in 65% yield, in a manner similar to that of the synthesis of (+)-dibromophakellstatin reported by Nagasawa and colleagues^51^. Having constructed a C-ring, we next tried to form an F-ring. Only the reductive condition using diisobutylaluminium hydride was successful for the removal of the trifluoroacetyl group and subsequent treatment of the resulting amine with CbzNCS afforded thiourea **23** in good yield. The thiourea moiety was directly converted into guanidine **24** by the condensation using 1-ethyl-3-(3-dimethylaminopropyl)carbodiimide^52^ with *o*-nitrobenzylamine in 82% yield. However, the oxidative cleavage of the vinyl group without the oxidation of pyrrole was difficult, probably due to the steric hindrance of the TBS group at the C17 position. Thus, two TBS groups were removed by hydrogen fluoride (HF)·pyridine and only primary alcohol was protected again by the triisopropylsilyl group, to give **25** in a 67% two-step yield. In the case of the free secondary hydroxyl group, dihydroxylation using OsO_4_ and tetramethylethylenediamine in dichloromethane^53^ proceeded smoothly at −78 °C to give osmate and subsequent hydrolysis in the mixed solution of methanol and 1 M HCl afforded the desired diol. The oxidative cleavage of diol was successful only in the mixed solution of methanol and water (4:1) to give an unstable F-ring product **26**, which gradually decomposed even under the neutral condition, due probably to the retro-aldol reaction at the C17 position. Product **26** was then directly used for the next reaction without purification. With the construction of an ABCDEF ring system of palau'amine, the conversions of functional groups into **1** were finally attempted. The substitution of the secondary hydroxyl group to chloride with stereoretention proceeded by the use of the neighbouring-group effect of guanidine on the F-ring. The activation of secondary alcohol by using sulfuryl chloride induced the participation of guanidine depicted as **27** in analogy with ‘massadine aziridine'^54^ and the subsequent nucleophilic attack of chloride afforded **28** with an α-configuration of chloride, along with a small amount of epimer at the C20 position as an unnatural configuration. On the other hand, the hydroxyl group of hemiaminal also reacted with excess sulfuryl chloride to give dichloride when more than 1.0 equiv of sulfuryl chloride was used, and the chloride at the C20 position was readily hydrolysed to convert back to **28**. Next, we had to develop a new method for converting the methylthio group into an amino group, leading to guanidine under acidic conditions, because we found that hexacyclic intermediates were readily decomposed under the basic conditions. After various examinations, we achieved the desired conversion by the reaction of a trifluoromethanesulfonyl imide salt of *o*-nitrobenzylamine with sulfoxide **29** to give **30** in a 70% two-step yield. The overoxidation into sulfone and the use of a salt other than trifluoromethanesulfonic imide afforded only a complex mixture. In the final transformation of the functional group, the primary alcohol was converted into chloromethanesulfonate **31** (ref. 55) after removal of the triisopropylsilyl group. The azidation reaction of **31** proceeded at room temperature to afford **32** as a protected palau'amine. When methanesulfonate was adopted as a leaving group, the azidation reaction required a high temperature (50 °C) that also induced azidation at the C17 position via the neighbouring-group effect. Fortunately, the minor epimer of the unnatural configuration at the C20 position disappeared at this stage. Finally, Hg-lump irradiation followed by direct hydrogenation as a deprotection of **32** afforded **1** in 64% yield as a 3TFA salt. Synthetic **1** showed spectral data (^1^H and ^13^C nuclear magnetic resonance (NMR), high-resolution mass spectra) completely identical to those of natural palau'amine **1** (refs 3, 7). Throughout this total synthesis, **1** was obtained in 0.039% overall yield after 45 steps (78% average yield at each step) from commercially available cyclopentenenone **10**. As a result of the 45-step synthesis, many synthetic intermediates as derivatives of **1** possessing various ring core systems were actually obtained for the first time. In future studies, we plan to perform activity evaluations for each deprotected synthetic intermediate, to further elucidate the pharmacophore.

### Biological test of synthetic palau'amine

**Figure 6:**
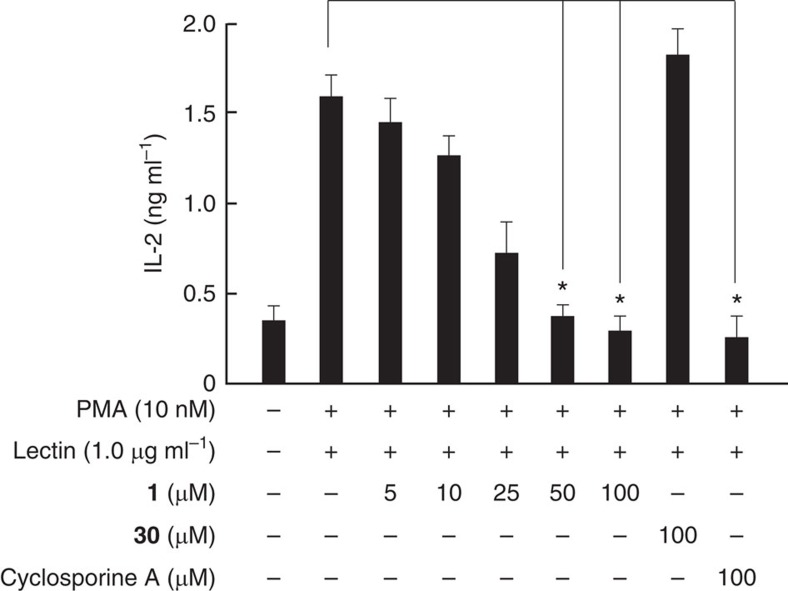
Immunosuppressive activity of synthetic palau'amine at various concentrations. Balb/c splenic lymphocytes were treated with various concentrations of an aqueous solution of synthetic **1**, synthetic **30** (100 μM) and cyclosporine A (100 μM) at 37 °C for 1 h, and then the cells were incubated with phorbol 12-myristate 13-acetate (PMA, 10 nM) and lectin (1.0 μg ml^−1^). After incubation for 5 h, the IL-2 in culture supernatant was measured by using an enzyme-linked immunosorbent assay kit (error bars, s.e.m.; *P<0.01).

Finally, the immunosuppressive activity of synthetic palau'amine was examined (Fig. 6). Lymphocytes derived from a mouse spleen were treated with various concentrations of an aqueous solution of synthetic **1** for 1 h and then the cells were incubated with phorbol 12-myristate 13-acetate and lectin^56^. After incubation for 5 h, the interleukin-2 (IL-2) in the culture supernatant was measured by using an enzyme-linked immunosorbent assay kit. Thereupon, the synthetic palau'amine·3TFA salt was confirmed to exhibit strong immunosuppressive activity, as shown in Fig. 6. The IC50 value was determined to be 45.3 μM, which is a practical level of activity, as it was for cyclosporine A. Not surprisingly, the synthetic intermediate **30** as a negative control did not exhibit immunosuppressive activities, due to the large hydrophobic protecting groups of all polar functional groups, and it slightly increased the production of IL-2. Therefore, we confirmed that not only the spectral data but also the biological properties of synthetic **1** were identical to those of natural palau'amine. Recently, Tepe and coworkers^10^,^11^ reported that palau'amine **1** and its related natural products inhibit I*κ*Bα degradation. On the other hand, we revealed that the synthetic palau'amine·3TFA salt inhibited the release of IL-2 from lymphocytes in the same manner as cyclosporine A, which is known to inhibit the I*κ*Bα degradation and nuclear factor-κB^57^. Thus, our synthetic palau'amine·3TFA salt was also expected to exhibit a similar inhibition of I*κ*Bα degradation.

## Discussion

In summary, we have achieved the total synthesis of palau'amine **1** via the single-step construction of an ABDE tetracyclic ring system. In the single-step construction, the chelation effect of lithium salt forming an eight-membered ring was crucial for the construction of a *trans*-bicyclo[3.3.0]octane skeleton (D/E ring junction) by bringing two reaction points close to each other at the transannular position. With the establishment of an efficient method for constructing a tetracyclic basic structure of palau'amine, various synthetic analogues for structure–activity relationship study and chemical probes of palau'amine would be accessible. For example, protected palau'amine **28** might be able to introduce labeling groups at desired spots on a C-ring, F-ring or a primary amine of an E-ring. Therefore, the chemistry described here offers not only a solution to a formidable synthetic challenge but also an alternative synthetic route to elucidate a pharmacophore and the mechanistic details of bioactivities of palau'amine. As our synthetic route is actually too long to develop **1** as a practical immunosuppressive agent at the current stage, further improvement of the key cascade cyclization reaction and the development of a shorter route to the cyclization precursors as a second-generation synthesis are also currently underway in our laboratory.

## Methods

### General

All the reaction were carried out in a round-bottomed flask with an appropriate number of necks and side arms connected to a three-way stopcock and/or a rubber septum cap under an argon atmosphere. All vessels were first evacuated by rotary pump and then flushed with argon before use. Solution and solvent were introduced by hypodermic syringe through a rubber septum. During the reaction, the vessel was kept under a positive pressure of argon. Dry THF was freshly prepared by distillation from benzophenone ketyl before use. Anhydrous CH_2_Cl_2_, dimethylformamide, ethanol, MeCN, methanol, pyridine and toluene were purchased from Kanto Chemical Co. Inc.

Infrared spectra were recorded on JASCO FT/IR-4100 spectrophotometer using 5 mm KBr plate. Wavelengths of maximum absorbance are quoted in cm^–1^. ^1^H-NMR spectra were recorded on a JEOL ECA-400 (400 MHz), JEOL ECA-500 (500 MHz) and Bruker AV-500 (500 MHz) in CDCl_3_, *d–*MeCN and D_2_O, respectively. Chemical shifts are reported in p.p.m. and signals are expressed as singlet (s), doublet (d), triplet (t), quartet (q), multiplet (m) and broad (br). ^13^C-NMR spectra were recorded on a JEOL ECA-400 (100 MHz), Bruker AV-400N (100 MHz) and Bruker AV-500 (125 MHz) in CDCl_3_, C_6_D_6_, CD_3_CN and D_2_O. Chemical shifts are reported in p.p.m. High-resolution mass spectra were recorded on a Thermo Scientific Exactive, Instrumental Analysis Division, Equipment Manager Center Creative Research Institution, Hokkaido University and a Waters SYNAPT-G2 Si HDMS, Tokushima Bunri University. HPLC was recorded on a HITACHI D-2,500 Chromato-Integrater. Analytical TLC was performed using 0.25 mm E Merck Silica gel (60F-254) plates. Reaction components were visualized with phosphomolybdic acid or ninhydrin or *p*-anisaldehyde in 10% sulfuric acid in ethanol. Kanto Chem. Co. Silica Gel 60N (particle size 0.040–0.050 mm) was used for column chromatography. Mouse IL-2 ELISA was purchased from Biolegend.

### Experimental data

For ^1^H and ^13^C NMR spectra of compounds, see Supplementary Figs 9–48. For the comparisons of ^1^H and ^13^C spectra of the natural and synthetic palau'amine, see Supplementary Figs 49 and 50, and Supplementary Tables 1 and 2. For the HPLC of synthetic palau'amine, see Supplementary Fig. 51. For the Cartesian Coordinates from density functional theory calculations (in Å) of **18**, see Supplementary Tables 3–7. For the experimental procedures and spectroscopic and physical data of compounds and the crystallographic data of compound **21**, see Supplementary Methods.

## Additional information

**Accession code:** The X-ray crystallographic coordinates for structures 21 reported in this study have been deposited at the Cambridge Crystallographic Data Centre (CCDC), under deposition number 1417980. These data can be obtained free of charge from The Cambridge Crystallographic Data Centre via www.ccdc.cam.ac.uk/data_request/cif.

**How to cite this article:** Namba, K. *et al*. Total synthesis of palau'amine. *Nat. Commun.* 6:8731 doi: 10.1038/ncomms9731 (2015).

## Supplementary Material

Supplementary Information Supplementary Figures 1-51, Supplementary Tables 1-8, Supplementary Discussion, Supplementary Methods and Supplementary References

Supplementary Data 1 Crystallographic information file
