# Supplementary material for: Total synthesis of palau'amine
Source: Nat Commun. 2015 Nov 4;6:8731. doi: 10.1038/ncomms9731 (PMC4667646; doi:10.1038/ncomms9731)
Supplement: Supplementary Information — Supplementary Figures 1-51, Supplementary Tables 1-8, Supplementary Discussion, Supplementary Methods and Supplementary References [file ncomms9731-s1.pdf]

Supplementary Figures

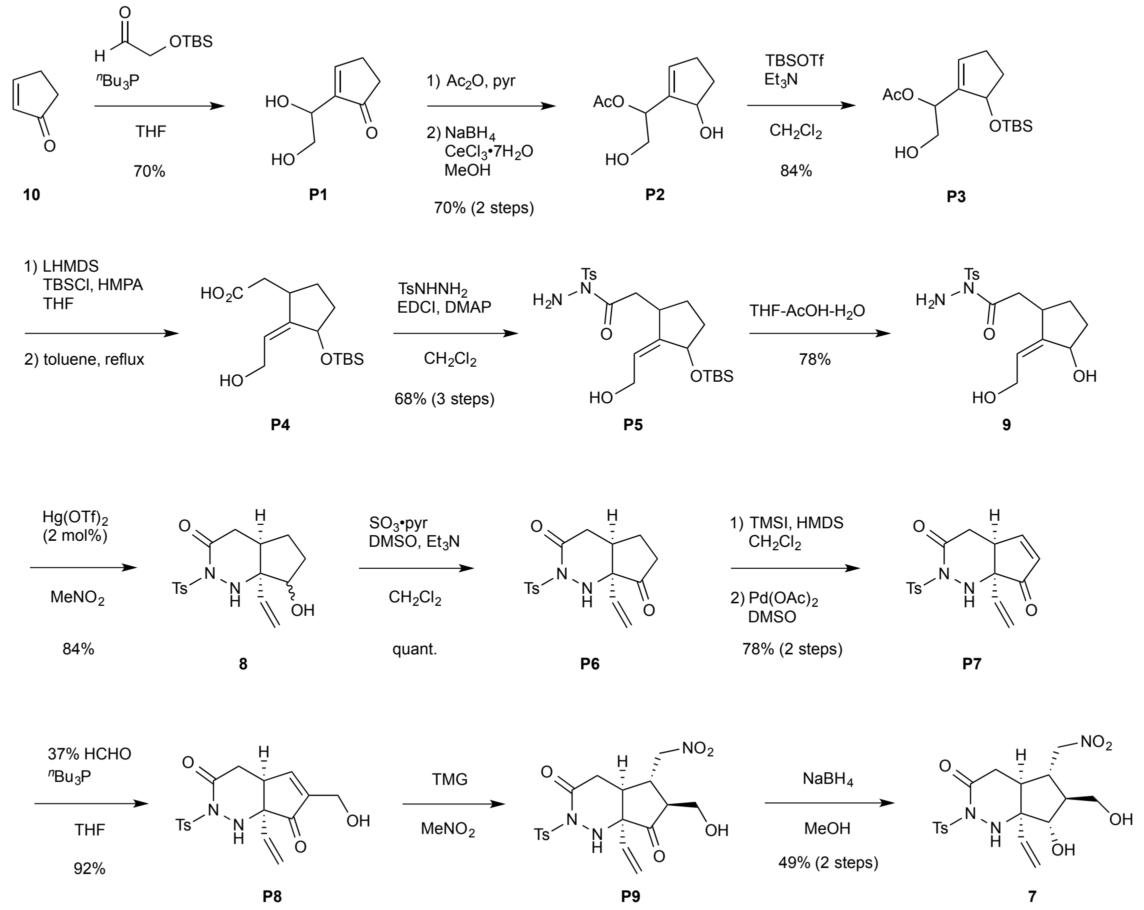

Supplementary Figure 1. Previous Synthesis of Compound **7**[1]

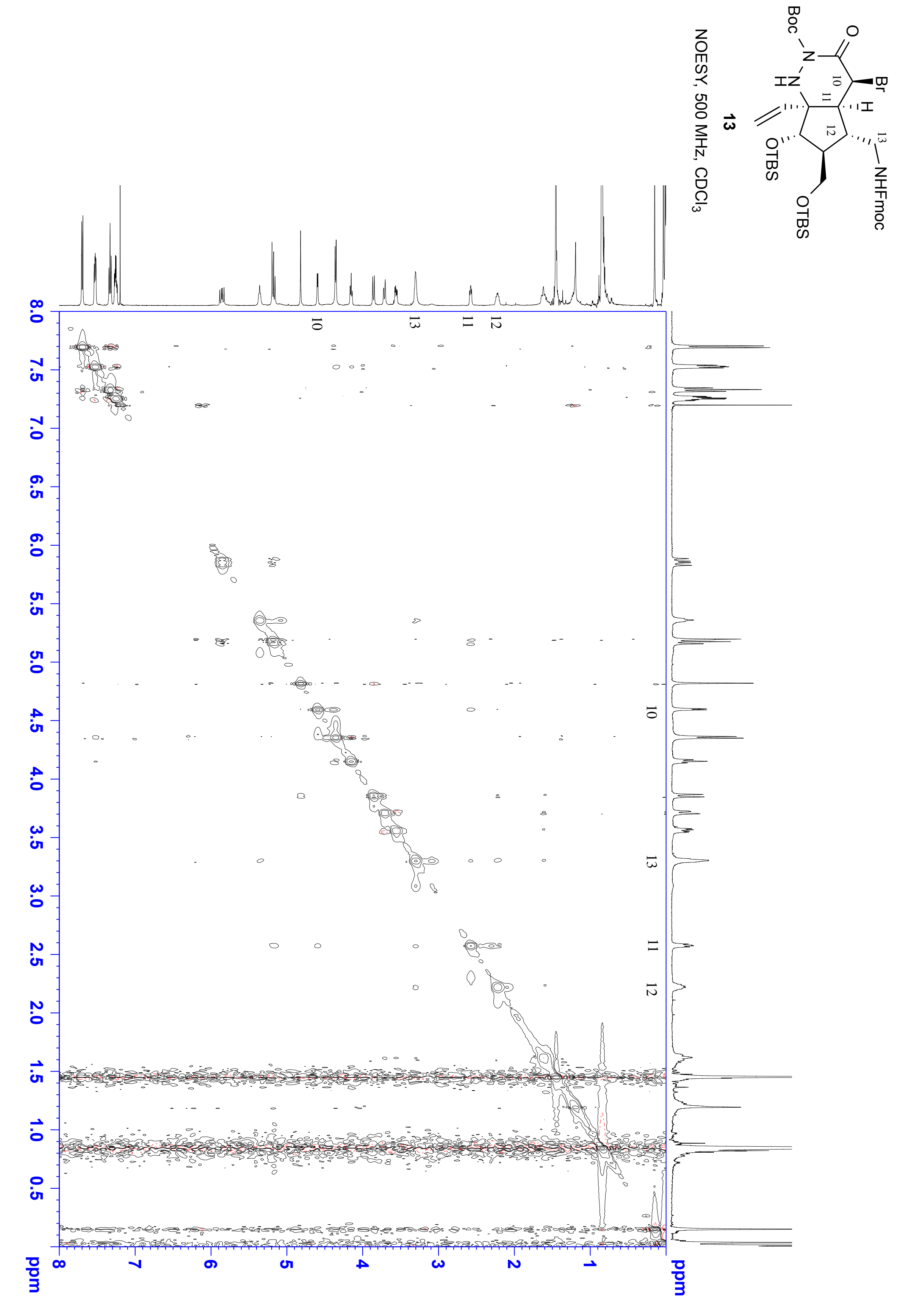

Supplementary Figure 2. NOESY spectrum of **13**

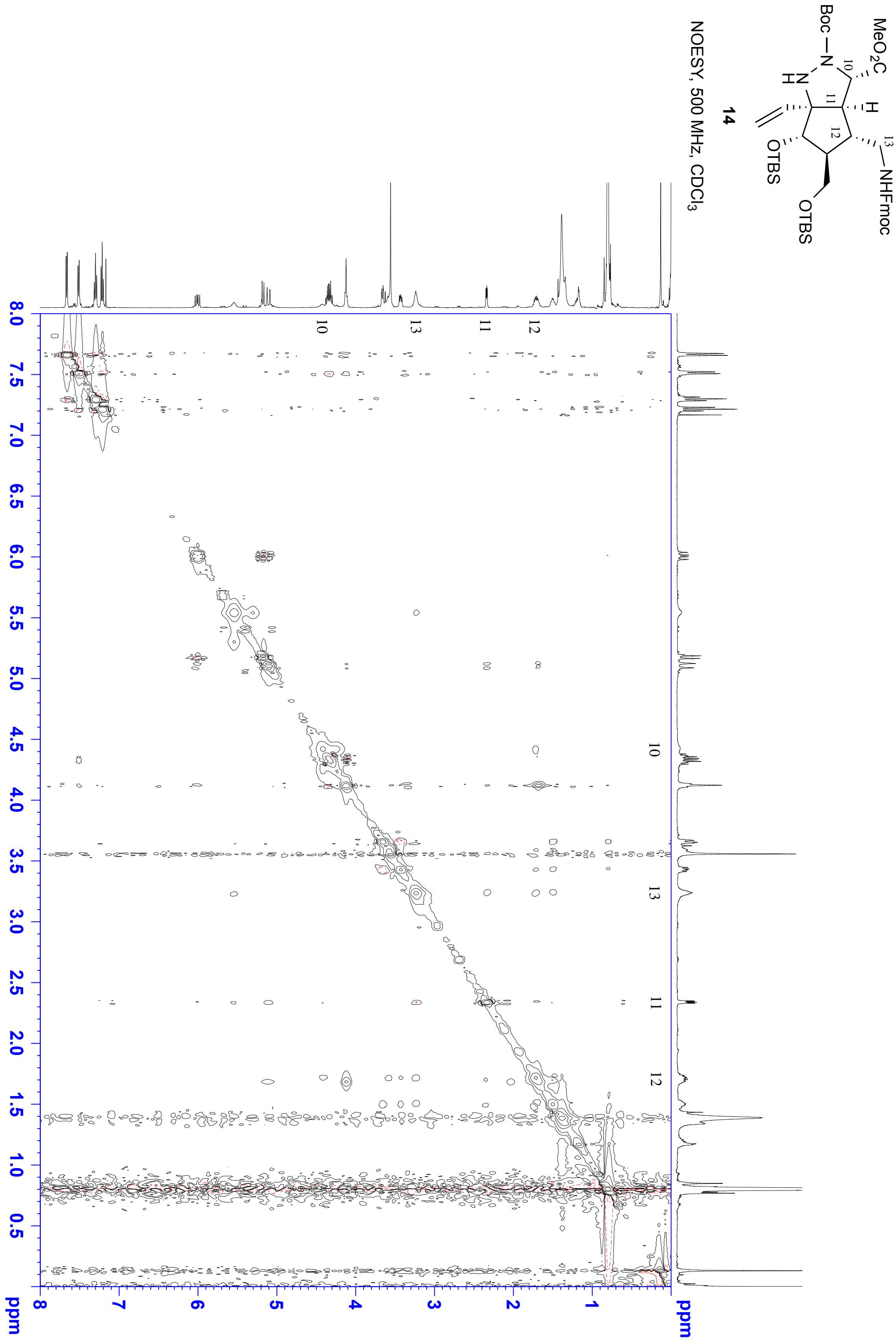

Supplementary Figure 3. NOEY spectrum of **14**

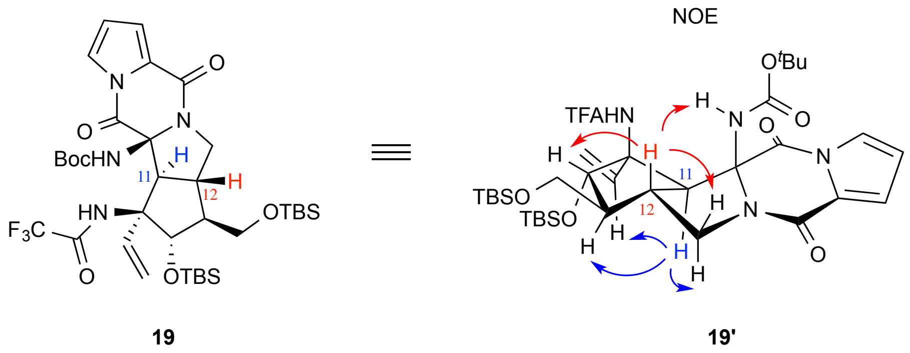

Supplementary Figure 4. NOE Analysis of Compound **19**

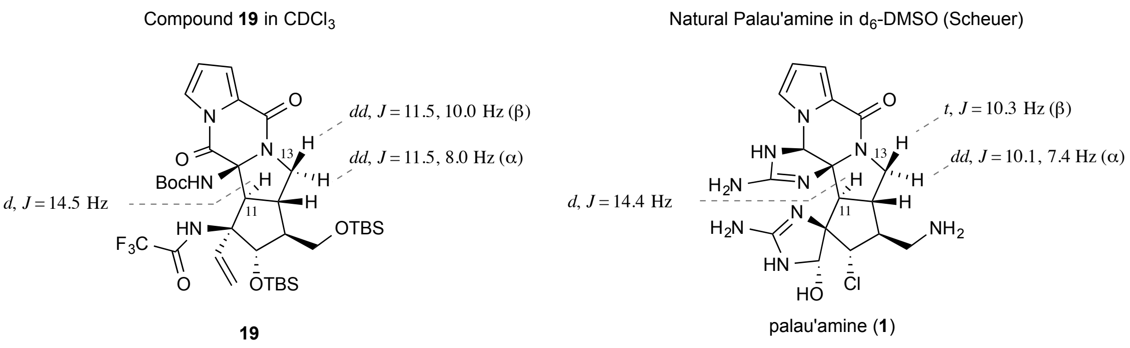

Supplementary Figure 5. Comparison of Coupling Constant of Compound **19** and Natural Palau'amine (**1**)

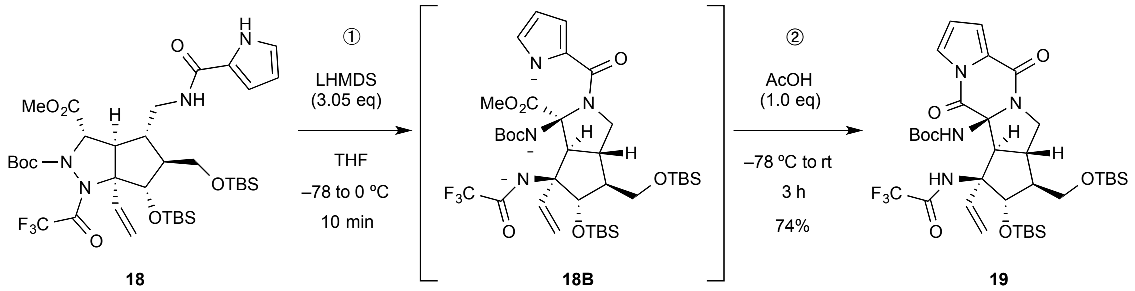

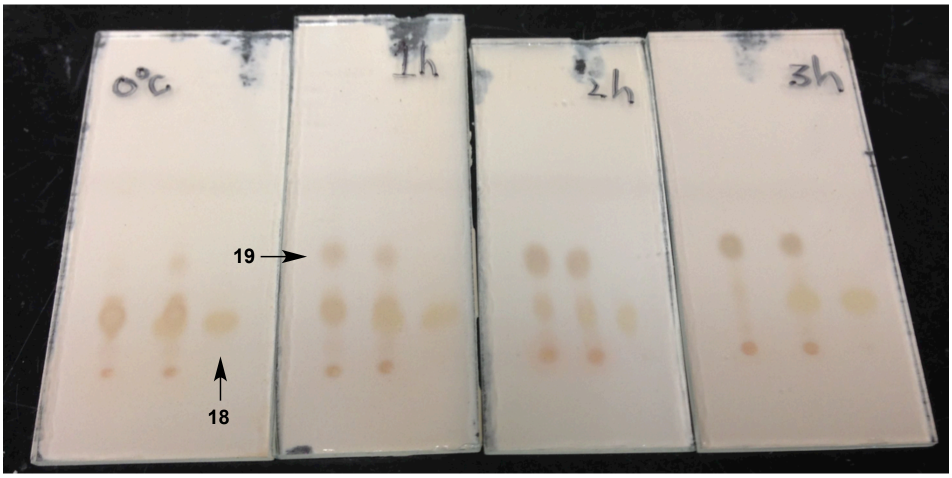

TLC (hexane/EtOAc = 3:1)

Supplementary Figure 6. TLC Analysis in the Conversion of Compound **18** to **19**

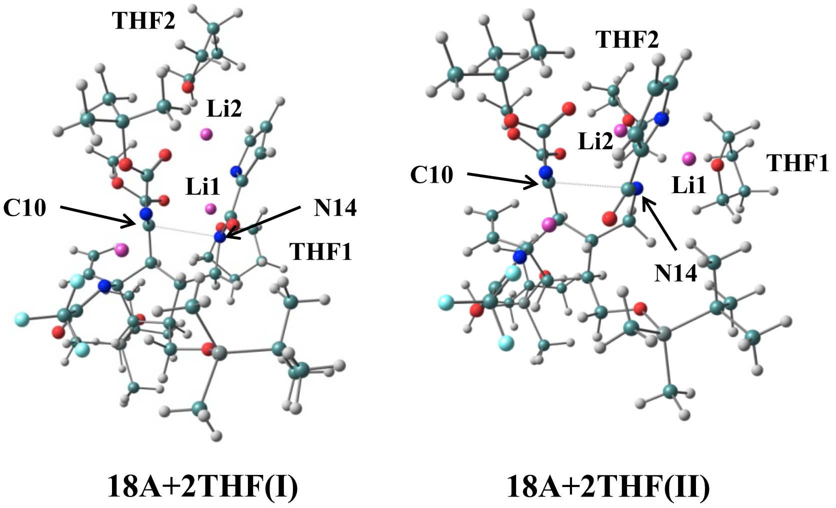

Supplementary Figure 7. Optimized structures of **18A+2THF(I)** and **18A+2THF(II)**.

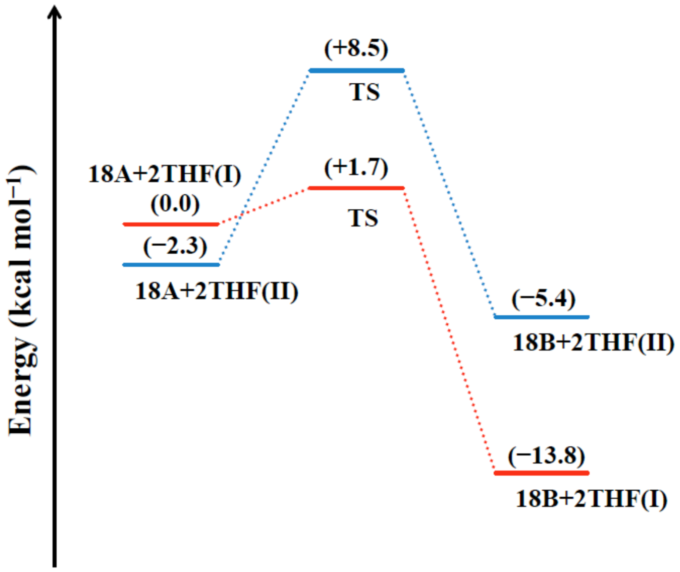

Supplementary Figure 8. Potential energy profiles for the cyclization reaction of **18A+2THF(I)→18B+2THF(I)** and **18A+2THF(II)→18B+2THF(II)**. The potential energies (in kcal/mol) relative to **18A+2THF(I)** are shown in parenthesis.

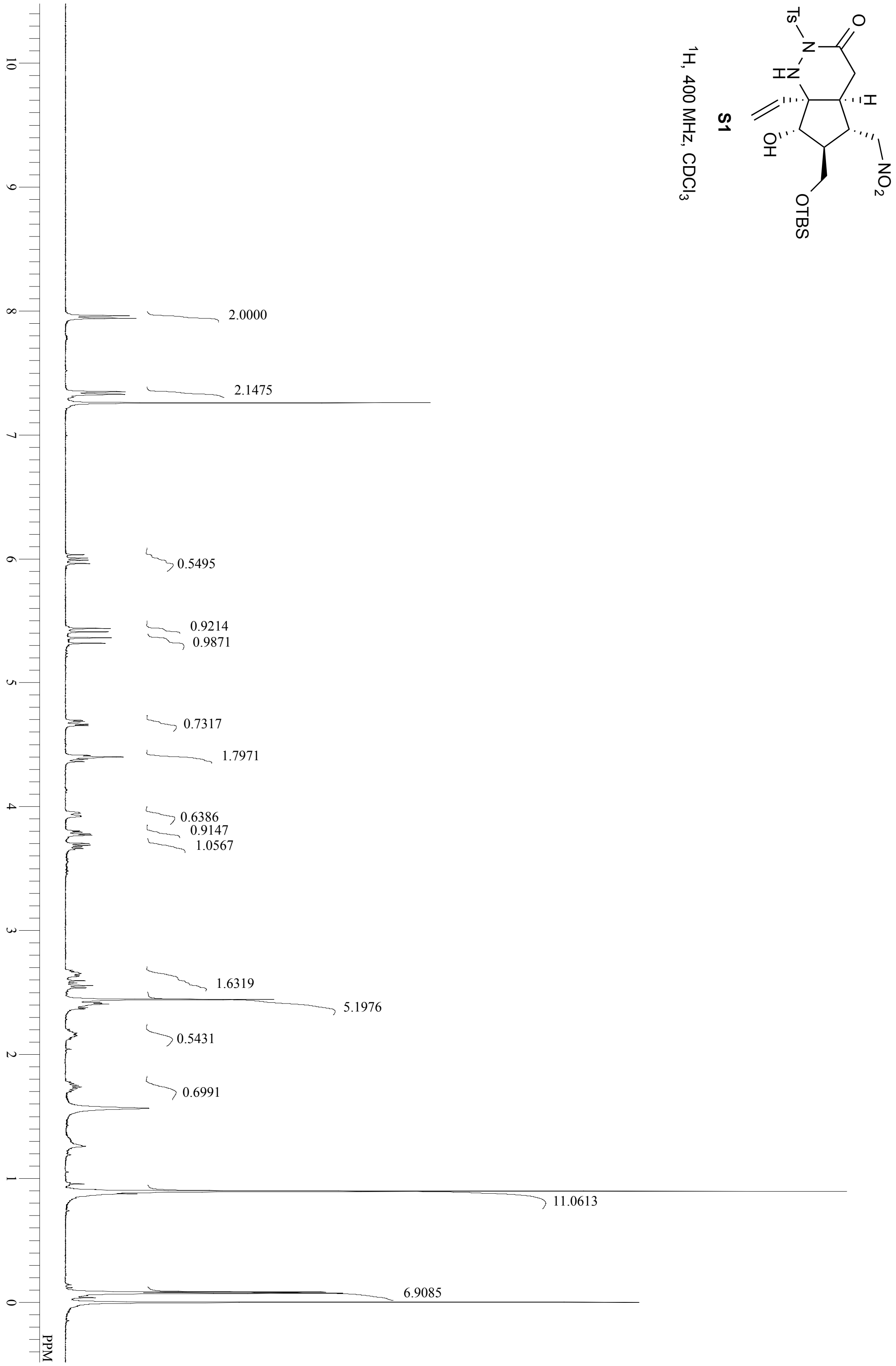

Supplementary Figure 9. ^{1}H-NMR spectrum of **S1**

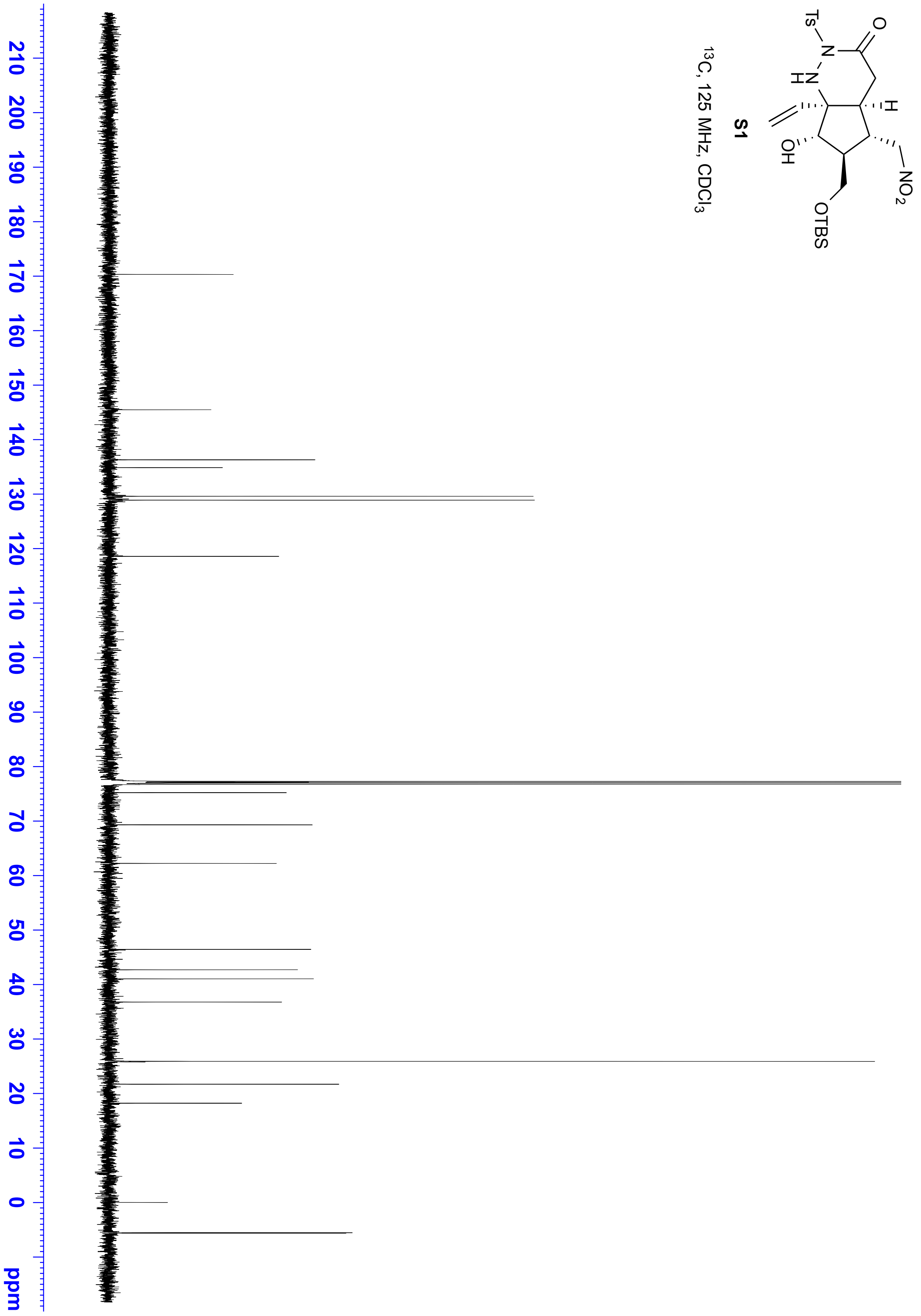

Supplementary Figure 10. ^{13}C-NMR spectrum of **S1**

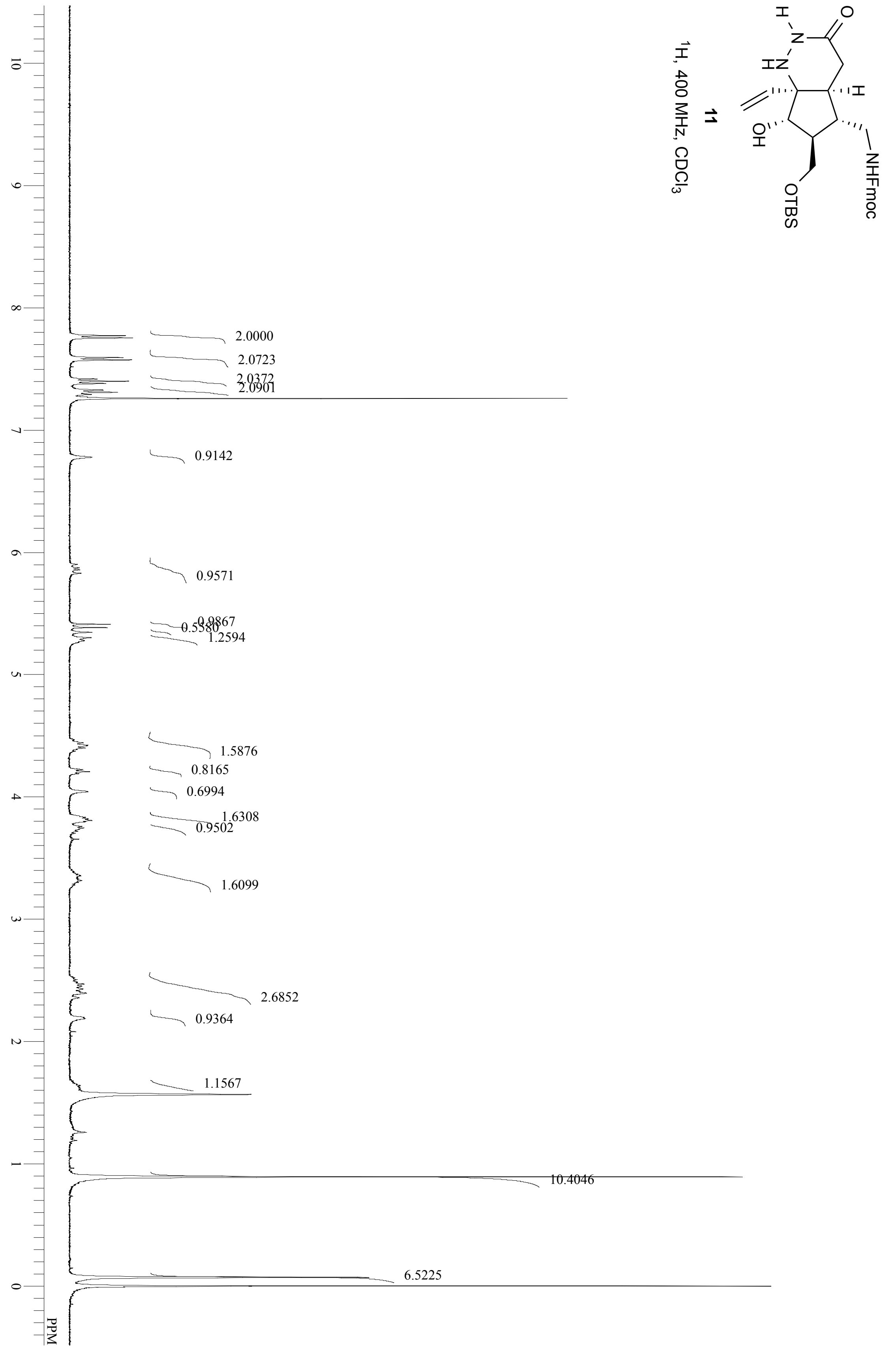

Supplementary Figure 11. ^{1}H-NMR spectrum of **11**

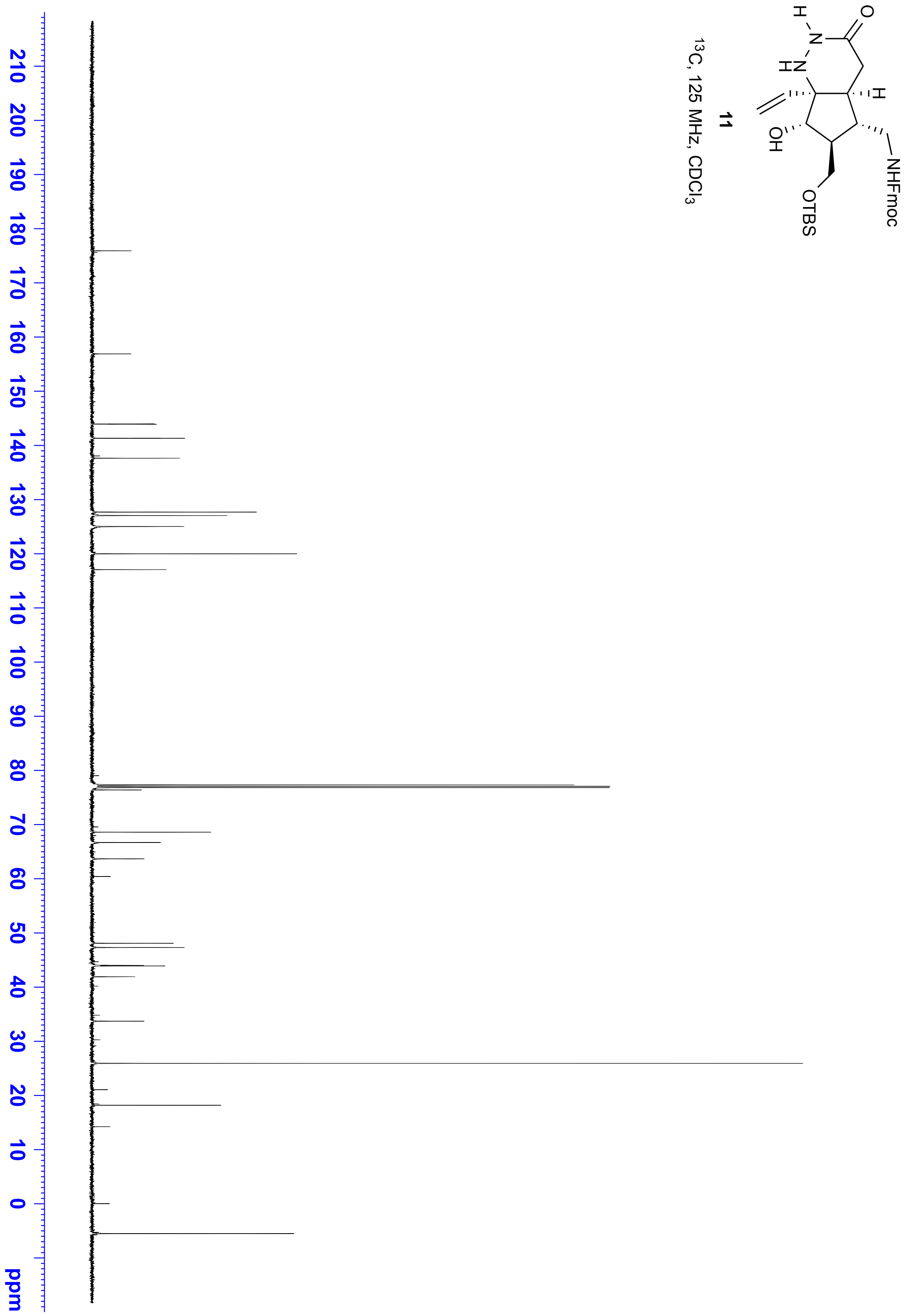

Supplementary Figure 12. ^{13}C-NMR spectrum of **11**

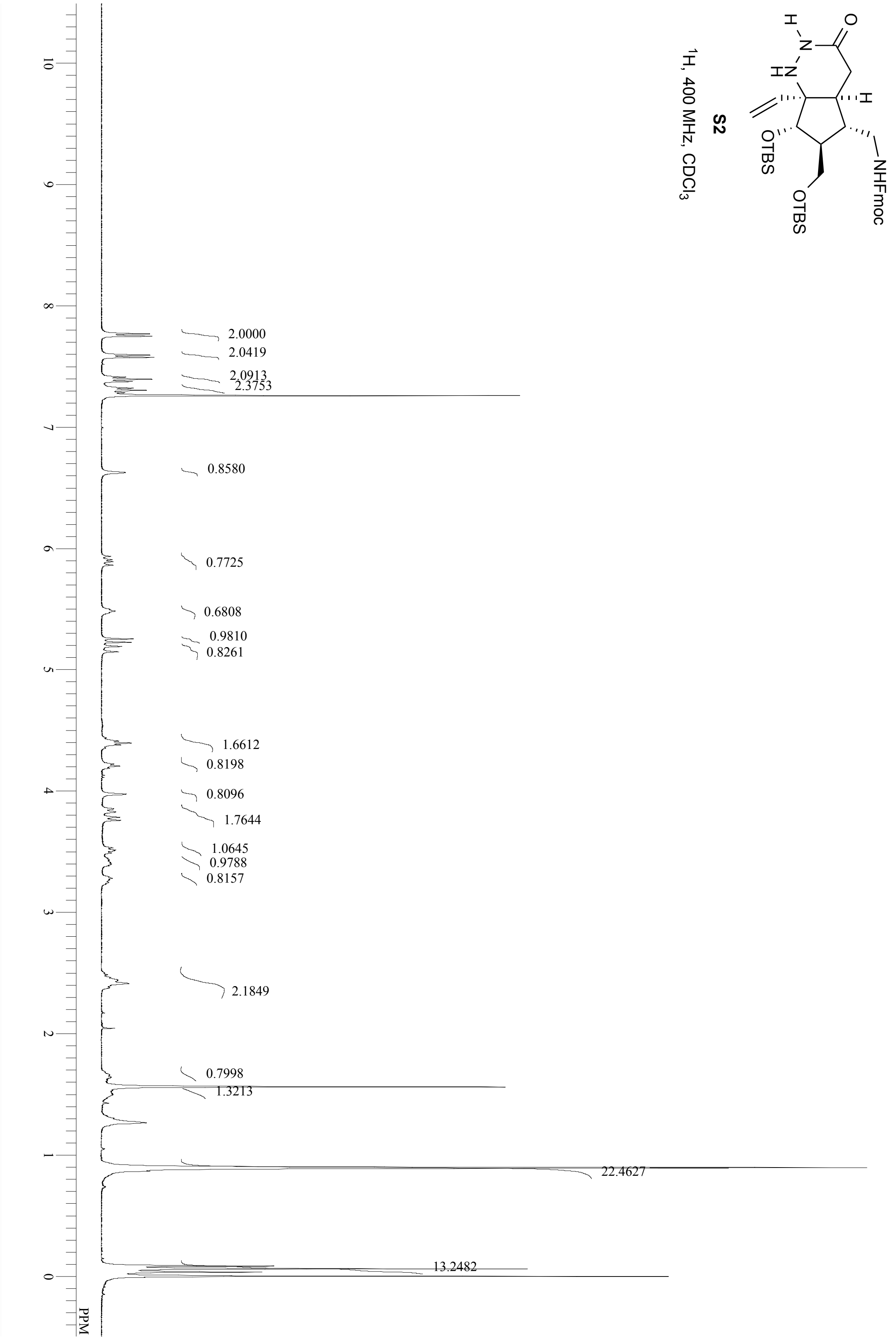

Supplementary Figure 13. ^{1}H-NMR spectrum of **S2**

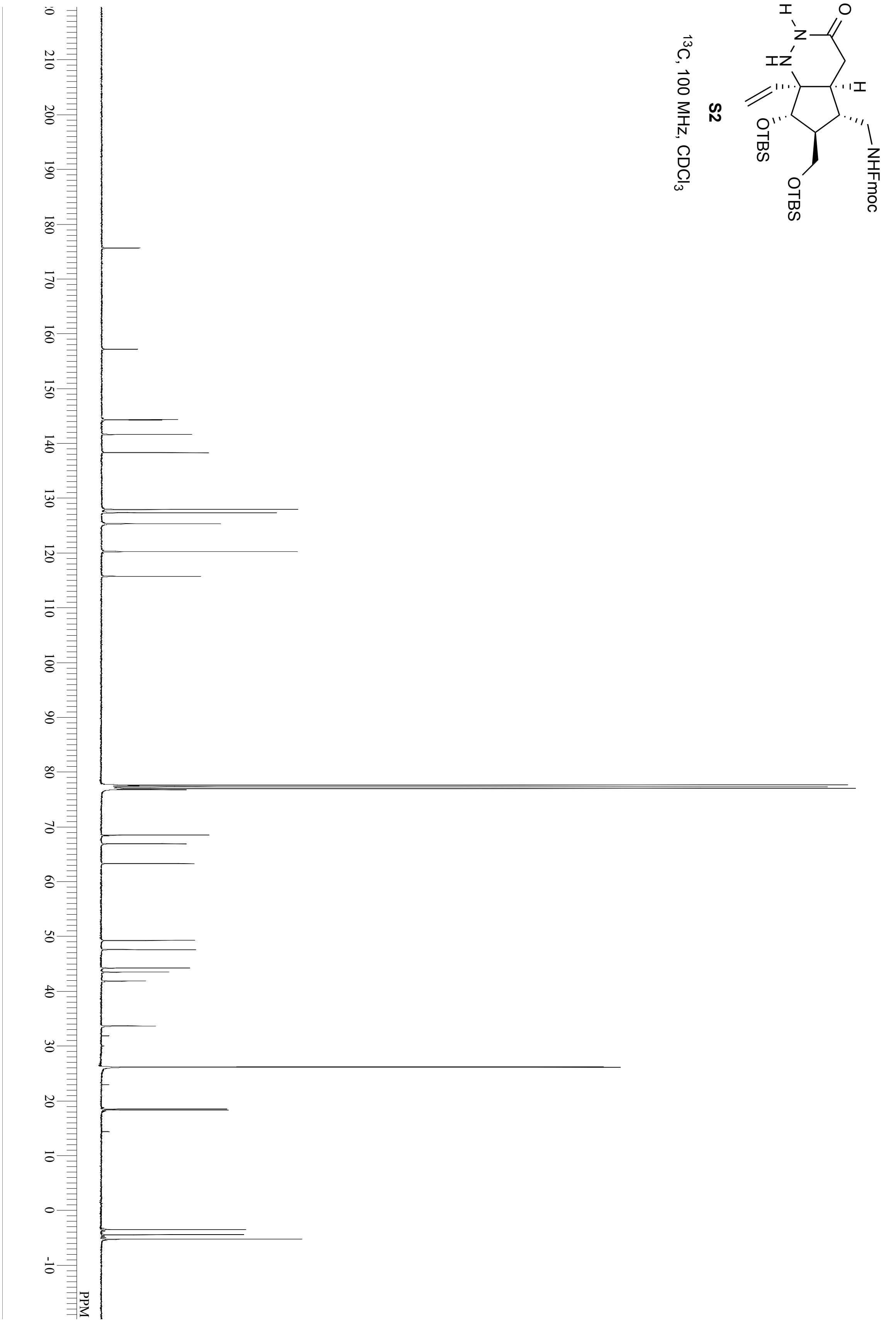

Supplementary Figure 14. ^{13}C-NMR spectrum of **S2**

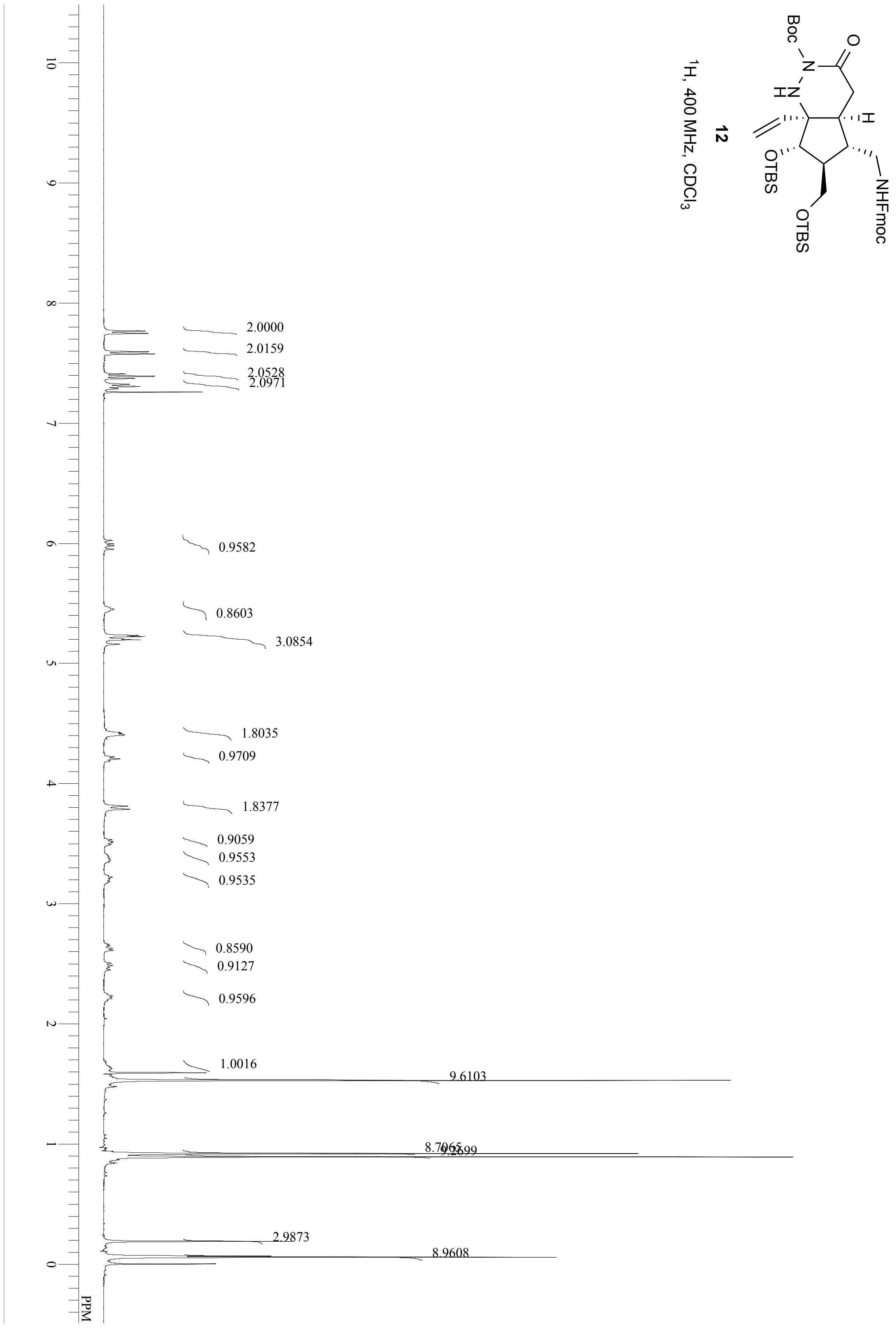

Supplementary Figure 15. ^{1}H-NMR spectrum of **12**

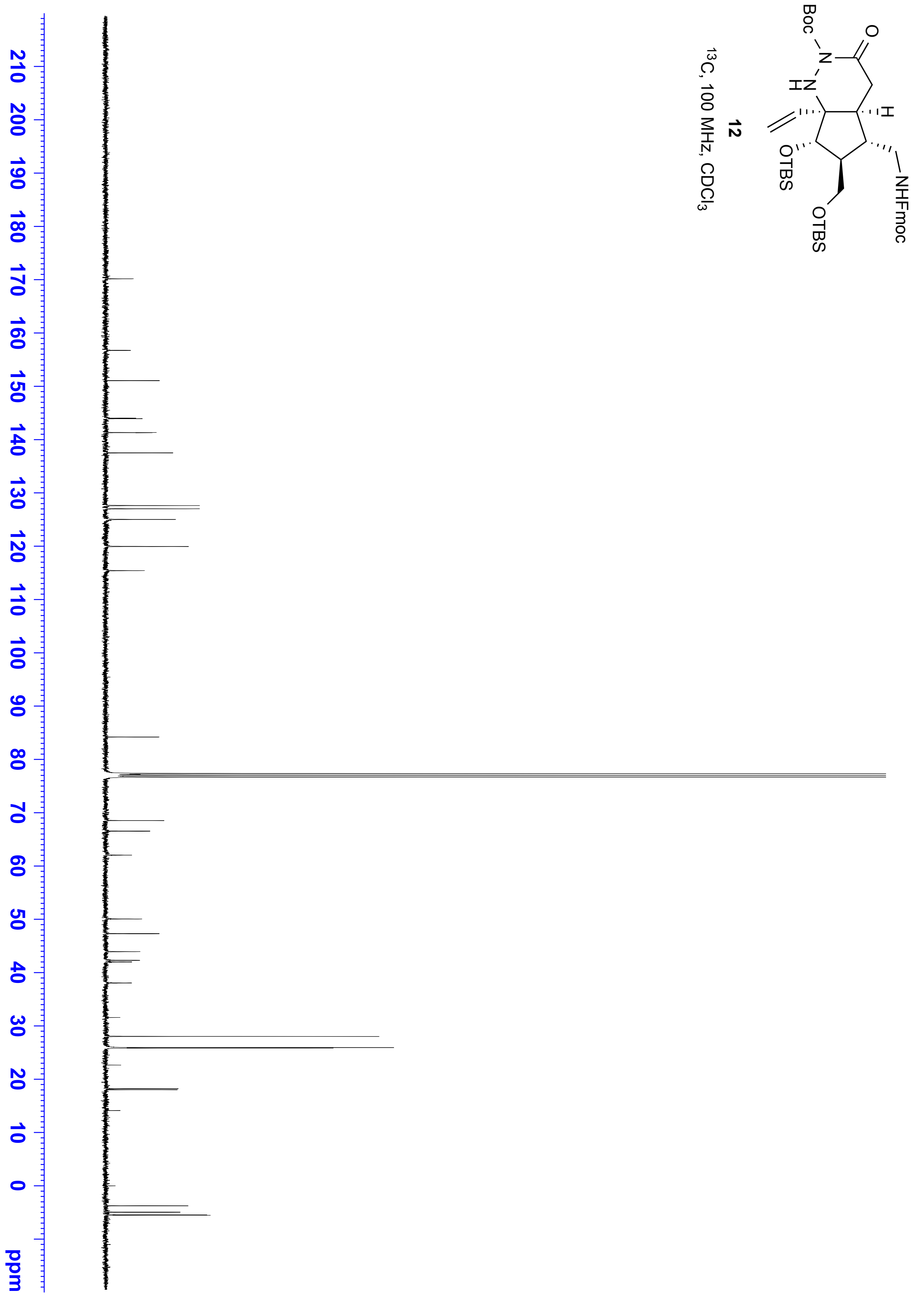

Supplementary Figure 16. ^{13}C-NMR spectrum of **12**

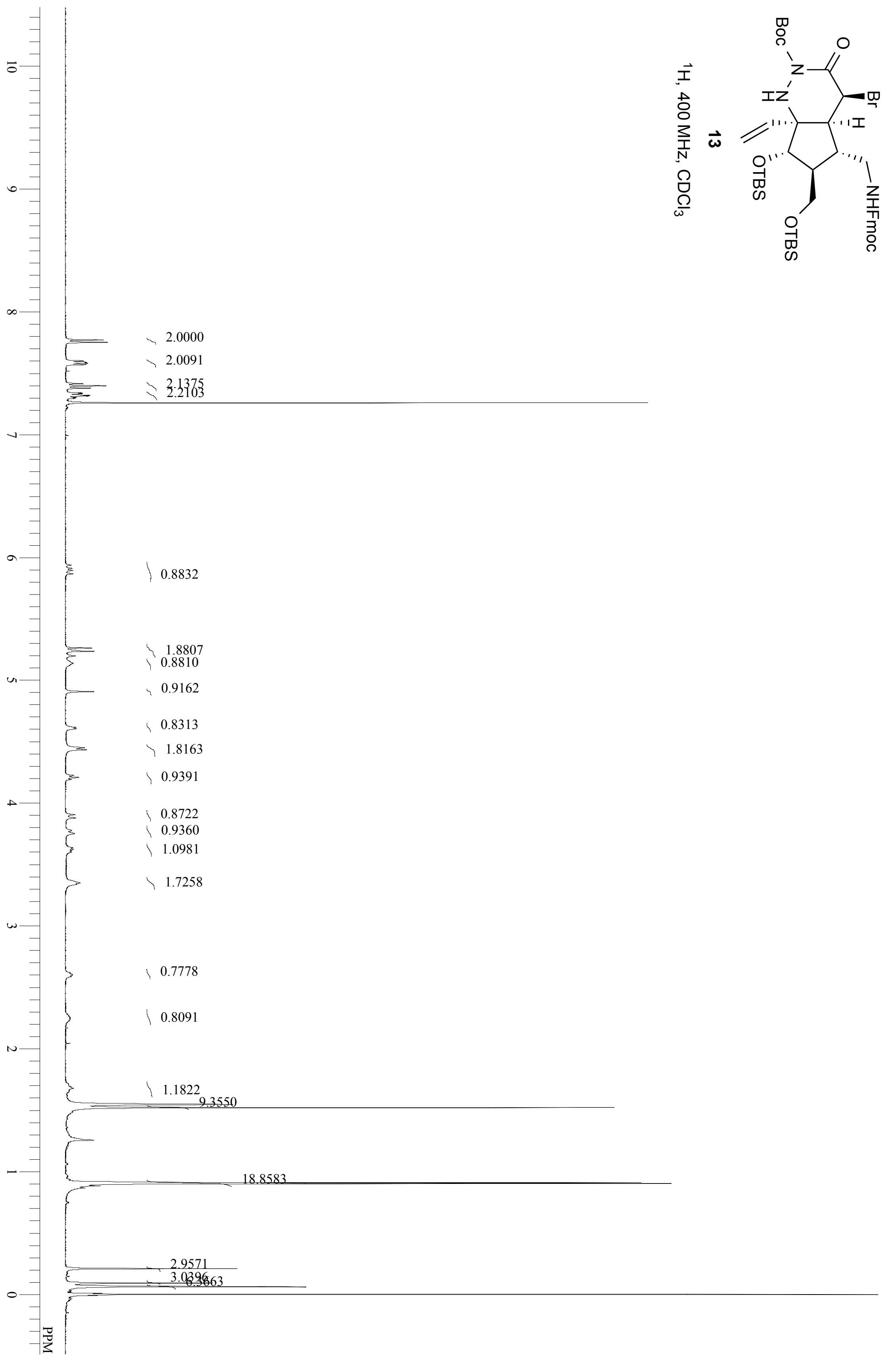

Supplementary Figure 17. ^{1}H-NMR spectrum of **13**

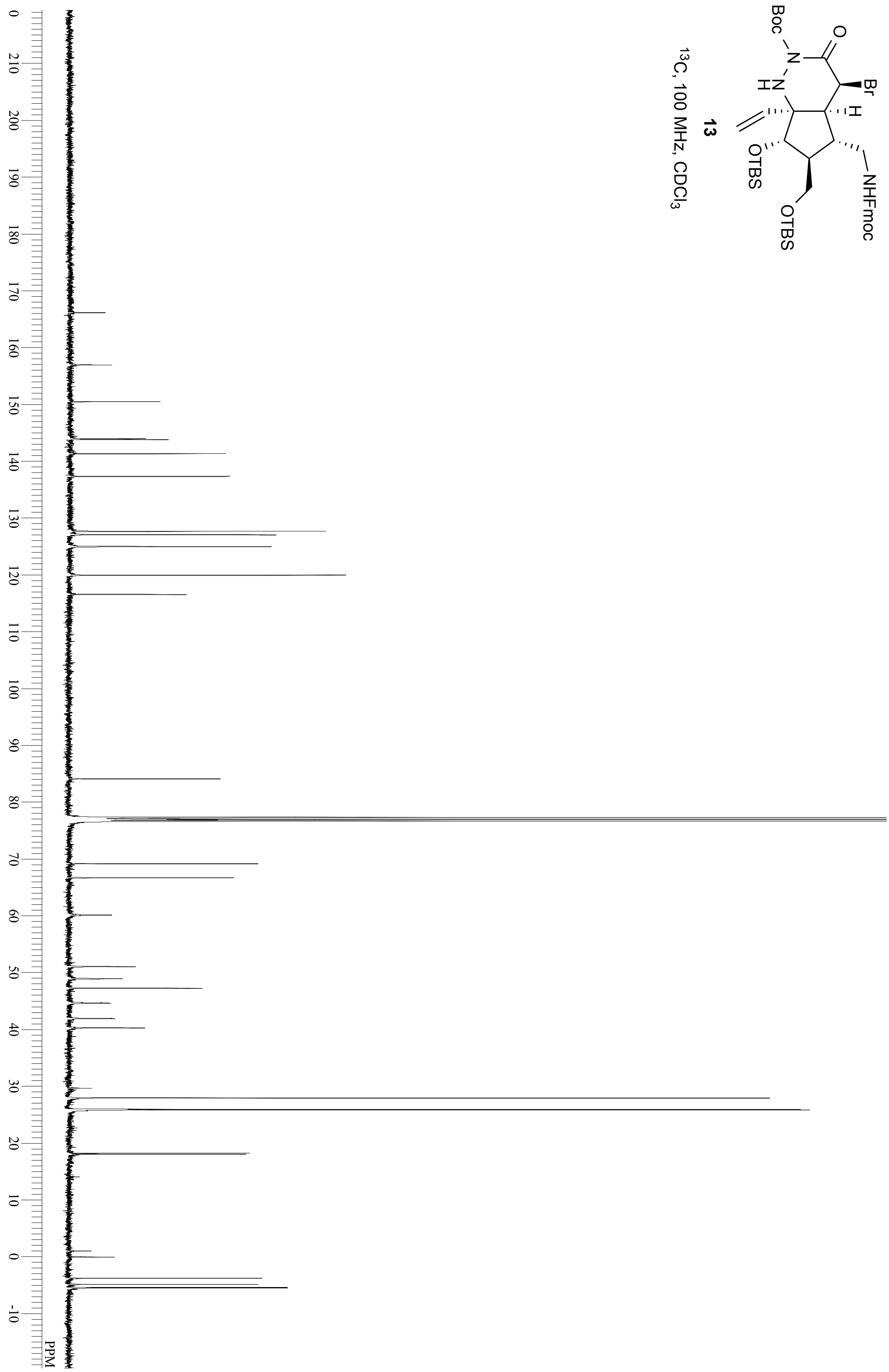

Supplementary Figure 18. ^{13}C-NMR spectrum of **13**

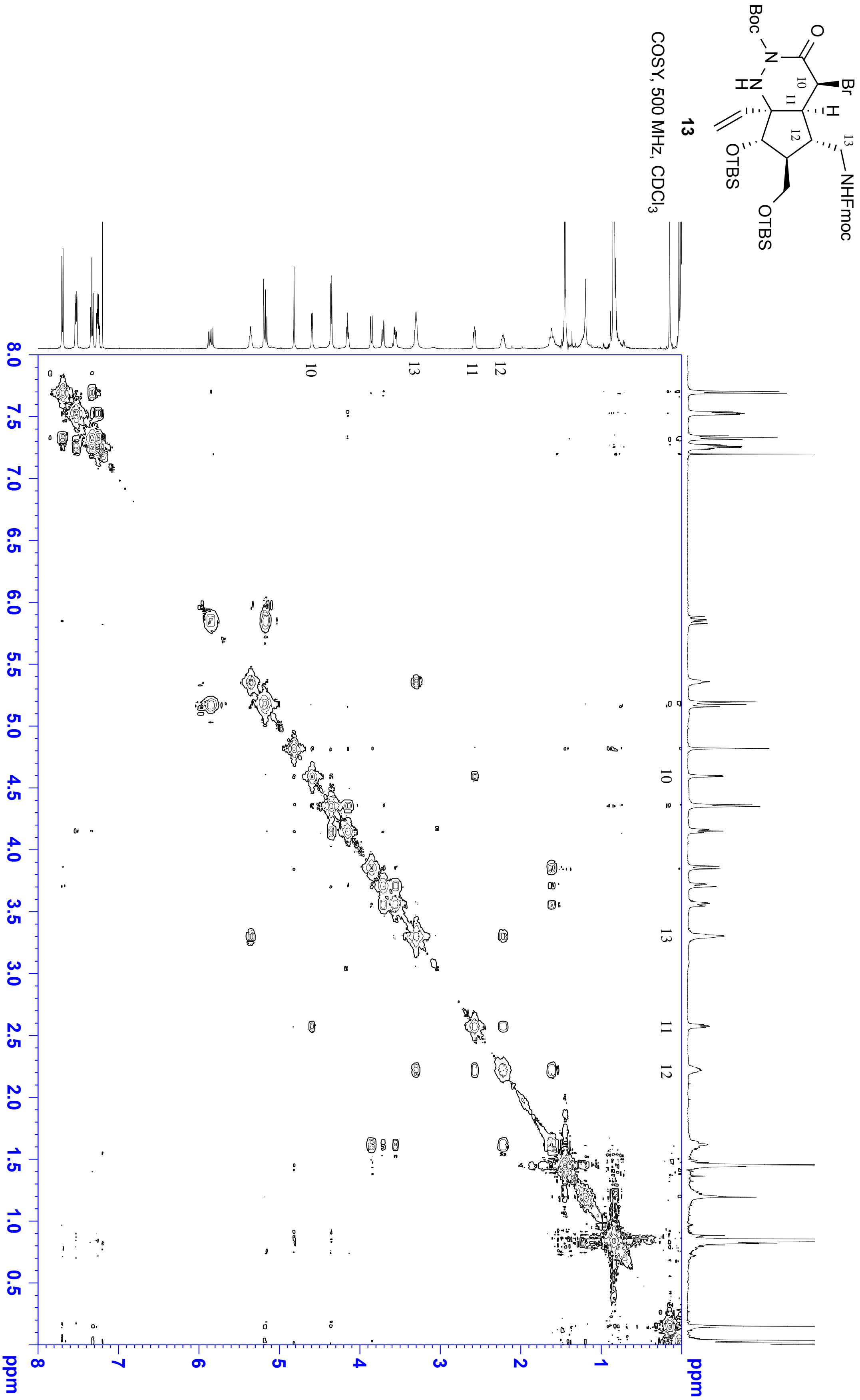

Supplementary Figure 19. COSY spectrum of **13**

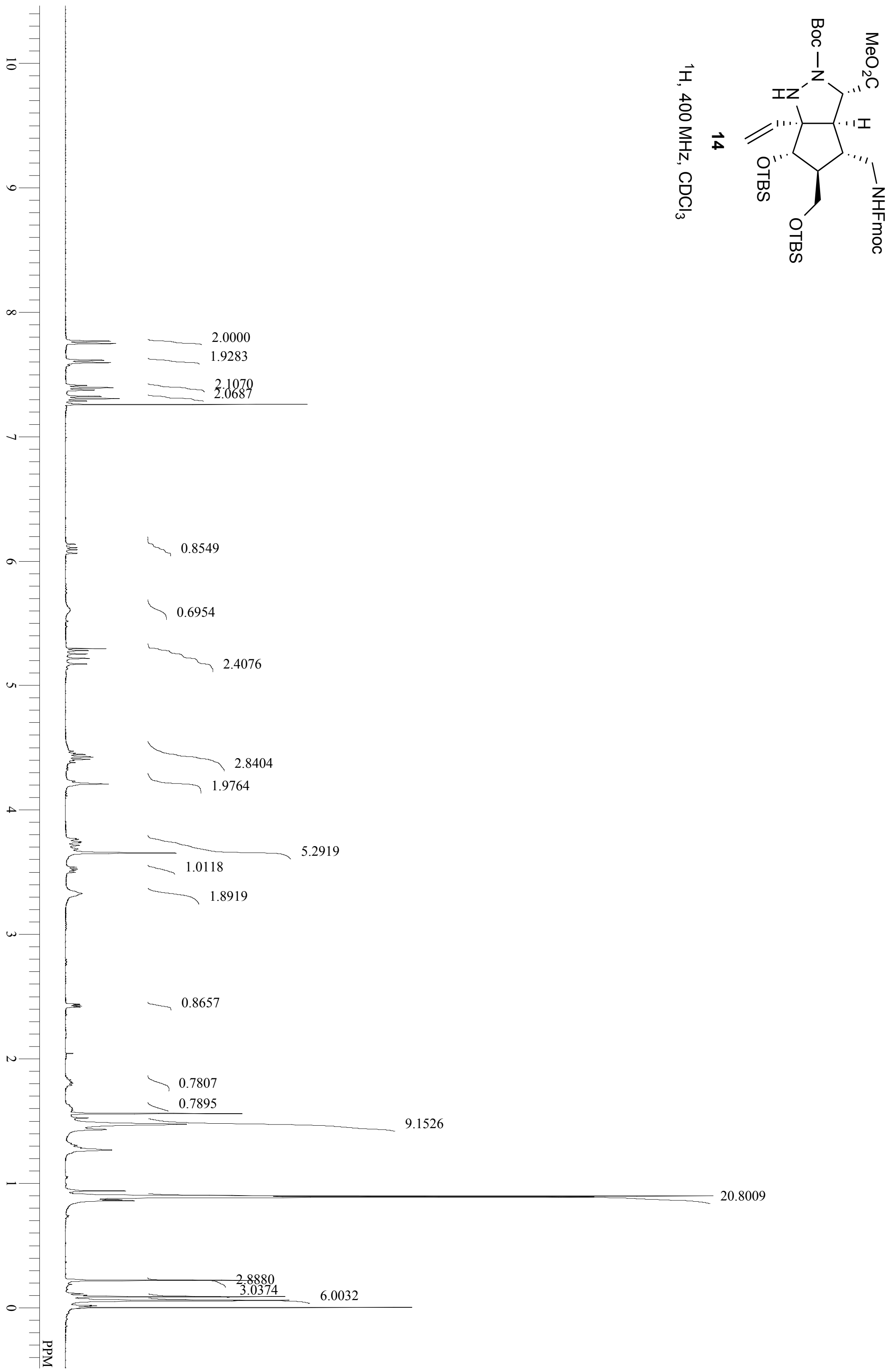

Supplementary Figure 20. ^{1}H-NMR spectrum of **14**

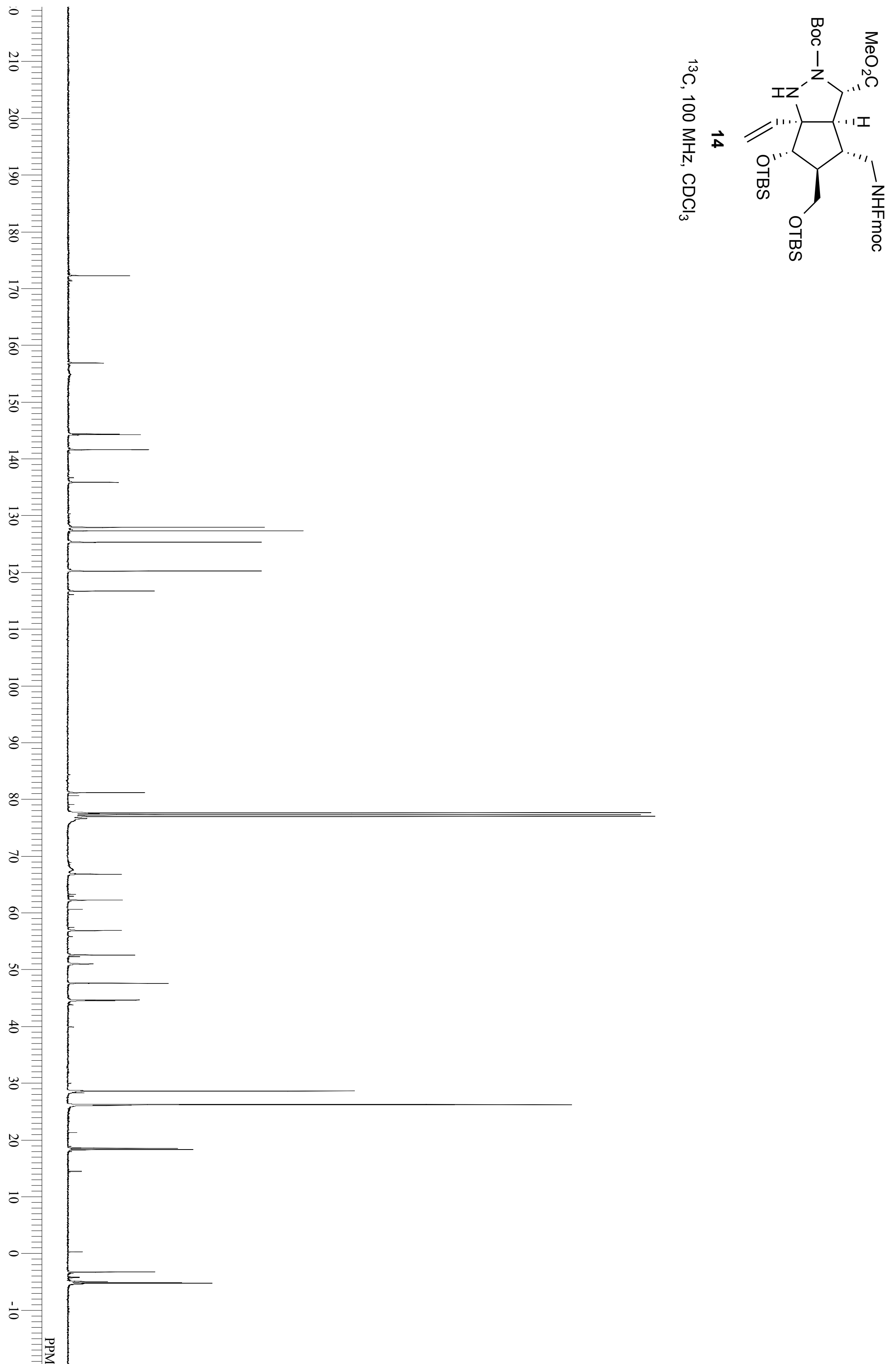

Supplementary Figure 21. ^{13}C-NMR spectrum of **14**

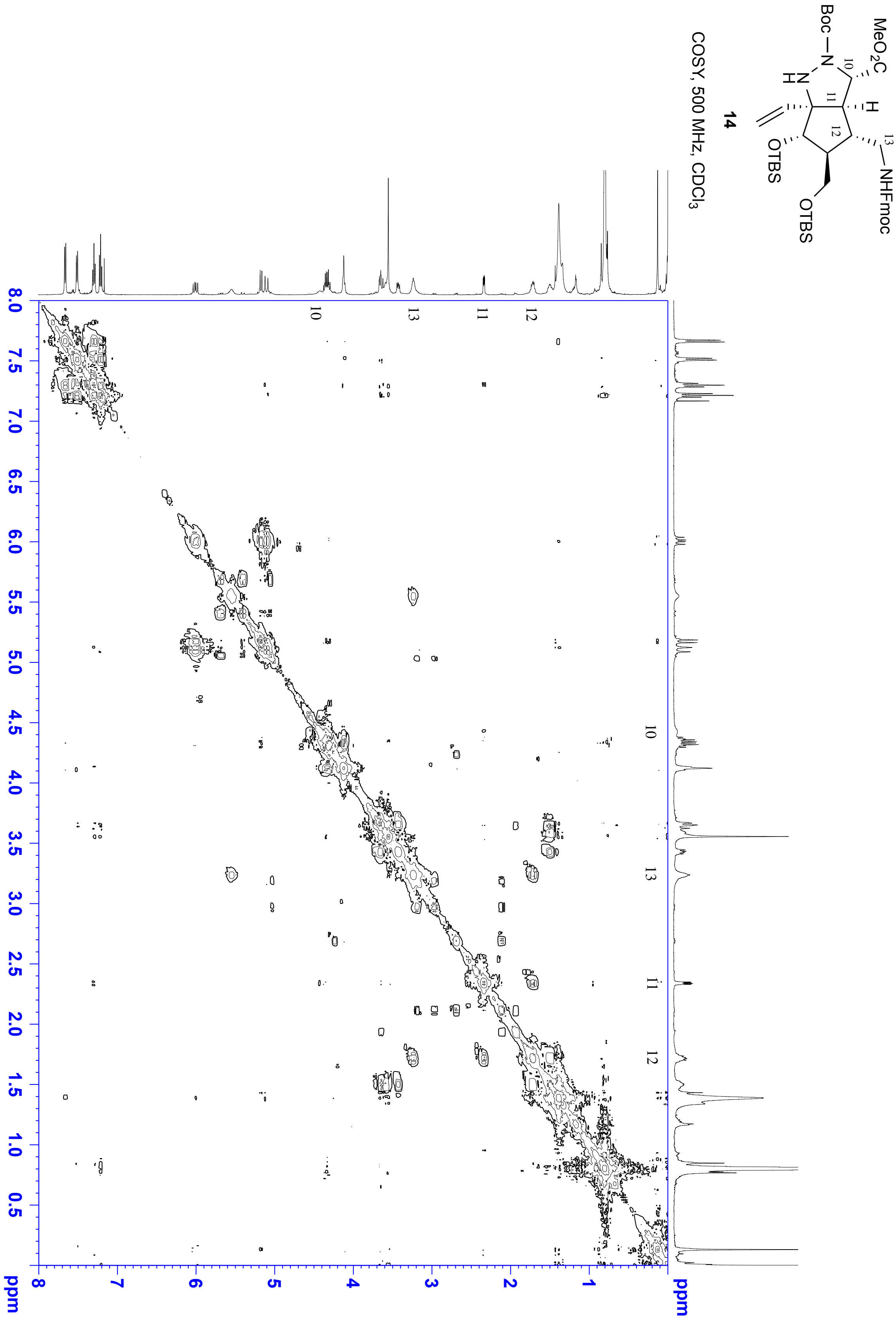

Supplementary Figure 22. COSY spectrum of **14**

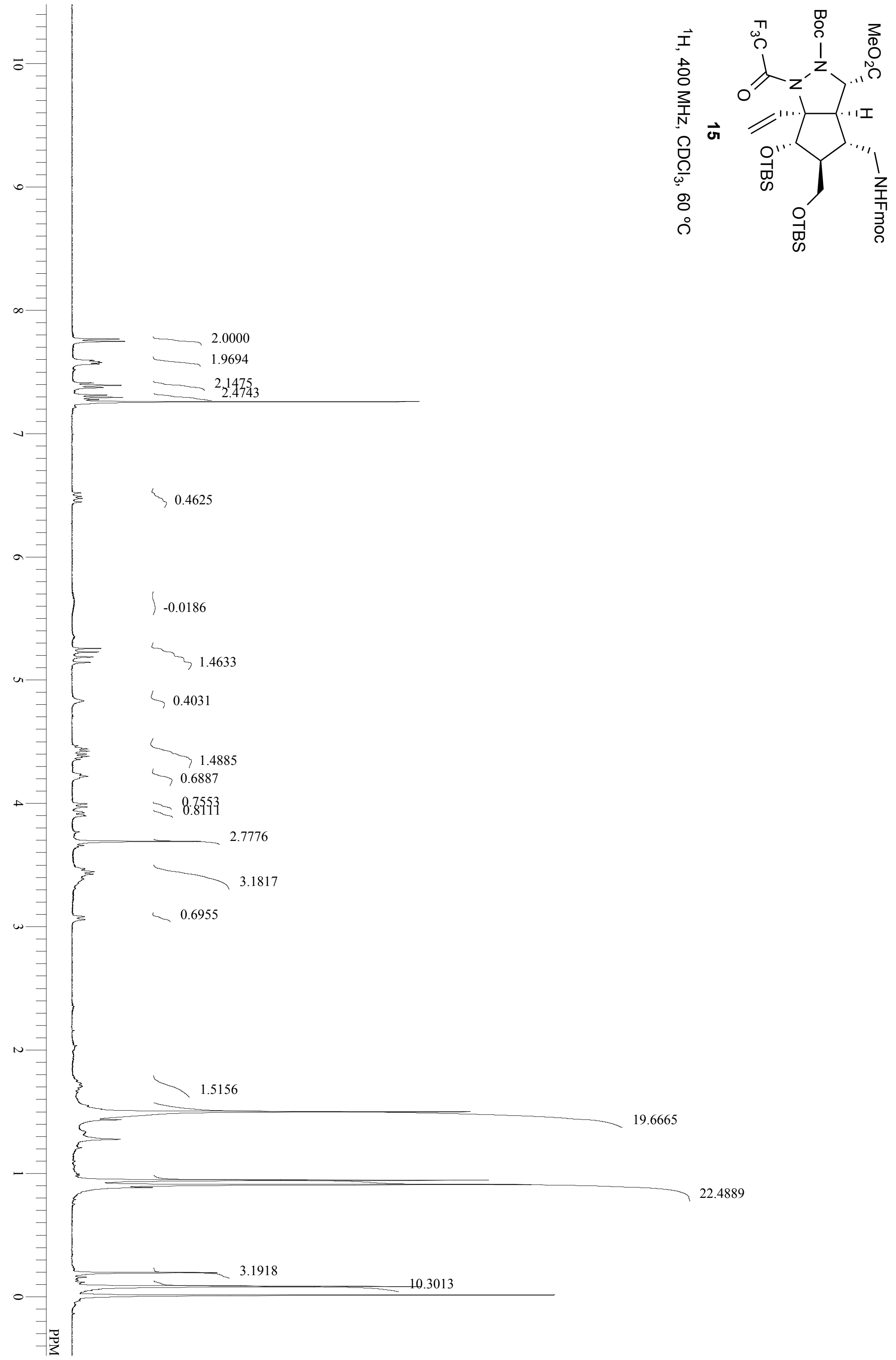

Supplementary Figure 23. ^{1}H-NMR spectrum of **15**

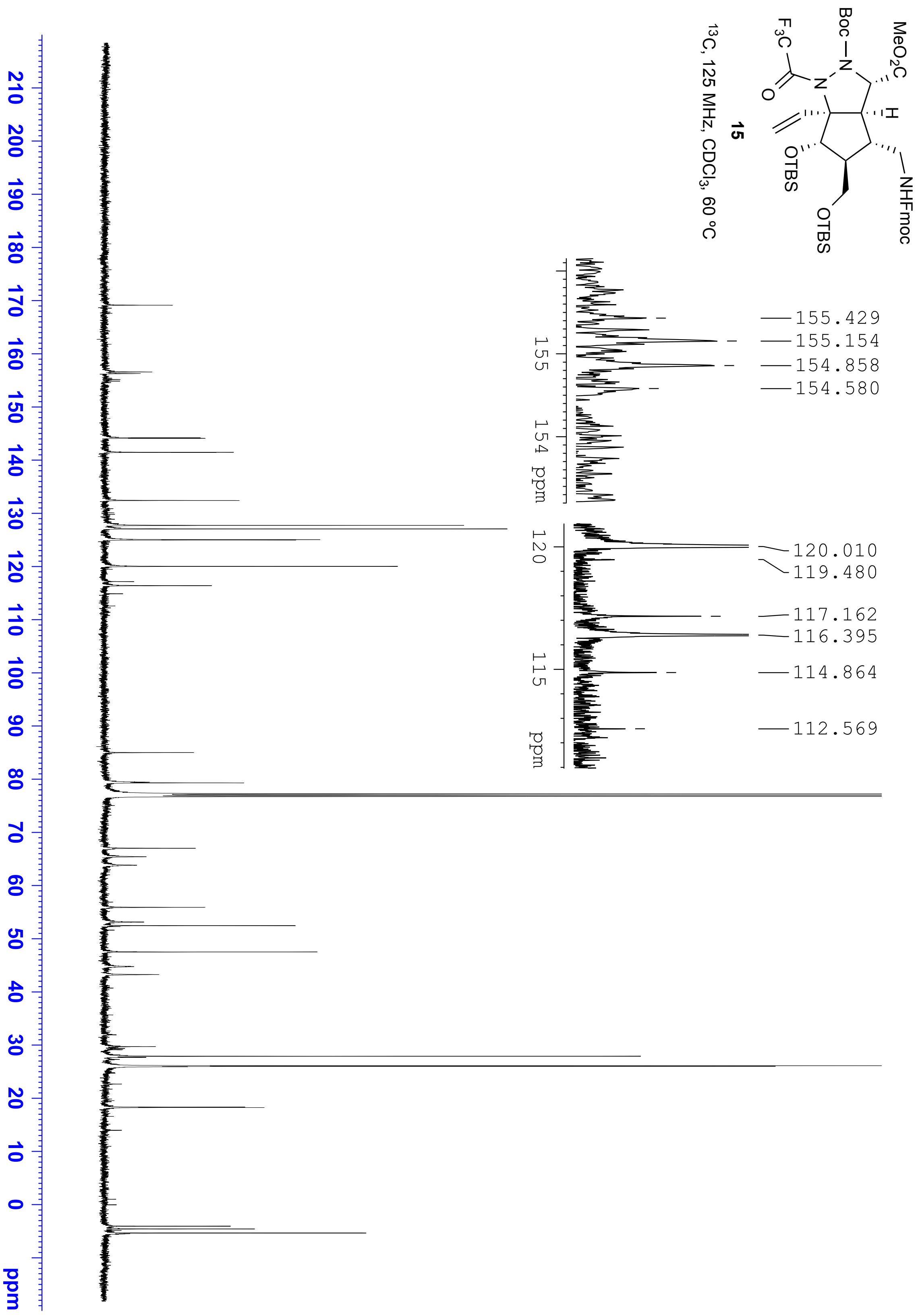

Supplementary Figure 24. ^{13}C-NMR spectrum of **15**

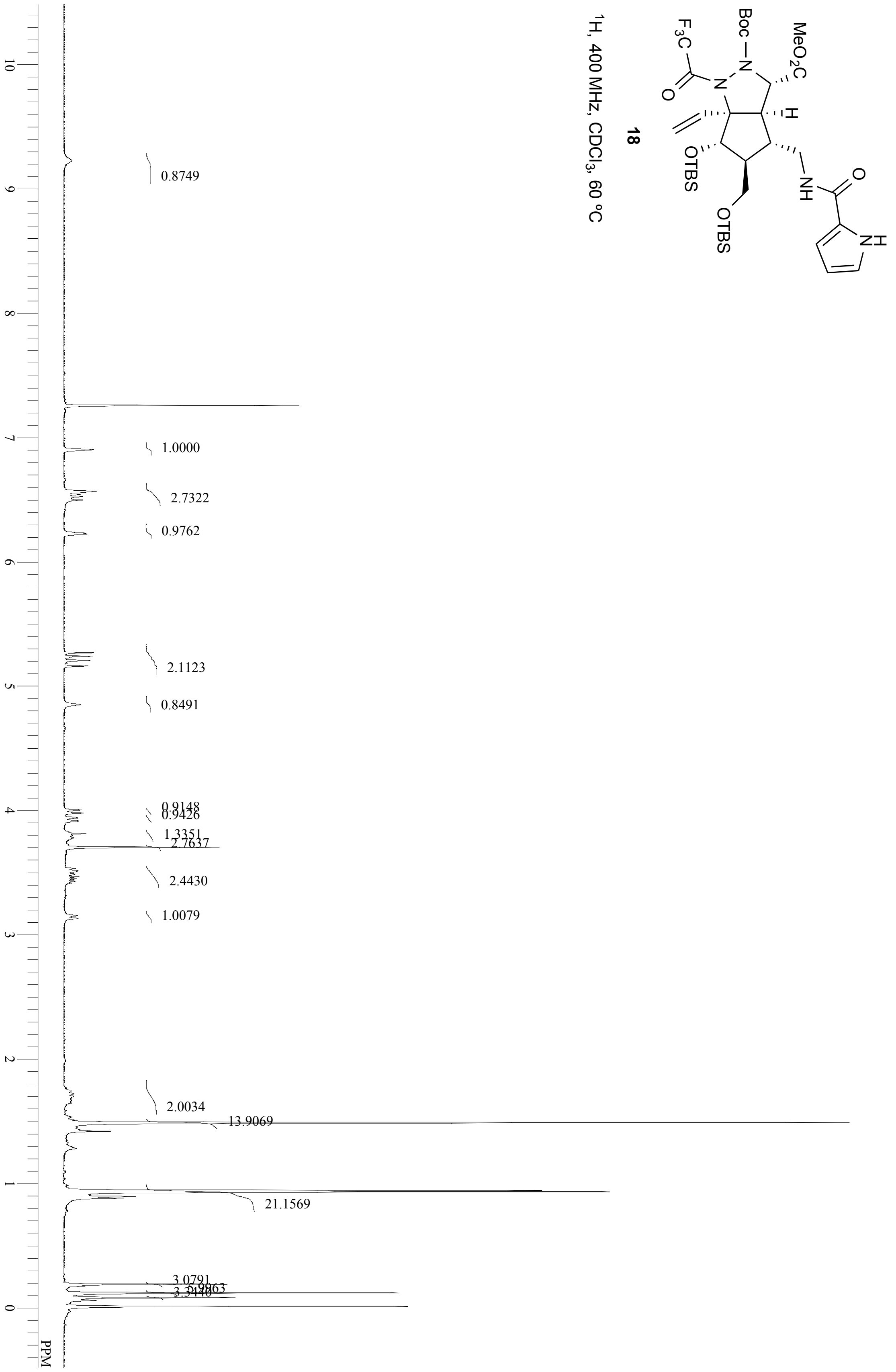

Supplementary Figure 25. ^{1}H-NMR spectrum of **18**

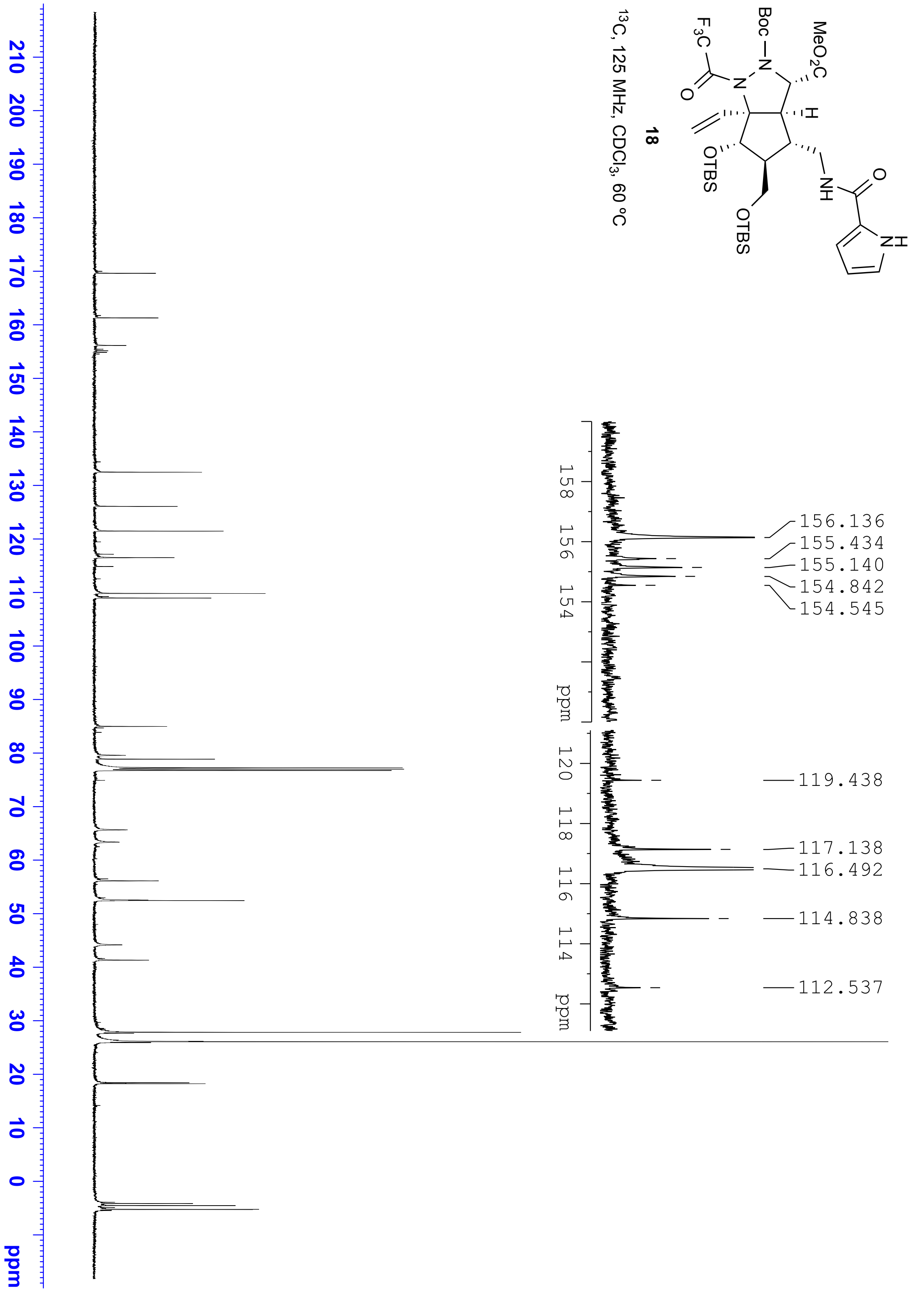

Supplementary Figure 26. ^{13}C-NMR spectrum of **18**

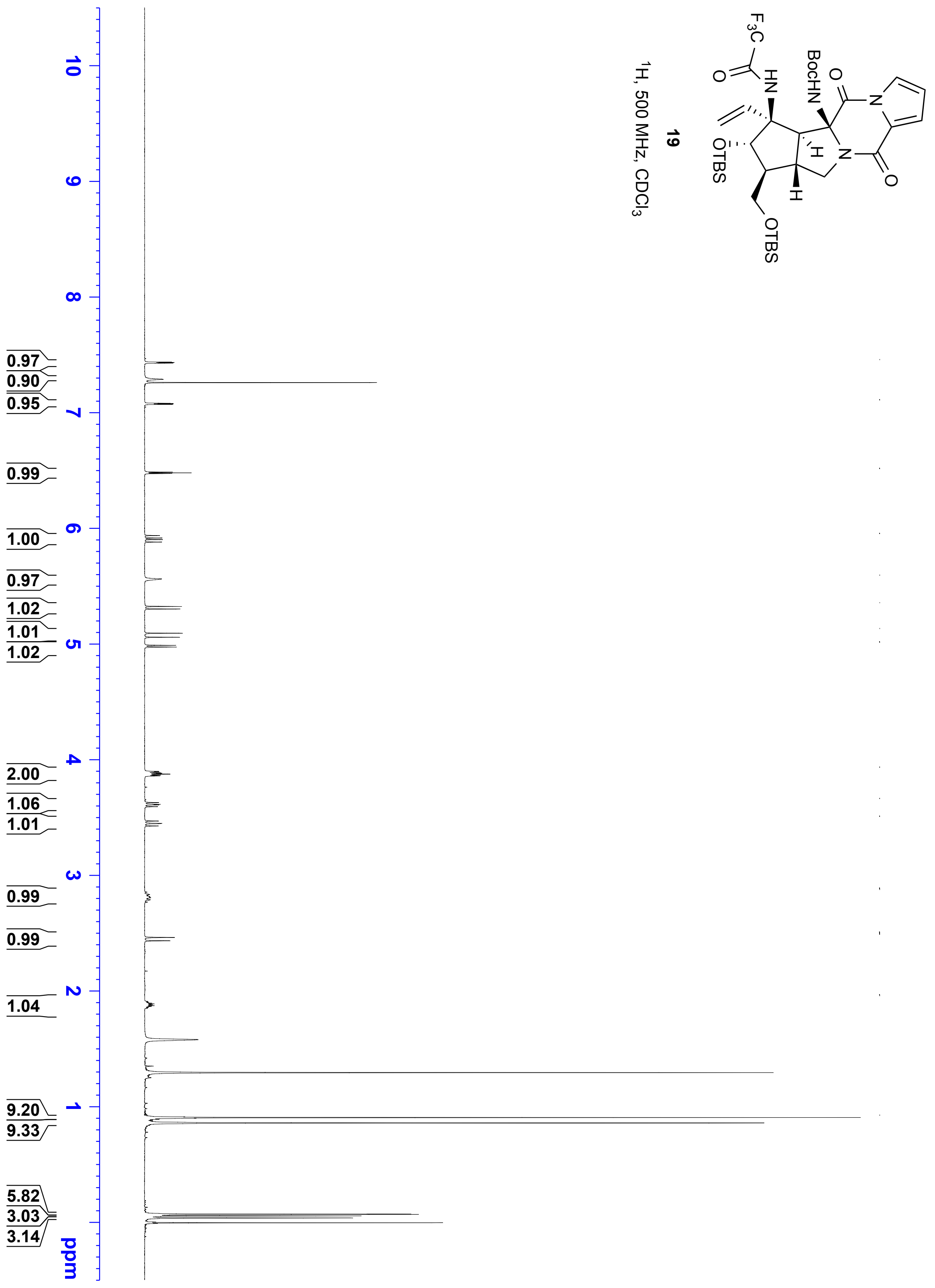

Supplementary Figure 27. ^{1}H-NMR spectrum of **19**

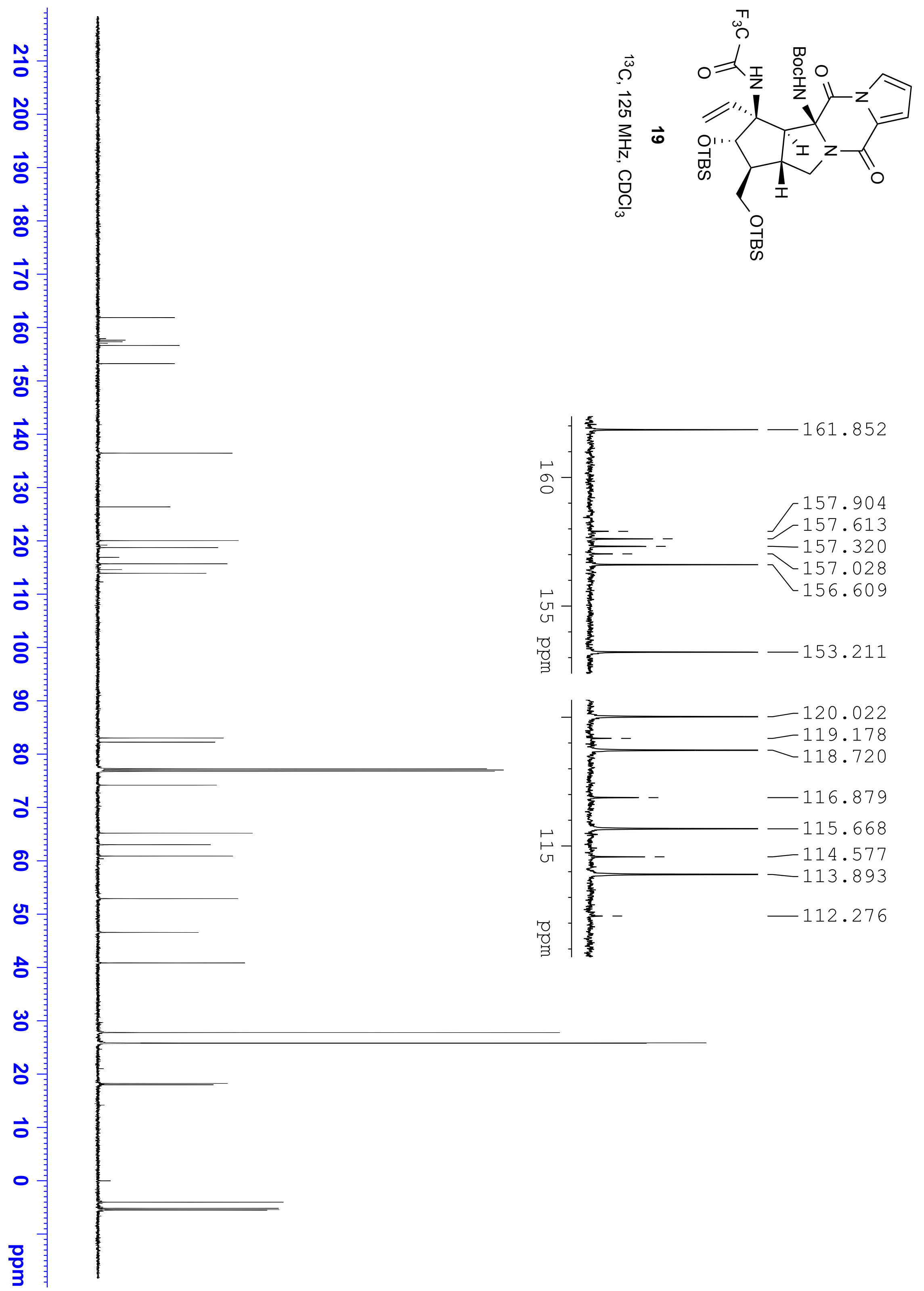

Supplementary Figure 28. ^{13}C-NMR spectrum of **19**

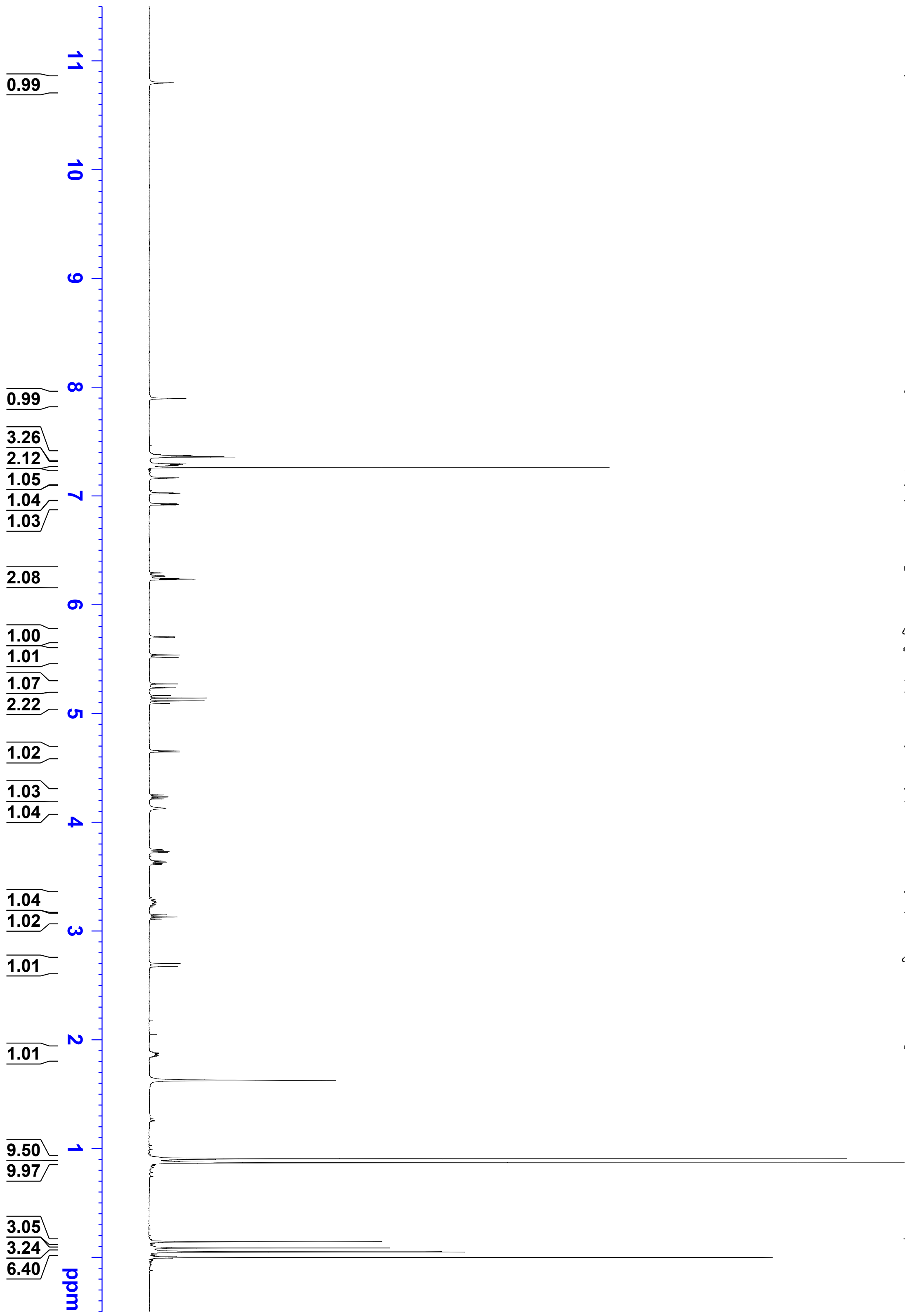

Supplementary Figure 29. ^{1}H-NMR spectrum of **20**

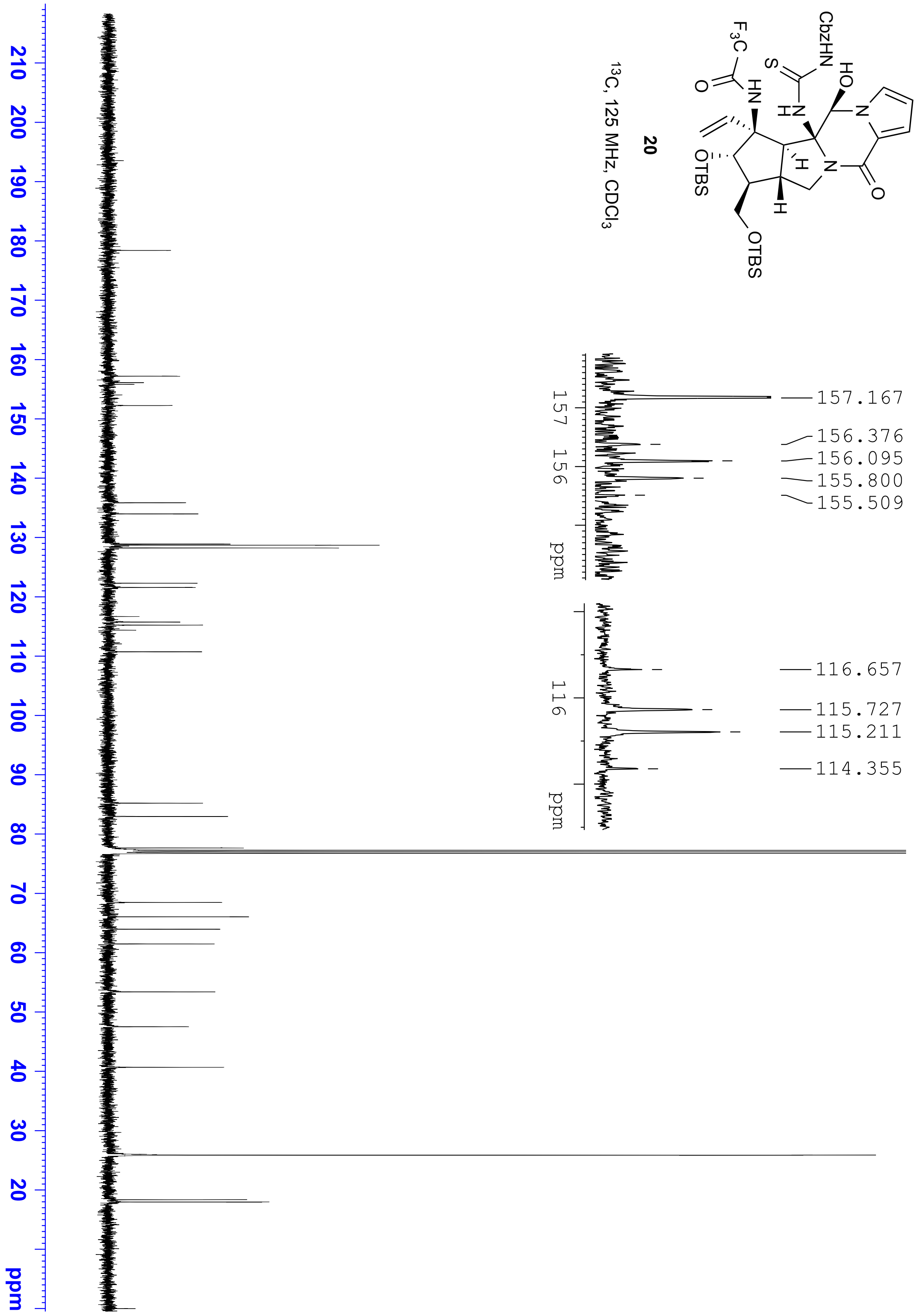

Supplementary Figure 30. ^{13}C-NMR spectrum of **20**

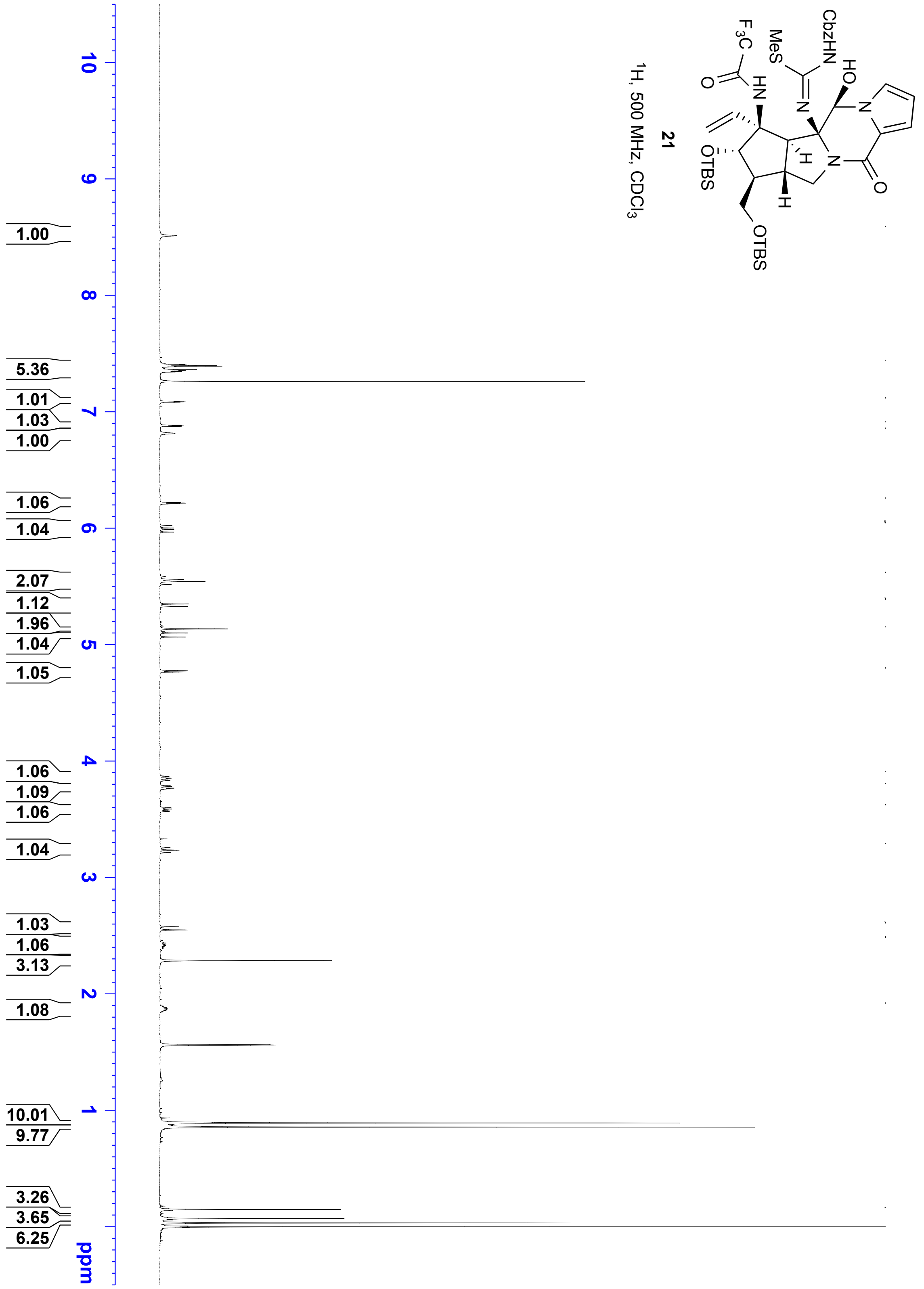

Supplementary Figure 31. ^{1}H-NMR spectrum of **21**

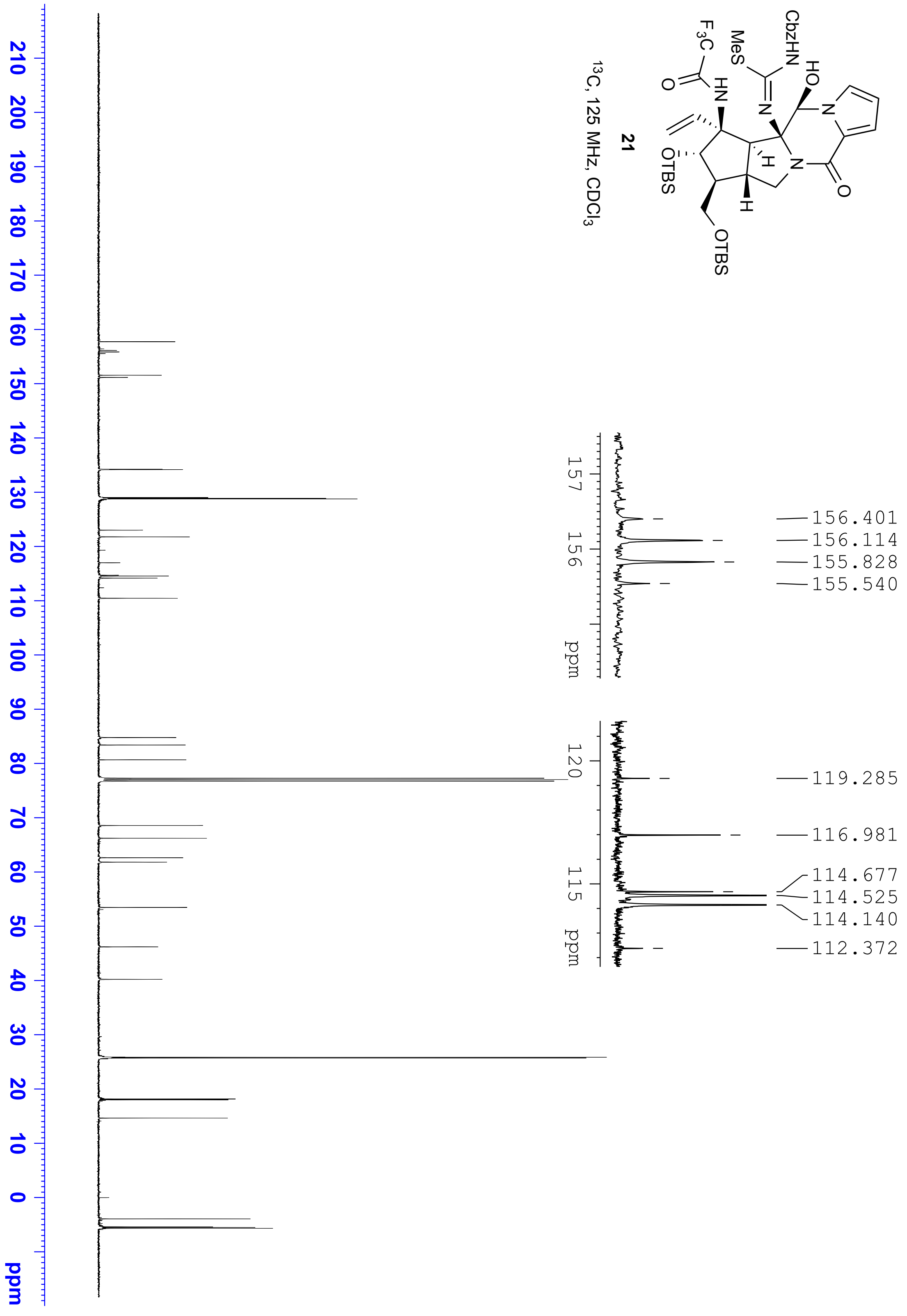

Supplementary Figure 32. ^{13}C-NMR spectrum of **21**

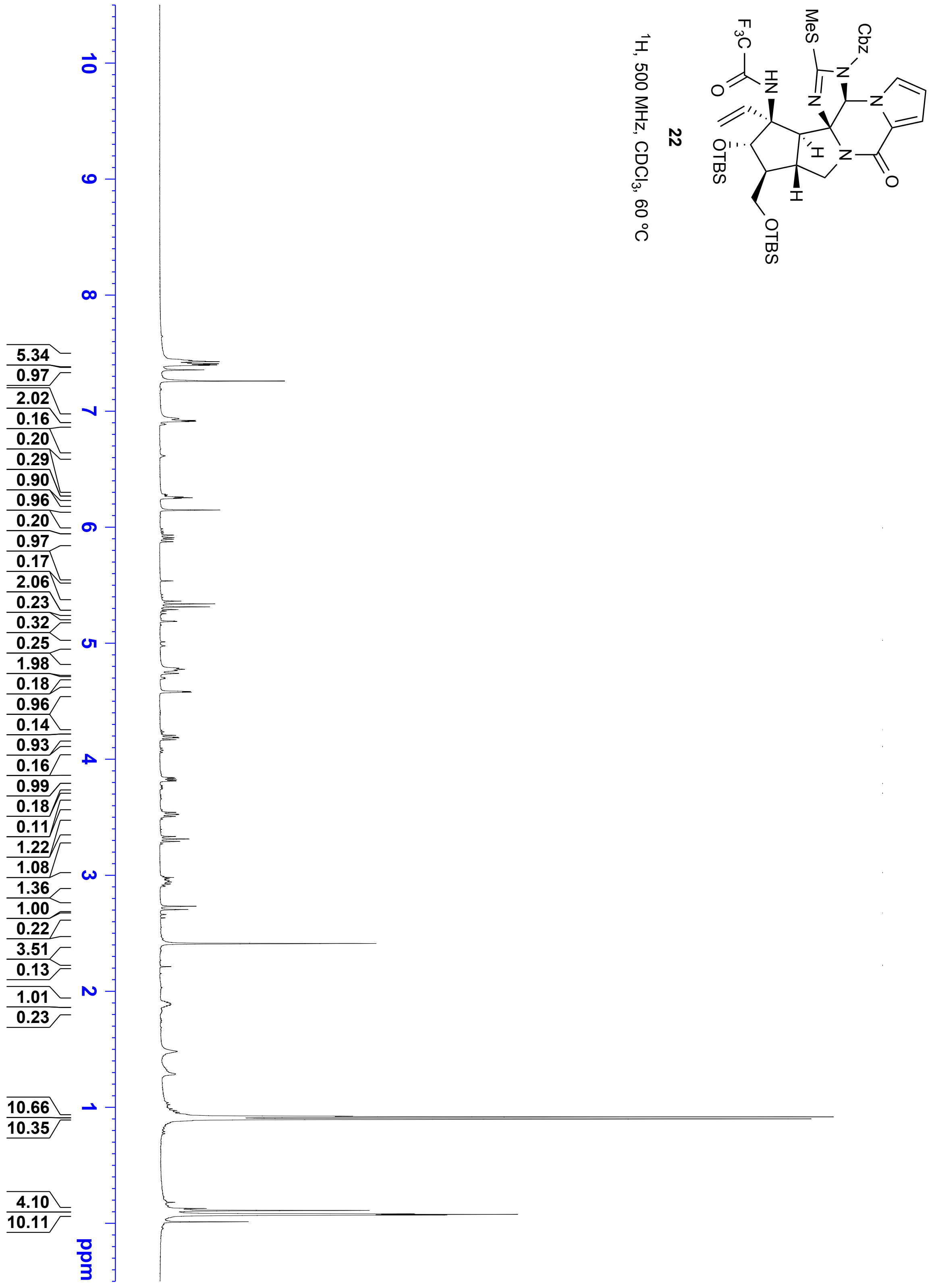

Supplementary Figure 33. ^{1}H-NMR spectrum of **22**

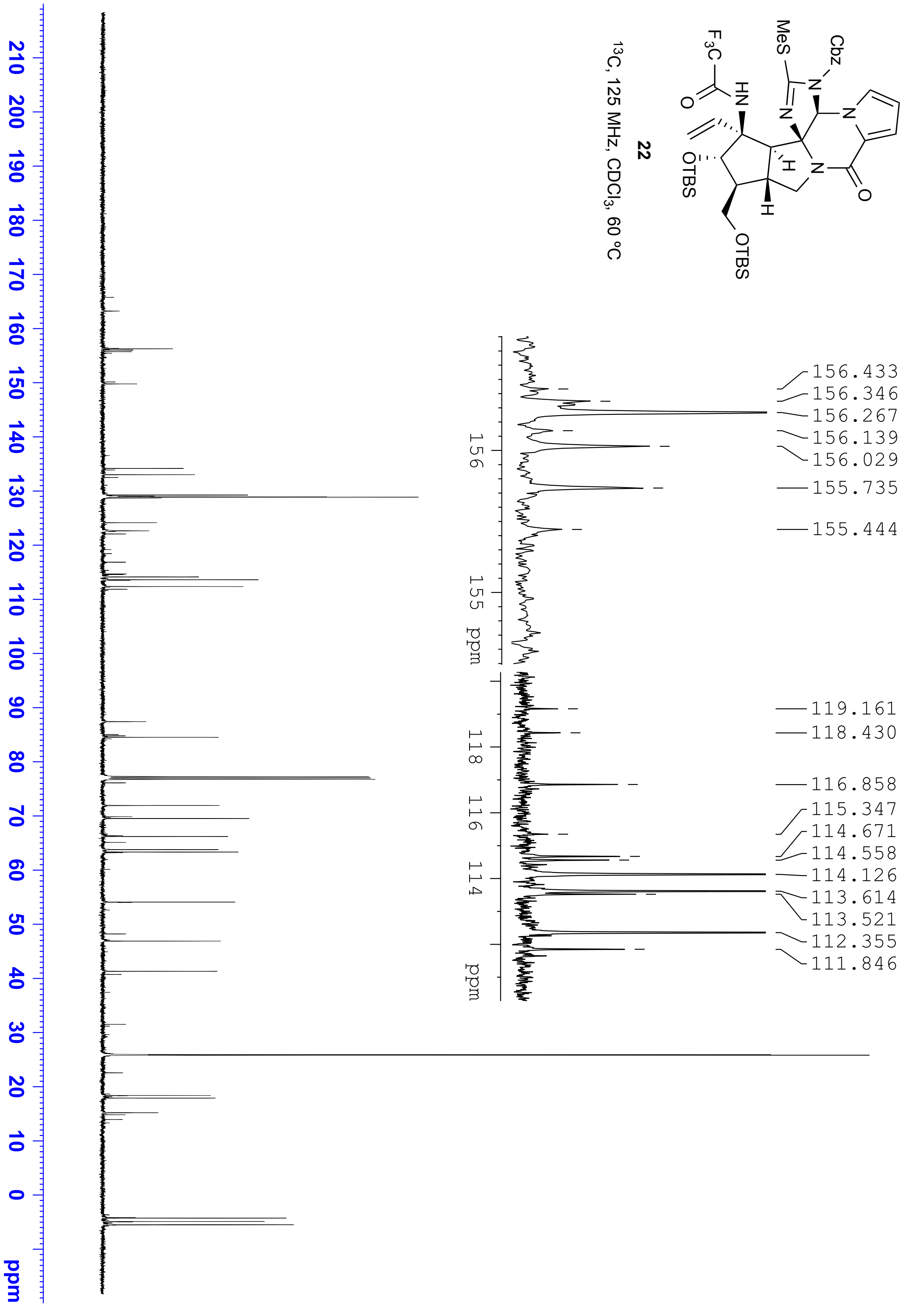

Supplementary Figure 34. ^{13}C-NMR spectrum of **22**

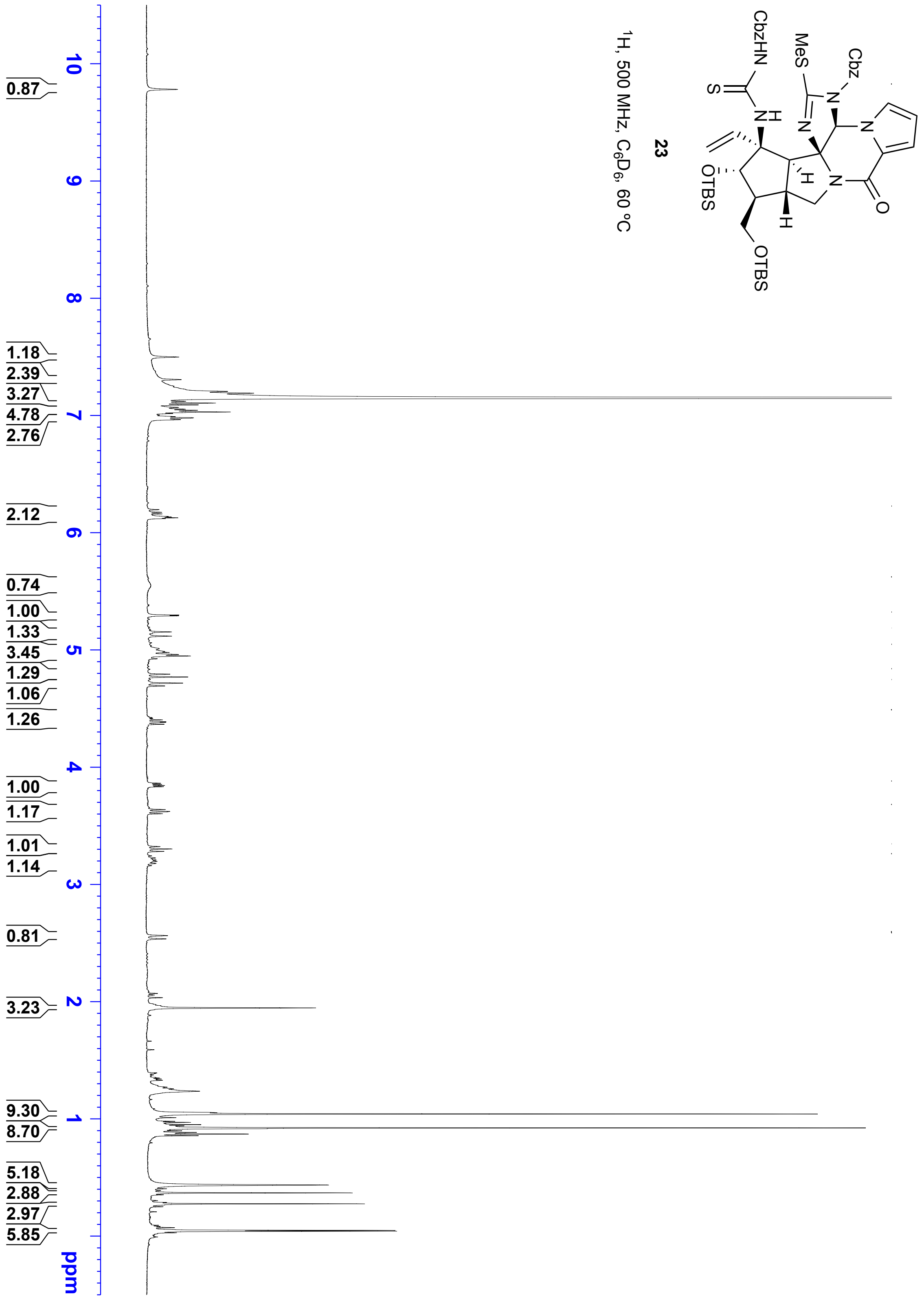

Supplementary Figure 35. ^{1}H-NMR spectrum of **23**

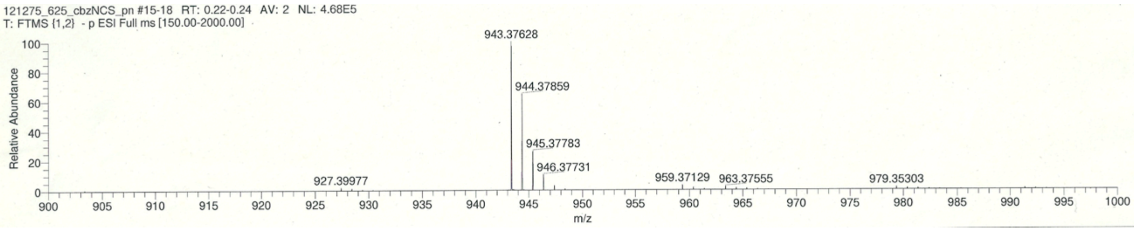

Elemental composition search on mass 943.37628

m/z= 938.37628-948.37628

Isotope	Min	Max
O-16	0	8
C-12	0	50
H-1	0	70
Si-28	0	2
N-14	0	7
S-32	0	2

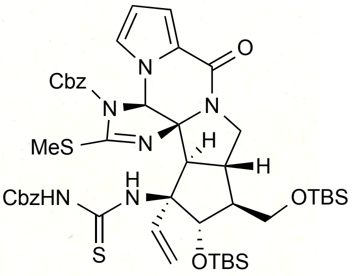

23

Charge -1
Mass tolerance 5.00 ppm
Nitrogen rule not used
RDB equiv -1.00-100.00
max results 100

m/z	Theo. Mass	Delta (ppm)	RDB equiv.	Composition
943.37628	943.37641	-0.13	27.0	$C_{50}H_{57}O_8N_7SSi$
	943.37571	0.61	21.0	$C_{49}H_{65}O_8N_3S_2Si_2$
	943.37705	-0.81	26.0	$C_{50}H_{61}O_4N_7S_2Si_2$
	943.37437	2.03	21.5	$C_{47}H_{63}O_7N_6S_2Si_2$
	943.37978	-3.71	22.0	$C_{47}H_{61}O_8N_7S_2Si$

Supplementary Figure 36. HRMS (ESI) spectrum of **23**

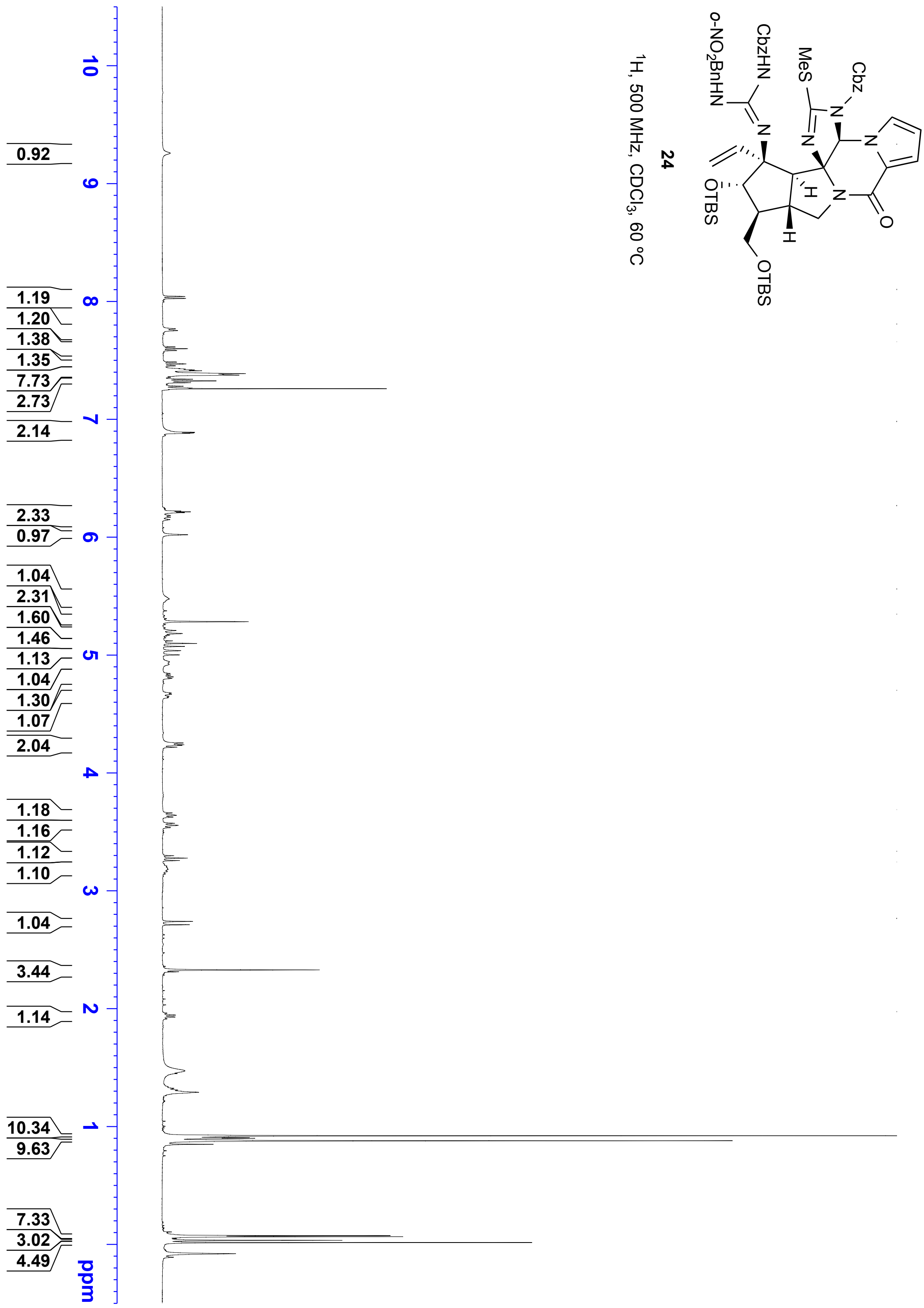

Supplementary Figure 37. ^{1}H-NMR spectrum of **24**

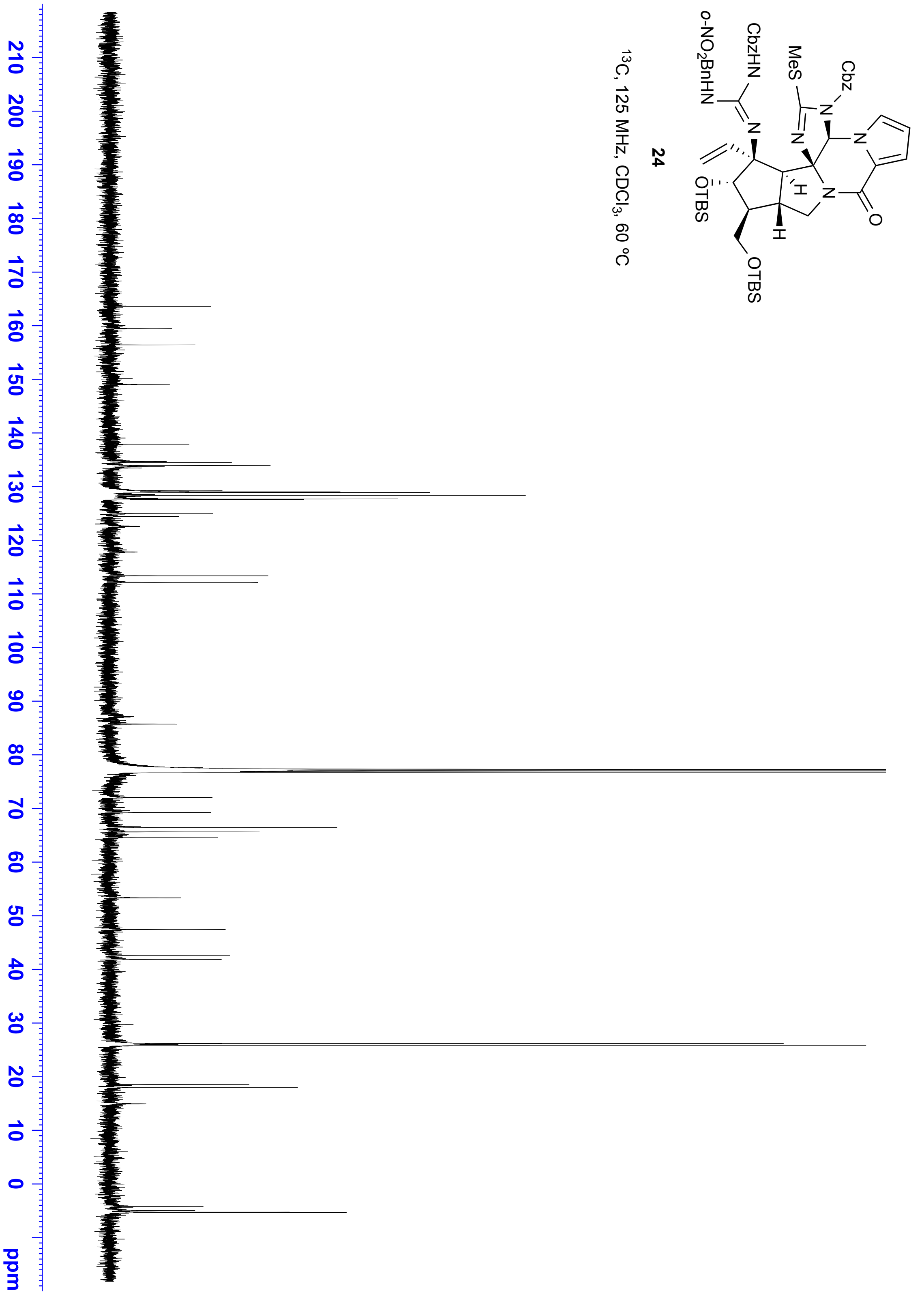

Supplementary Figure 38. ^{13}C-NMR spectrum of **24**

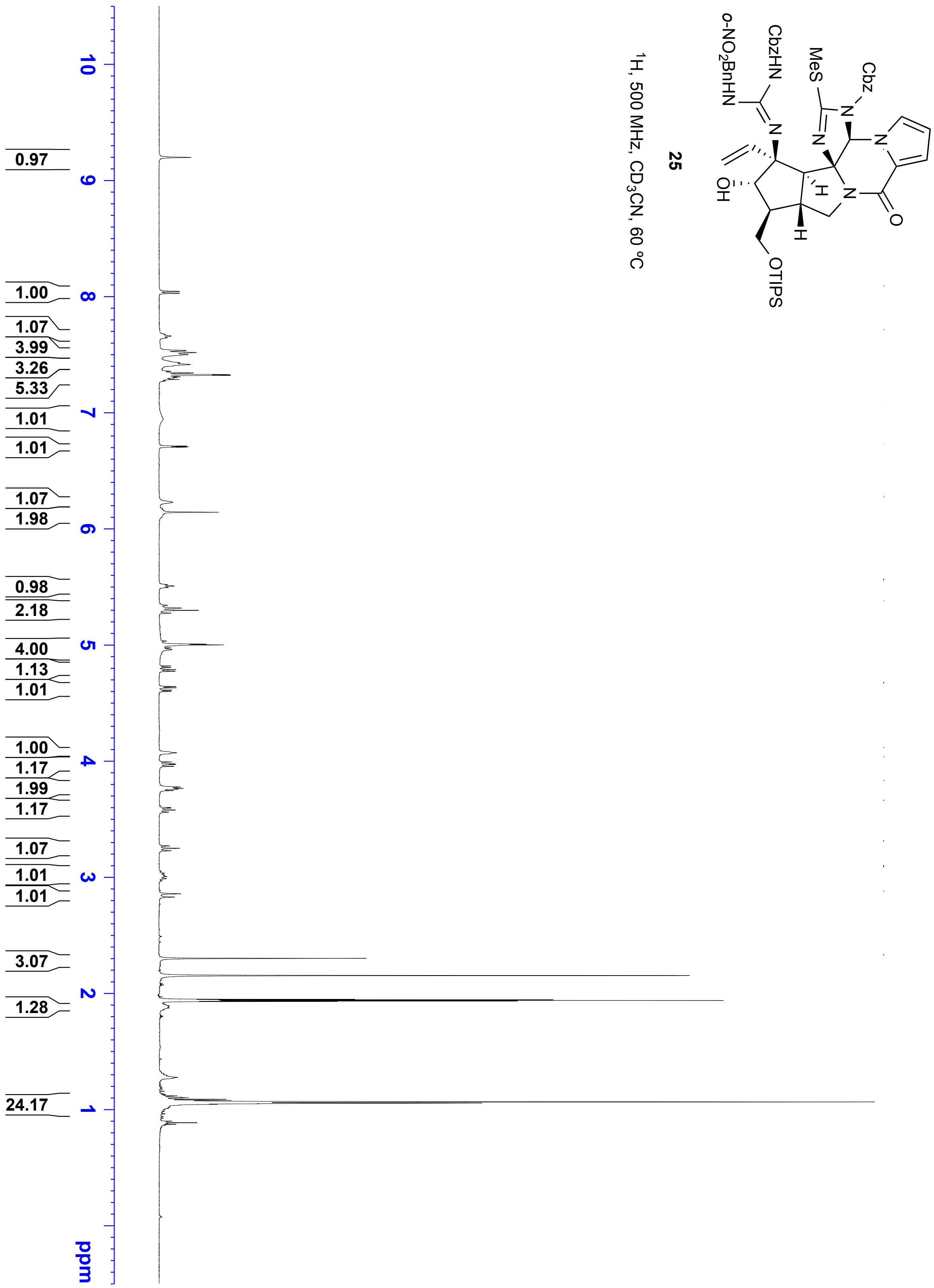

Supplementary Figure 39. ^{1}H-NMR spectrum of **25**

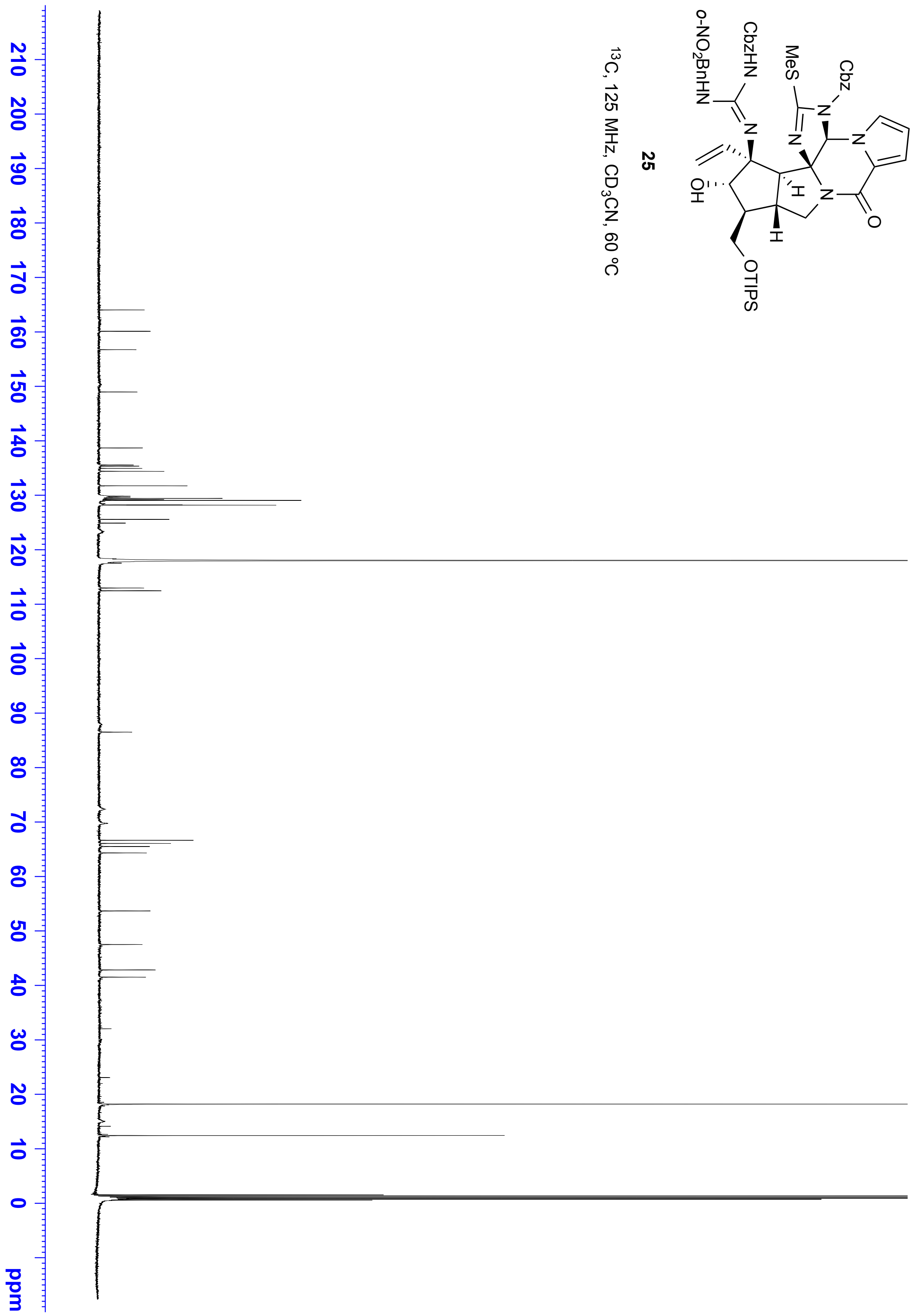

Supplementary Figure 40. ^{13}C-NMR spectrum of **25**

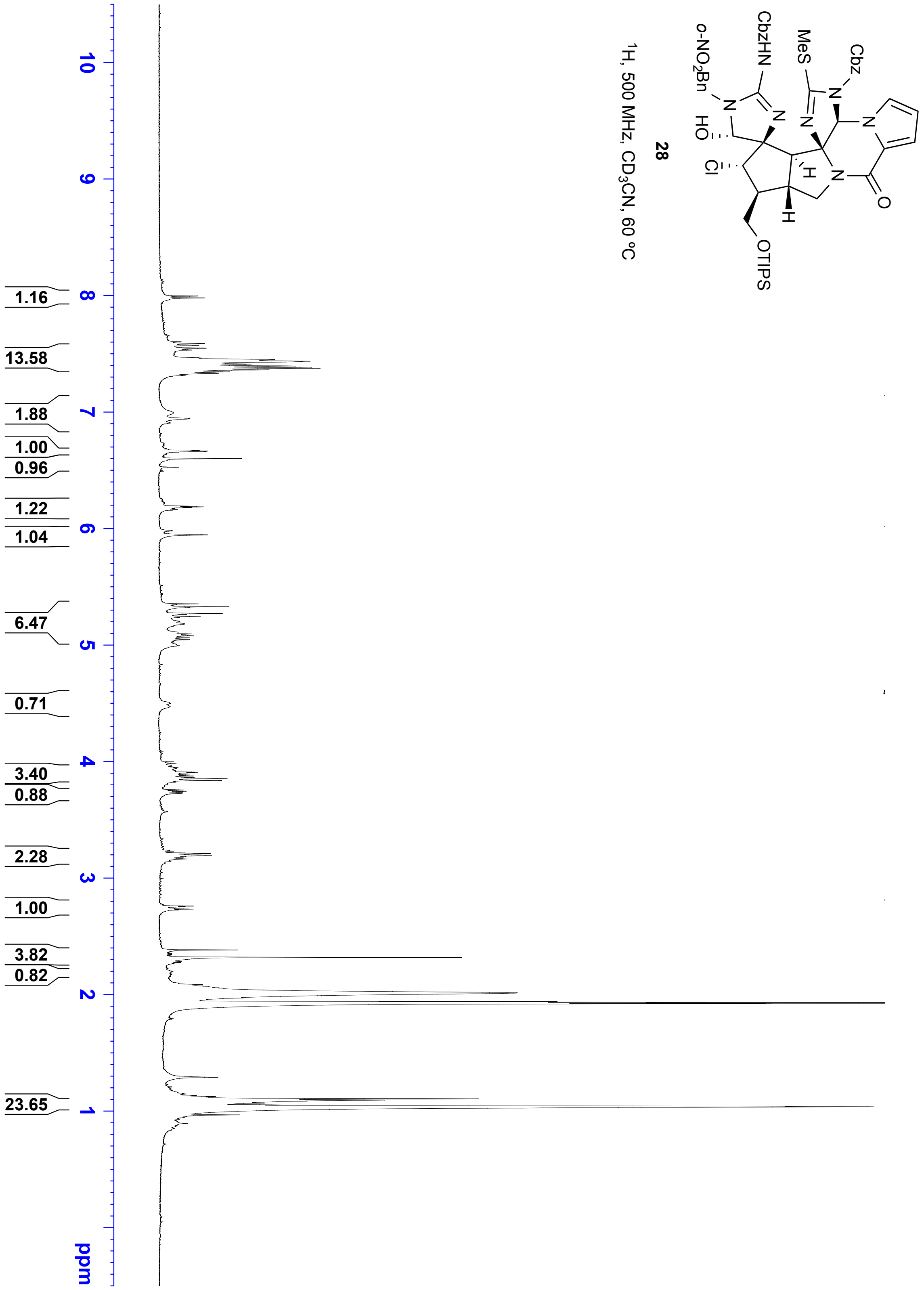

Supplementary Figure 41. ^{1}H-NMR spectrum of **28**

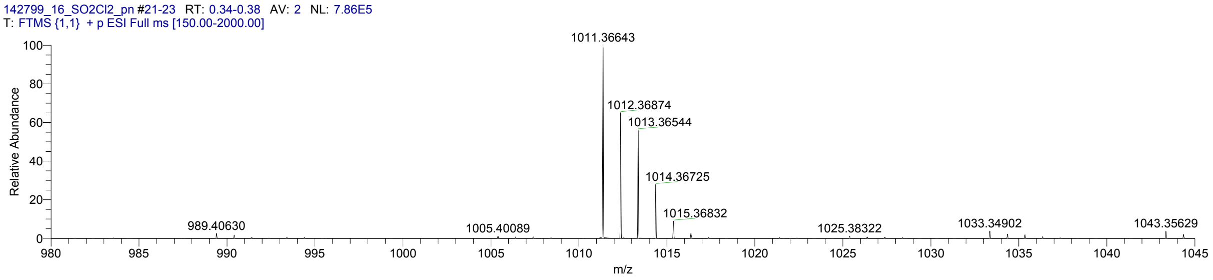

Elemental composition search on mass 1011.36643

m/z= 1006.36643-1016.36643

Isotope	Min	Max
C-12	0	60
H-1	0	120
O-16	0	9
N-14	0	8
Si-28	0	1
S-32	0	1
Cl-35	0	1

Charge 1
Mass tolerance 5.00 ppm
Nitrogen rule not used
RDB equiv -1.00-100.00
max results 100

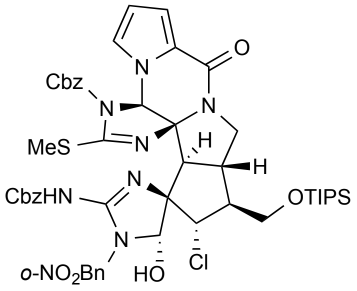

28

m/z	Theo. Mass	Delta (ppm)	RDB equiv.	Composition
1011.36643	1011.36651	-0.08	34.5	$C_{59} H_{56} O_6 N_6 Cl S$
	1011.36628	0.15	34.5	$C_{58} H_{56} O_7 N_6 Cl Si$
	1011.36563	0.79	25.5	$C_{50} H_{60} O_9 N_8 Cl S Si$
	1011.36762	-1.18	34.0	$C_{60} H_{58} O_8 N_3 Cl Si$
	1011.36782	-1.37	34.5	$C_{56} H_{55} O_7 N_8 S Si$
	1011.36468	1.73	39.5	$C_{60} H_{51} O_6 N_8 S$
	1011.36445	1.96	39.5	$C_{59} H_{51} O_7 N_8 Si$
	1011.36383	2.57	30.0	$C_{56} H_{58} O_9 N_5 Cl S$
	1011.36916	-2.70	34.0	$C_{58} H_{57} O_8 N_5 S Si$
	1011.36965	-3.18	29.5	$C_{55} H_{60} O_7 N_6 Cl S Si$
	1011.36249	3.90	30.5	$C_{54} H_{56} O_8 N_8 Cl S$
	1011.37050	-4.03	33.5	$C_{60} H_{59} O_9 N_2 S Si$
	1011.36226	4.13	30.5	$C_{53} H_{56} O_9 N_8 Cl Si$
	1011.36200	4.38	35.0	$C_{57} H_{53} O_9 N_7 S$
	1011.37099	-4.51	29.0	$C_{57} H_{62} O_8 N_3 Cl S Si$

Supplementary Figure 42. HRMS (ESI) spectrum of **28**

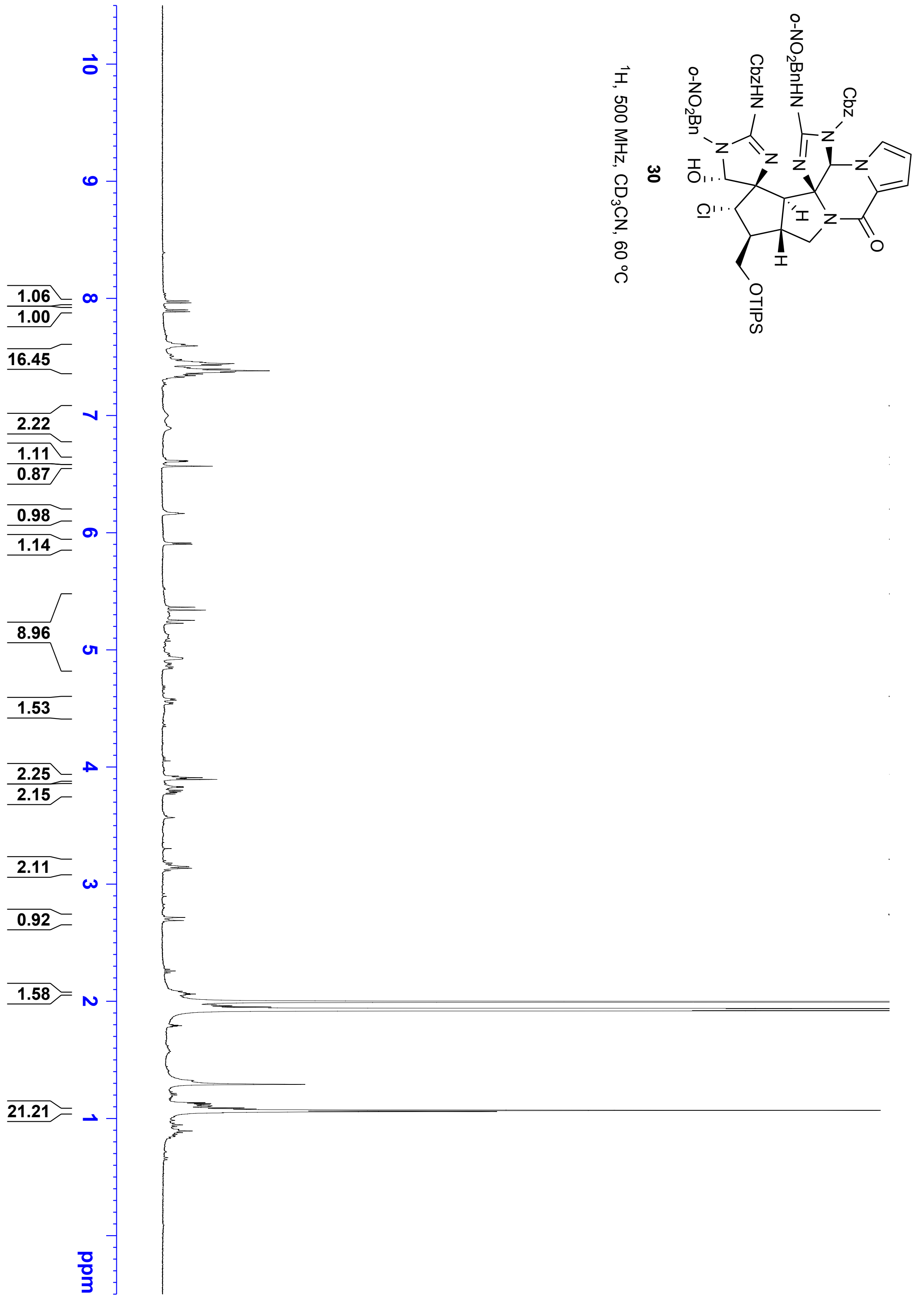

Supplementary Figure 43. ^{1}H-NMR spectrum of **30**

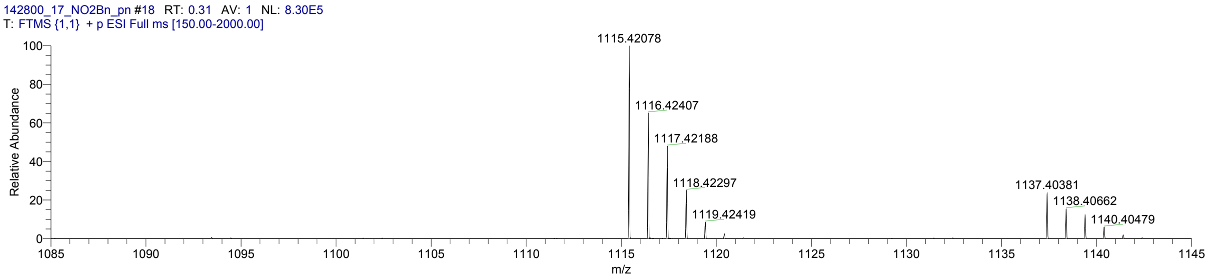

Elemental composition search on mass 1115.42078

m/z= 1110.42078-1120.42078

Isotope	Min	Max
C-12	0	60
H-1	0	120
O-16	0	11
N-14	0	10
Si-28	0	1
Cl-35	0	1

Charge 1
Mass tolerance 5.00 ppm
Nitrogen rule not used
RDB equiv -1.00-100.00
max results 100

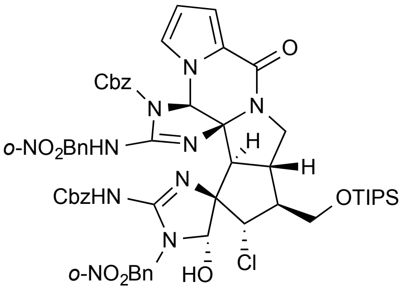

30

m/z	Theo. Mass	Delta (ppm)	RDB equiv.	Composition
1115.42078	1115.42083	-0.05	30.5	$C_{56}H_{64}O_{11}N_{10}ClSi$
	1115.41769	2.77	35.5	$C_{60}H_{60}O_{10}N_{10}Cl$

Supplementary Figure 44. HRMS (ESI) spectrum of **30**

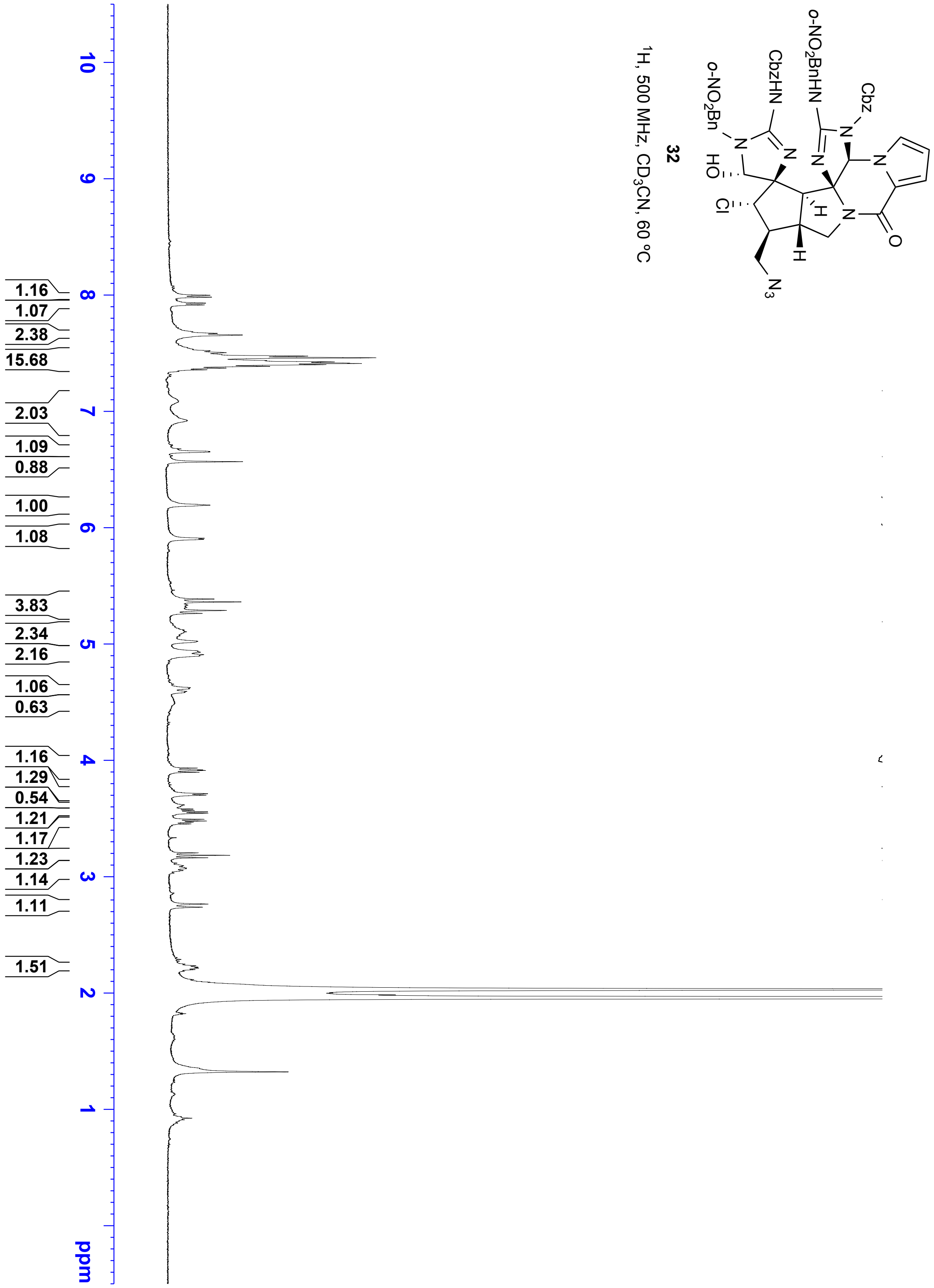

Supplementary Figure 45. ¹H-NMR spectrum of **32**

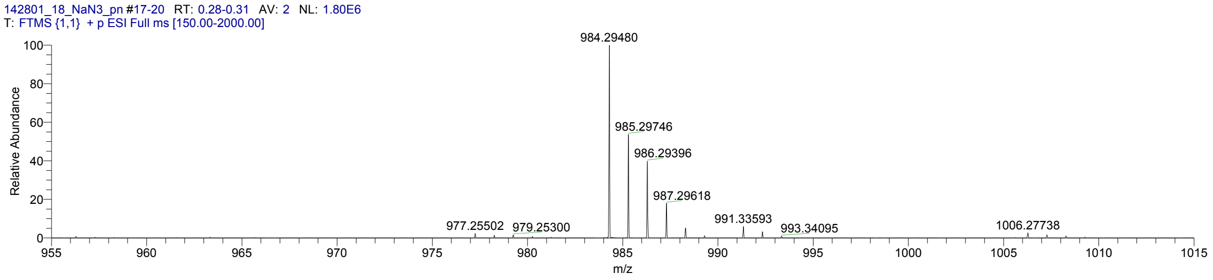

Elemental composition search on mass 984.29480

m/z= 979.29480-989.29480

Isotope	Min	Max
C-12	0	60
H-1	0	120
O-16	0	10
N-14	0	13
Cl-35	0	1

Charge 1
Mass tolerance 5.00 ppm
Nitrogen rule not used
RDB equiv -1.00-100.00
max results 100

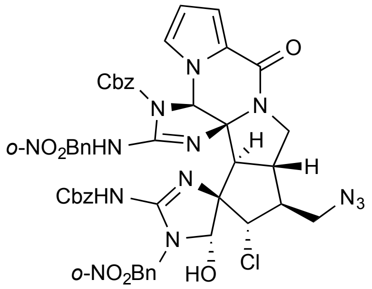

32

m/z	Theo. Mass	Delta (ppm)	RDB equiv.	Composition
984.29480	984.29389	0.93	32.5	C 47 H 43 O 10 N 13 Cl
	984.29608	-1.30	41.5	C 53 H 38 O 8 N 13
	984.29742	-2.67	41.0	C 55 H 40 O 9 N 10
	984.29204	2.80	40.0	C 60 H 45 O 8 N 4 Cl
	984.29204	2.81	45.5	C 59 H 39 O 3 N 11 Cl
	984.29791	-3.16	36.5	C 52 H 43 O 8 N 11 Cl
	984.29877	-4.03	40.5	C 57 H 42 O 10 N 7
	984.29070	4.16	40.5	C 58 H 43 O 7 N 7 Cl
	984.29925	-4.53	36.0	C 54 H 45 O 9 N 8 Cl
	984.29021	4.66	50.5	C 60 H 34 O 3 N 13

Supplementary Figure 46. HRMS (ESI) spectrum of **32**

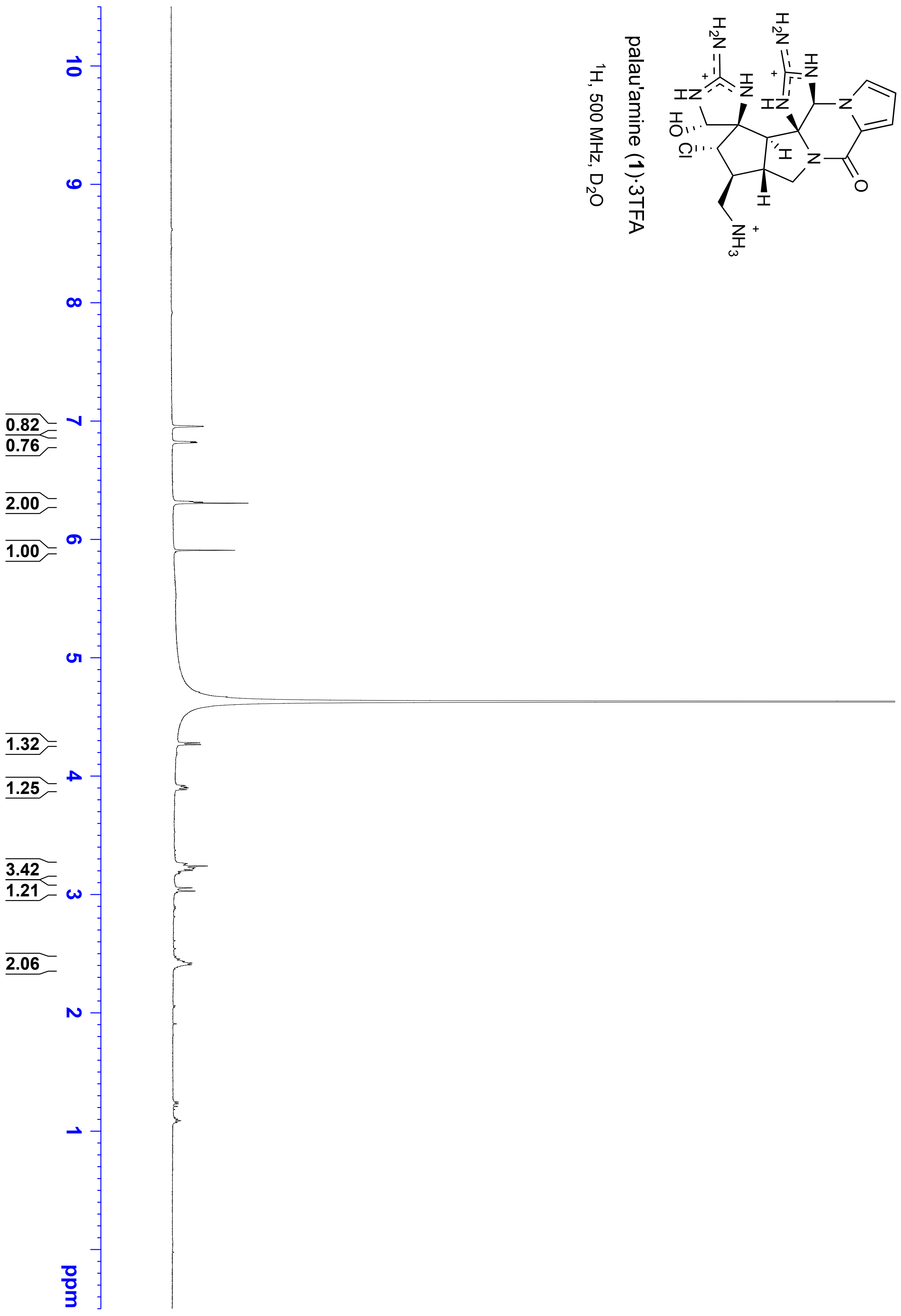

Supplementary Figure 47. ^{1}H-NMR spectrum of palau’amine (**1**)

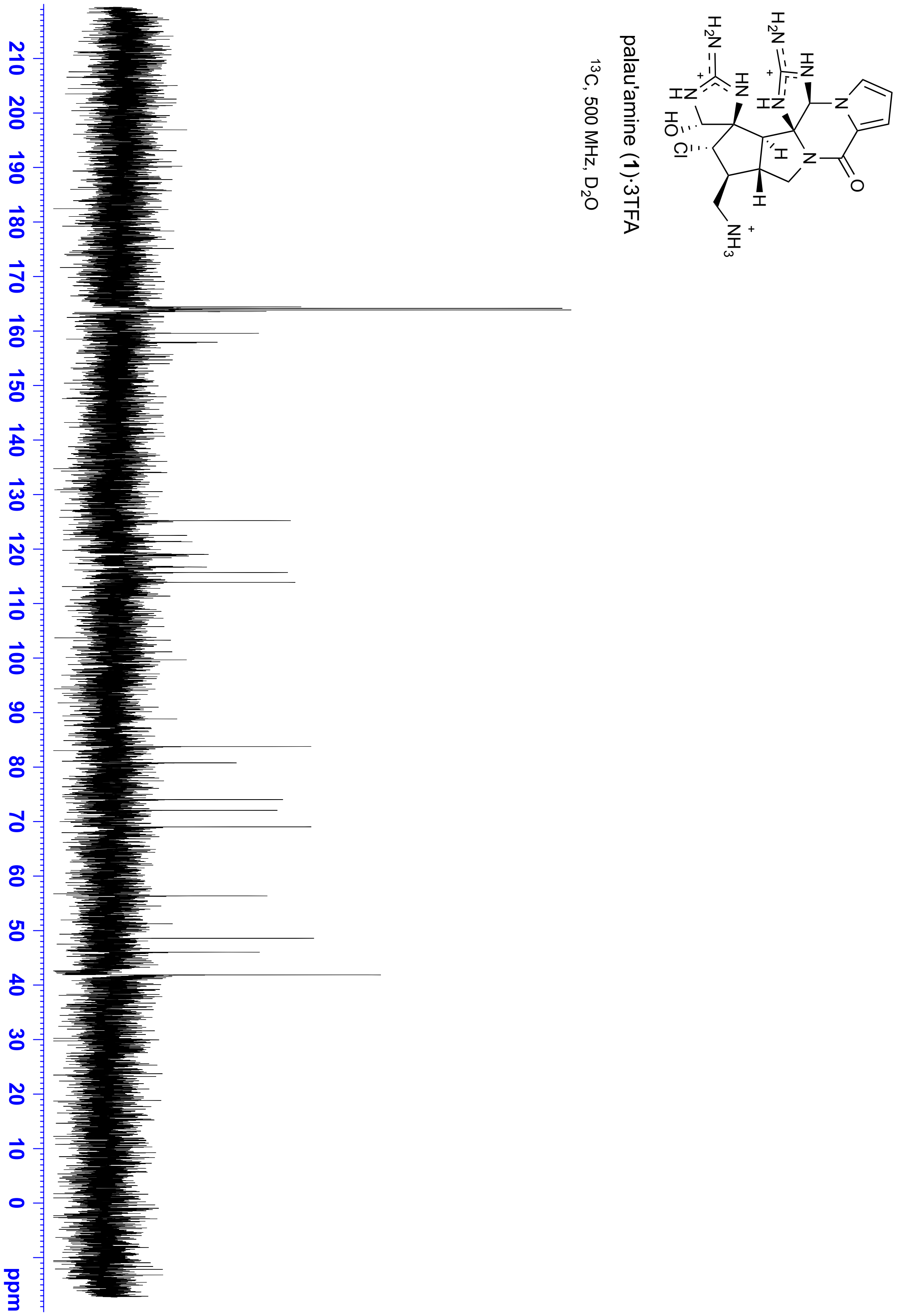

Supplementary Figure 48. ^{13}C-NMR spectrum of palau'amine (**1**)

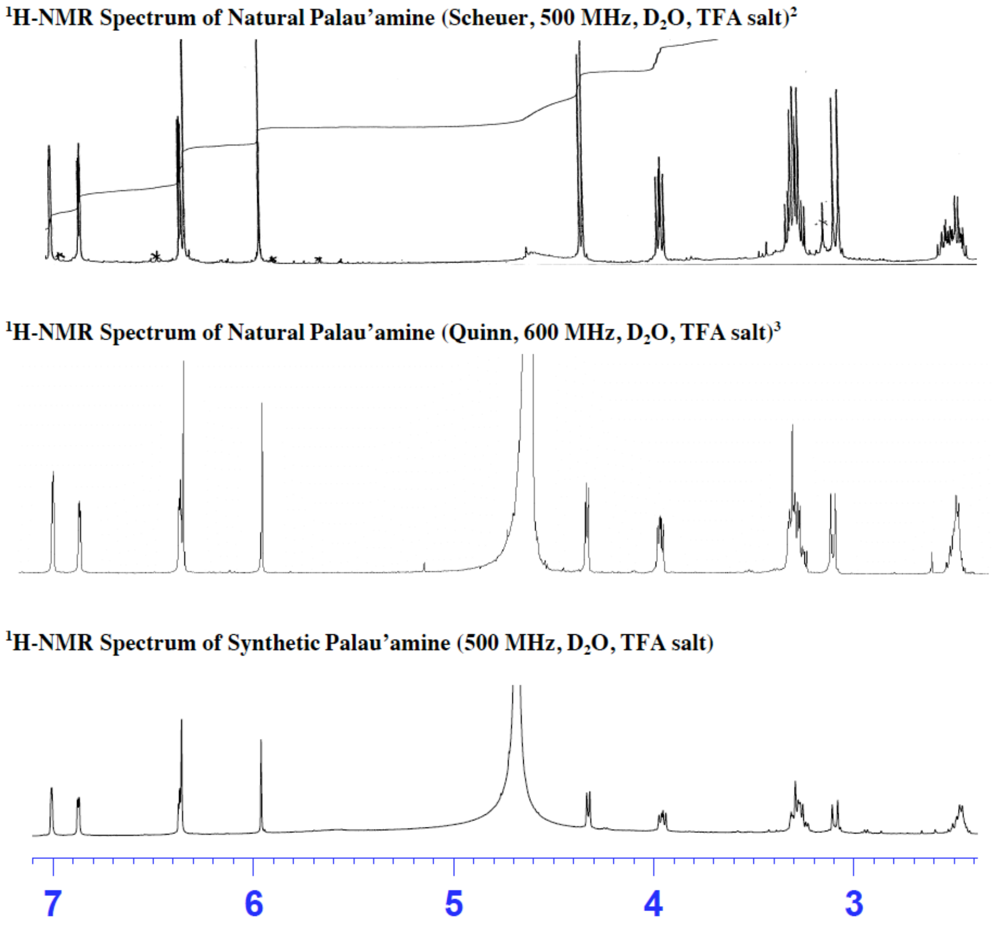

Supplementary Figure 49. Comparison of ^{1}H-NMR Spectra of natural and synthetic Palau'amine (**1**)

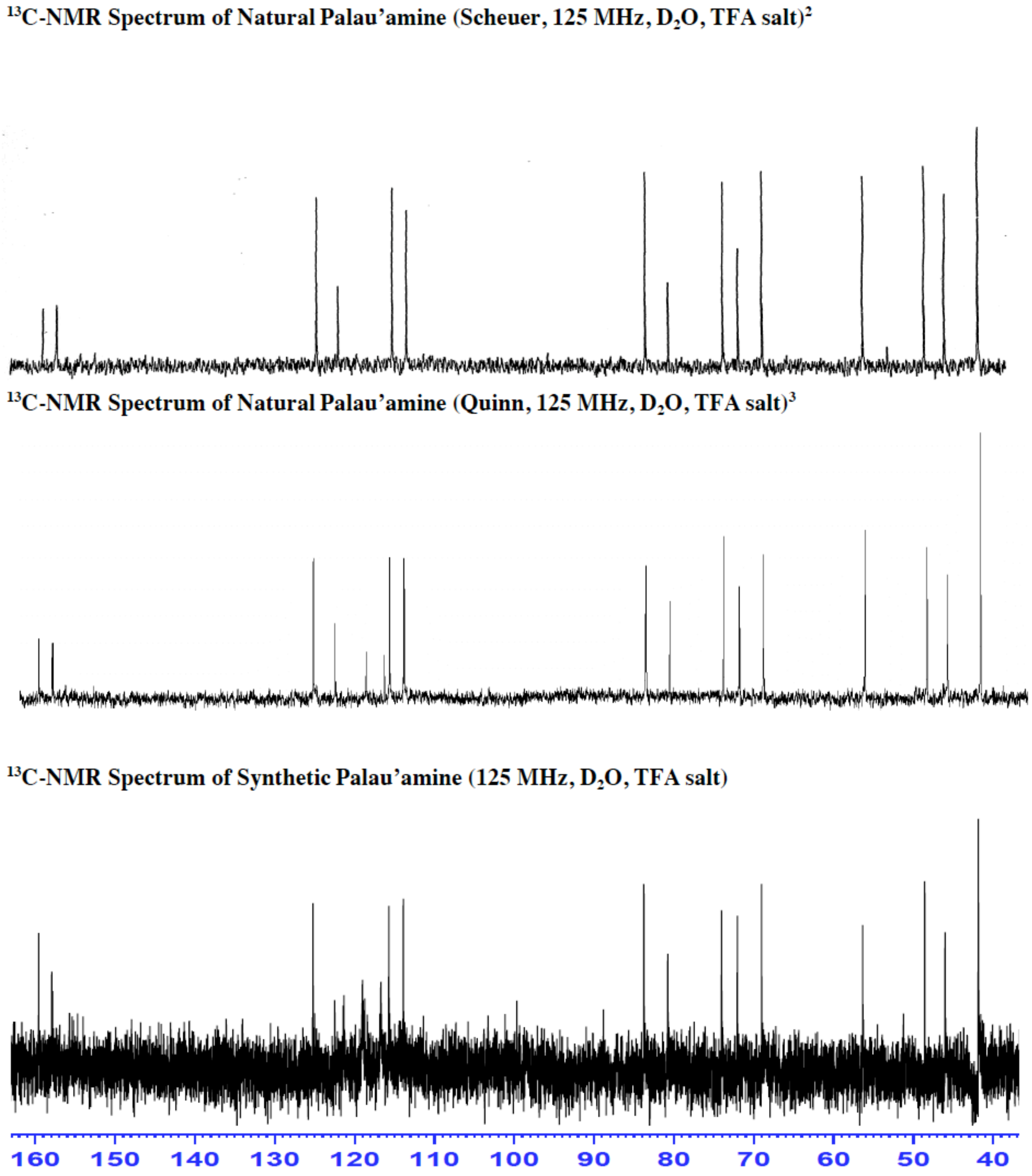

Supplementary Figure 50. Comparison of ^{13}C-NMR Spectra of natural and synthetic Palau'amine (**1**)

Reverse–Phase HPLC

(Atlantis dC18, 5 μm, 250 x 4.6 mm, 100% H_2O (0.1% HCO_2H), 1 mL/min)

Synthetic Palau'amine (**1**)

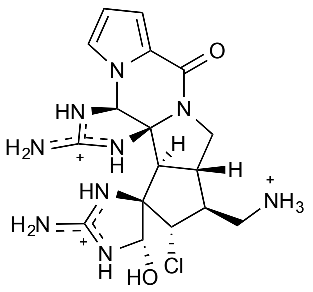

palau'amine (**1**)·3TFA

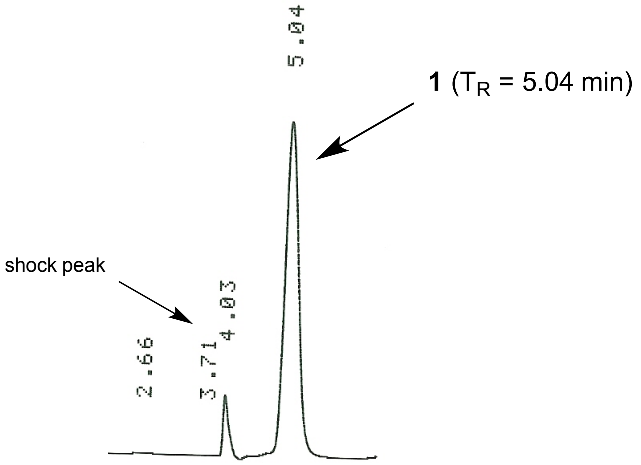

Brank (100% H_2O (0.1% HCO_2H))

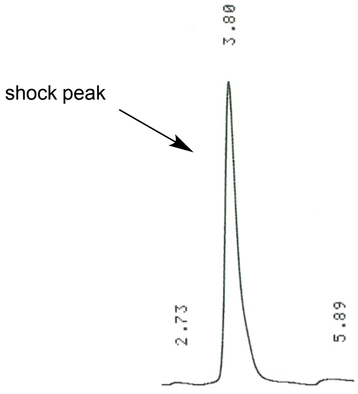

Supplementary Figure 51. HPLC Analysis of Synthetic Palau'amine (1)

Supplementary Tables

Position	Natural (Scheuer)[2-3]	Natural (Quinn)[4]	Synthetic
**3**	6.85 (dd, 3.9, 1.5)	6.89 (dd, 3.9, 1.6)	6.87 (dd, 4.0, 1.5)
**4**	6.35 (dd, 3.9, 2.8)	6.39 (dd, 3.9, 2.8)	6.37 (dd, 4.0, 2.5)
**5**	6.99 (dd, 2.8, 1.5)	7.03 (dd, 2.8, 1.6)	7.01 (dd, 2.5, 1.5)
**6**	6.33 (s)	6.37 (s)	6.36 (s)
**11**	3.08 (d, 14.1)	3.11 (d, 13.8)	3.09 (d, 14.0)
**12**	2.52 (dddd)	2.50 (m)	2.49 (m)
**13**	3.96 (dd, 10.4, 7.3)α	3.97 (dd, 10.2, 7.2)α	3.95 (dd, 10.0, 7.0)α
	3.28 (dd, 10.4, 10.3)β	3.31 (t, 10.2)β	3.29 (t, 10.2)β
**17**	4.35 (d, 7.9)	4.34 (d, 7.8)	4.33 (d, 7.5)
**18**	2.47 (dddd)	2.48 (m)	2.47 (m)
**19**	3.32 (dd, 13.2, 7.0)a	3.32 (dd, 13.2, 6.6)a	3.31 (dd, 13.2, 6.5)a
	3.24 (dd, 13.2, 7.0)b	3.27 (dd, 13.2, 6.6)b	3.26 (dd, 13.2, 6.5)b
**20**	5.96 (s)	5.98 (s)	5.96 (s)

Supplementary Table 1. ^{1}H-NMR Comparison of natural and synthetic Palau'amine (**1**)

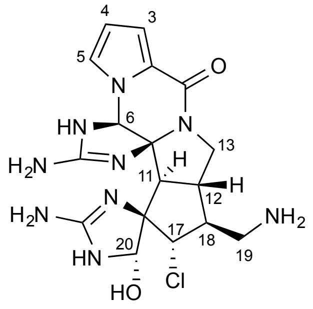

palau'amine (**1**)·3TFA

Position	Natural (Scheuer)[2-3]	Natural (Quinn)[4]	Synthetic
**2**	122.5	122.5	122.5
**3**	115.6	115.7	115.7
**4**	113.8	113.9	113.9
**5**	125.2	125.2	125.2
**6**	69.0	69.0	69.0
**8**	157.8[3, 5-6] (159.6)[2]	157.8	157.9
**10**	80.8	80.7	80.8
**11**	56.3	56.3	56.4
**12**	41.8	41.8	41.9
**13**	46.1	46.0	46.0
**15**	159.5[3, 5-6] (157.8)[2]	159.5	159.6
**16**	72.1	72.0	72.1
**17**	74.0	74.0	74.0
**18**	48.6	48.6	48.6
**19**	41.9	41.8	41.9
**20**	83.7	83.7	83.8
**22**	157.9	157.9	157.8

Supplementary Table 2. ^{13}C-NMR Comparison of natural and synthetic Palau'amine (**1**)

**18A**			
	x	*y*	*z*
C	0.2517	−0.8517	−0.5261
C	0.4778	0.6326	−0.8613
C	1.1659	−1.0999	0.7118
C	1.9689	0.8622	−0.5282
C	2.4667	−0.4152	0.1769
H	0.6704	−1.4404	−1.3470
H	−0.1529	1.2658	−0.2310
H	2.5395	0.9631	−1.4570
H	3.1320	−0.1934	1.0170
C	−1.1634	−1.2803	−0.3230
N	−1.9358	−0.7106	0.5214
C	−1.6217	−2.5390	−1.0555
O	−1.5711	−2.6841	−2.2528
O	−2.0543	−3.4493	−0.2095
C	−2.5409	−4.6763	−0.7842

H	−3.4014	−4.4640	−1.4182
H	−1.7502	−5.1453	−1.3683
H	−2.8222	−5.2980	0.0595
C	−3.2275	−1.2136	0.7487
O	−4.0593	−1.3989	−0.1176
O	−3.4021	−1.3916	2.0319
C	−4.6521	−1.9635	2.5848
C	−5.8289	−1.0549	2.2566
H	−6.6881	−1.3747	2.8493
H	−5.5945	−0.0228	2.5260
H	−6.0980	−1.1004	1.2031
C	−4.8203	−3.3785	2.0497
H	−5.0005	−3.3814	0.9751
H	−3.9291	−3.9699	2.2703
H	−5.6744	−3.8426	2.5464
C	−4.3725	−1.9715	4.0794
H	−5.2200	−2.4149	4.6039
H	−3.4784	−2.5578	4.2971
H	−4.2277	−0.9539	4.4467
C	1.3273	−2.5606	1.0662
H	1.6187	−2.7432	2.0954
C	1.1636	−3.5943	0.2474
H	0.9156	−3.4857	−0.8039
H	1.3044	−4.6087	0.6042
N	0.6575	−0.3335	1.8545
C	1.4294	−0.2951	2.9165
O	2.5200	−0.8155	3.1862
C	0.8919	0.6399	4.0220
F	−0.4117	1.0104	3.8468
F	0.9613	0.0889	5.2308
F	1.5905	1.7832	4.0560
C	0.1268	0.9230	−2.3190
H	0.2166	2.0067	−2.4711
H	0.8837	0.4377	−2.9479
N	−1.1766	0.4300	−2.7172
C	−2.2117	1.1292	−2.2610
O	−2.1888	2.1060	−1.4787
C	−3.5350	0.6618	−2.7960
C	−4.7842	1.2750	−2.6630
N	−3.6379	−0.4787	−3.5522
C	−5.6977	0.4746	−3.3933
C	−4.9451	−0.5785	−3.9256
H	−4.9885	2.1868	−2.1227
H	−6.7559	0.6444	−3.5285
H	−5.2858	−1.3992	−4.5424
Li	−4.7484	−0.7899	−1.7642
Li	−1.7582	−1.1926	−3.6315
Li	−1.1286	0.4786	1.9678
O	3.1002	−1.2594	−0.7655
Si	4.6327	−1.8828	−0.4806
C	4.9931	−2.9874	−1.9695
C	5.8323	−0.4429	−0.3841
H	5.6582	0.1456	0.5216
H	6.8690	−0.7899	−0.3555
H	5.7179	0.2207	−1.2450
C	4.6697	−2.8346	1.1316
H	4.1767	−3.8049	1.0339
H	5.7011	−2.9992	1.4560
H	4.1461	−2.2717	1.9121
C	5.1727	−2.1288	−3.2291
H	6.0307	−1.4562	−3.1403
H	5.3434	−2.7721	−4.1008
H	4.2849	−1.5214	−3.4293
C	6.2787	−3.7854	−1.7081
H	6.5180	−4.4083	−2.5782
H	7.1359	−3.1287	−1.5279

H	6.1731	−4.4475	−0.8435
C	3.8250	−3.9594	−2.1871
H	4.0435	−4.6241	−3.0318
H	3.6462	−4.5840	−1.3061
H	2.9004	−3.4186	−2.4050
C	2.2023	2.1207	0.2924
H	3.2752	2.2515	0.4805
H	1.7019	2.0182	1.2640
O	1.7094	3.2431	−0.4217
Si	0.9008	4.4892	0.3534
C	−0.6308	3.7974	1.1834
H	−1.2212	3.2299	0.4550
H	−0.3579	3.1546	2.0263
H	−1.2542	4.6038	1.5815
C	2.0432	5.2396	1.6393
H	2.3887	4.4741	2.3406
H	2.9235	5.6902	1.1729
H	1.5316	6.0135	2.2177
C	0.4535	5.7346	−0.9949
C	0.1597	7.0990	−0.3528
H	−0.1666	7.8103	−1.1209
H	−0.6380	7.0342	0.3950
H	1.0458	7.5167	0.1339
C	1.6261	5.8789	−1.9751
H	2.5424	6.1978	−1.4678
H	1.8343	4.9341	−2.4821
H	1.3872	6.6327	−2.7351
C	−0.7940	5.2576	−1.7531
H	−0.9974	5.9314	−2.5945
H	−0.6854	4.2415	−2.1412
H	−1.6758	5.2548	−1.1059

Supplementary Table 3. Cartesian Coordinates from DFT calculations (in Å) of **18A**

TS(**18A/18B**)			
	x	***y***	***z***
C	0.4927	−0.9379	−0.3224
C	0.4224	0.4967	−0.8469
C	1.4548	−0.8209	0.8916
C	1.8610	1.0187	−0.7171
C	2.5983	−0.0032	0.1805
H	1.0129	−1.5402	−1.0740
H	−0.2416	1.0714	−0.1966
H	2.3544	1.0092	−1.6941
H	3.2156	0.4836	0.9417
C	−0.8994	−1.4797	−0.1664
N	−1.6977	−0.9177	0.7084
C	−1.0832	−2.9433	−0.5655
O	−1.0604	−3.3976	−1.6868
O	−1.1469	−3.6891	0.5190
C	−1.2008	−5.1090	0.3140
H	−2.0919	−5.3667	−0.2577
H	−0.3094	−5.4385	−0.2198
H	−1.2385	−5.5438	1.3076
C	−2.9543	−1.4324	0.9161
O	−3.5400	−2.2422	0.2083
O	−3.4603	−0.9054	2.0221
C	−4.8145	−1.2113	2.5020
C	−5.8397	−0.7192	1.4882
H	−6.8344	−0.7763	1.9352
H	−5.6378	0.3213	1.2248
H	−5.8362	−1.3203	0.5806
C	−4.9416	−2.7000	2.8017

H	−4.9215	−3.2973	1.8921
H	−4.1305	−3.0198	3.4594
H	−5.8890	−2.8746	3.3155
C	−4.8977	−0.3969	3.7858
H	−5.8768	−0.5356	4.2468
H	−4.1296	−0.7205	4.4908
H	−4.7579	0.6644	3.5722
C	1.9244	−2.1400	1.4567
H	2.3071	−2.0806	2.4708
C	1.9364	−3.3078	0.8247
H	1.6023	−3.4273	−0.2013
H	2.3072	−4.2011	1.3157
N	0.8507	0.0184	1.9324
C	1.6322	0.3961	2.9165
O	2.8140	0.1585	3.2019
C	0.9409	1.3977	3.8690
F	−0.4213	1.4074	3.7647
F	1.2152	1.1585	5.1481
F	1.3323	2.6527	3.6004
C	−0.1989	0.4176	−2.2289
H	−0.5279	1.4096	−2.5484
H	0.5635	0.0625	−2.9329
N	−1.3081	−0.5346	−2.2447
C	−2.5322	0.0139	−2.0220
O	−2.7534	1.1012	−1.4754
C	−3.6720	−0.8025	−2.5305
C	−5.0399	−0.5474	−2.3987
N	−3.4597	−1.9448	−3.2603
C	−5.7001	−1.5847	−3.0986
C	−4.6886	−2.4040	−3.6176
H	−5.4842	0.2839	−1.8723
H	−6.7646	−1.7132	−3.2298
H	−4.7950	−3.2969	−4.2186
Li	−4.4461	−2.5312	−1.4115
Li	−1.4754	−2.2418	−3.2339
Li	−1.0718	0.3322	2.1020
O	3.3765	−0.8621	−0.6310
Si	5.0076	−1.1097	−0.3235
C	5.5608	−2.3596	−1.6266
C	5.8886	0.5356	−0.5257
H	5.5869	1.2321	0.2621
H	6.9739	0.4175	−0.4608
H	5.6521	0.9933	−1.4897
C	5.2691	−1.7416	1.4192
H	4.9498	−2.7815	1.5207
H	6.3249	−1.6726	1.6968
H	4.6855	−1.1426	2.1267
C	5.5437	−1.7094	−3.0171
H	6.2620	−0.8875	−3.0872
H	5.8098	−2.4492	−3.7819
H	4.5528	−1.3149	−3.2615
C	6.9846	−2.8351	−1.3030
H	7.3315	−3.5349	−2.0726
H	7.6948	−2.0026	−1.2707
H	7.0272	−3.3525	−0.3398
C	4.6094	−3.5645	−1.6228
H	4.9518	−4.3140	−2.3468
H	4.5649	−4.0433	−0.6394
H	3.5937	−3.2644	−1.8926
C	1.9179	2.4356	−0.1674
H	2.9562	2.7856	−0.1286
H	1.5247	2.4370	0.8580
O	1.1623	3.2962	−1.0063
Si	0.0869	4.4257	−0.3932
C	−1.1937	3.5635	0.6707
H	−1.7394	2.8048	0.1002

H	−0.7034	3.0915	1.5280
H	−1.9108	4.2874	1.0683
C	1.0272	5.6599	0.6606
H	1.4408	5.1721	1.5482
H	1.8543	6.1076	0.1044
H	0.3708	6.4652	1.0020
C	−0.6980	5.2439	−1.9042
C	−1.9119	6.0766	−1.4653
H	−2.3393	6.5959	−2.3312
H	−2.6968	5.4472	−1.0364
H	−1.6426	6.8371	−0.7249
C	0.3243	6.1600	−2.5909
H	0.6204	6.9904	−1.9432
H	1.2272	5.6105	−2.8734
H	−0.1077	6.5871	−3.5040
C	−1.1562	4.1631	−2.8935
H	−1.6844	4.6286	−3.7350
H	−0.2994	3.6146	−3.2927
H	−1.8318	3.4368	−2.4289

Supplementary Table 4. Cartesian Coordinates from DFT calculations (in Å) of TS (**18A**/**18B**)

**18B**			
	x	*y*	*z*
C	0.4856	−0.8897	−0.3357
C	0.4082	0.5312	−0.8615
C	1.4417	−0.7706	0.8691
C	1.8540	1.0243	−0.8274
C	2.5891	0.0392	0.1248
H	1.0280	−1.4782	−1.0877
H	−0.1962	1.1180	−0.1662
H	2.3198	0.9410	−1.8138
H	3.1869	0.5668	0.8741
C	−0.9976	−1.3412	−0.3971
N	−1.7348	−0.8489	0.7155
C	−1.0120	−2.8713	−0.5162
O	−0.9227	−3.4752	−1.5710
O	−1.0083	−3.4734	0.6497
C	−0.9007	−4.9017	0.6327
H	−1.7636	−5.3327	0.1250
H	0.0154	−5.2017	0.1243
H	−0.8773	−5.2041	1.6751
C	−2.9134	−1.3729	0.9604
O	−3.5369	−2.2393	0.3089
O	−3.4267	−0.8655	2.1162
C	−4.8176	−1.0392	2.4950
C	−5.7400	−0.5033	1.4028
H	−6.7571	−0.4272	1.7937
H	−5.4125	0.4932	1.0962
H	−5.7526	−1.1514	0.5278
C	−5.1083	−2.4975	2.8375
H	−5.0547	−3.1288	1.9529
H	−4.3862	−2.8568	3.5744
H	−6.1085	−2.5746	3.2706
C	−4.9354	−0.1748	3.7458
H	−5.9471	−0.2374	4.1502
H	−4.2320	−0.5174	4.5075
H	−4.7154	0.8680	3.5075
C	1.9374	−2.0722	1.4518
H	2.2666	−2.0055	2.4841
C	2.0444	−3.2299	0.8104
H	1.7616	−3.3522	−0.2309
H	2.4365	−4.1090	1.3109

N	0.8592	0.0920	1.9033
C	1.6519	0.4944	2.8655
O	2.8415	0.2753	3.1399
C	0.9572	1.5046	3.8057
F	−0.4046	1.4650	3.7451
F	1.2798	1.3202	5.0844
F	1.3001	2.7628	3.4840
C	−0.3882	0.3337	−2.1271
H	−0.9071	1.2284	−2.4633
H	0.2495	−0.0357	−2.9374
N	−1.3882	−0.7371	−1.7699
C	−2.7426	−0.2097	−1.8209
O	−3.0124	0.8977	−1.4128
C	−3.7274	−1.0792	−2.4591
C	−5.1207	−0.9303	−2.4558
N	−3.3584	−2.2293	−3.1130
C	−5.6260	−2.0440	−3.1478
C	−4.5073	−2.7969	−3.5451
H	−5.6718	−0.1244	−1.9948
H	−6.6612	−2.2733	−3.3528
H	−4.4930	−3.7150	−4.1163
Li	−4.3450	−2.8298	−1.2198
Li	−1.3846	−2.3983	−3.1107
Li	−1.0734	0.1975	2.1838
O	3.3984	−0.8314	−0.6418
Si	5.0240	−1.0496	−0.2849
C	5.6421	−2.3083	−1.5528
C	5.8825	0.6083	−0.4881
H	5.5475	1.3104	0.2811
H	6.9673	0.5092	−0.3904
H	5.6663	1.0489	−1.4648
C	5.2590	−1.6487	1.4721
H	4.9749	−2.6978	1.5820
H	6.3037	−1.5363	1.7761
H	4.6330	−1.0592	2.1507
C	5.6530	−1.6831	−2.9545
H	6.3439	−0.8370	−3.0132
H	5.9737	−2.4255	−3.6956
H	4.6587	−1.3288	−3.2423
C	7.0668	−2.7414	−1.1772
H	7.4516	−3.4522	−1.9182
H	7.7563	−1.8916	−1.1469
H	7.0936	−3.2327	−0.1999
C	4.7223	−3.5371	−1.5564
H	5.1007	−4.2863	−2.2628
H	4.6667	−4.0052	−0.5685
H	3.7059	−3.2669	−1.8535
C	1.9460	2.4648	−0.3480
H	2.9826	2.8176	−0.3888
H	1.6191	2.5055	0.7005
O	1.1351	3.2916	−1.1688
Si	0.0174	4.3684	−0.5354
C	−1.2383	3.4627	0.5229
H	−1.7983	2.7181	−0.0512
H	−0.7385	2.9591	1.3567
H	−1.9475	4.1762	0.9529
C	0.9116	5.6296	0.5251
H	1.3627	5.1452	1.3962
H	1.7065	6.1299	−0.0331
H	0.2203	6.3923	0.8942
C	−0.7957	5.1532	−2.0487
C	−1.9929	6.0102	−1.6132
H	−2.4449	6.4923	−2.4881
H	−2.7671	5.4057	−1.1318
H	−1.6971	6.8017	−0.9172
C	−1.2817	4.0431	−2.9914

H	−1.7851	4.4826	−3.8612
H	−0.4425	3.4415	−3.3506
H	−1.9933	3.3732	−2.4962
C	0.2230	6.0356	−2.7826
H	0.5434	6.8777	−2.1622
H	1.1123	5.4662	−3.0682
H	−0.2223	6.4461	−3.6969

Supplementary Table 5. Cartesian Coordinates from DFT calculations (in Å) of **18B**

**18B′**			
	x	*y*	*z*
C	0.4856	−0.8897	−0.3357
C	0.4082	0.5312	−0.8615
C	1.4417	−0.7706	0.8691
C	1.8540	1.0243	−0.8274
C	2.5891	0.0392	0.1248
H	1.0280	−1.4782	−1.0877
H	−0.1962	1.1180	−0.1662
H	2.3198	0.9410	−1.8138
H	3.1869	0.5668	0.8741
C	−0.9976	−1.3412	−0.3971
N	−1.7348	−0.8489	0.7155
C	−1.0120	−2.8713	−0.5162
O	−0.9227	−3.4752	−1.5710
O	−1.0083	−3.4734	0.6497
C	−0.9007	−4.9017	0.6327
H	−1.7636	−5.3327	0.1250
H	0.0154	−5.2017	0.1243
H	−0.8773	−5.2041	1.6751
C	−2.9134	−1.3729	0.9604
O	−3.5369	−2.2393	0.3089
O	−3.4267	−0.8655	2.1162
C	−4.8176	−1.0392	2.4950
C	−5.7400	−0.5033	1.4028
H	−6.7571	−0.4272	1.7937
H	−5.4125	0.4932	1.0962
H	−5.7526	−1.1514	0.5278
C	−5.1083	−2.4975	2.8375
H	−5.0547	−3.1288	1.9529
H	−4.3862	−2.8568	3.5744
H	−6.1085	−2.5746	3.2706
C	−4.9354	−0.1748	3.7458
H	−5.9471	−0.2374	4.1502
H	−4.2320	−0.5174	4.5075
H	−4.7154	0.8680	3.5075
C	1.9374	−2.0722	1.4518
H	2.2666	−2.0055	2.4841
C	2.0444	−3.2299	0.8104
H	1.7616	−3.3522	−0.2309
H	2.4365	−4.1090	1.3109
N	0.8592	0.0920	1.9033
C	1.6519	0.4944	2.8655
O	2.8415	0.2753	3.1399
C	0.9572	1.5046	3.8057
F	−0.4046	1.4650	3.7451
F	1.2798	1.3202	5.0844
F	1.3001	2.7628	3.4840
C	−0.3882	0.3337	−2.1271
H	−0.9071	1.2284	−2.4633
H	0.2495	−0.0357	−2.9374
N	−1.3882	−0.7371	−1.7699
C	−2.7426	−0.2097	−1.8209

O	−3.0124	0.8977	−1.4128
C	−3.7274	−1.0792	−2.4591
C	−5.1207	−0.9303	−2.4558
N	−3.3584	−2.2293	−3.1130
C	−5.6260	−2.0440	−3.1478
C	−4.5073	−2.7969	−3.5451
H	−5.6718	−0.1244	−1.9948
H	−6.6612	−2.2733	−3.3528
H	−4.4930	−3.7150	−4.1163
Li	−4.3450	−2.8298	−1.2198
Li	−1.3846	−2.3983	−3.1107
Li	−1.0734	0.1975	2.1838
O	3.3984	−0.8314	−0.6418
Si	5.0240	−1.0496	−0.2849
C	5.6421	−2.3083	−1.5528
C	5.8825	0.6083	−0.4881
H	5.5475	1.3104	0.2811
H	6.9673	0.5092	−0.3904
H	5.6663	1.0489	−1.4648
C	5.2590	−1.6487	1.4721
H	4.9749	−2.6978	1.5820
H	6.3037	−1.5363	1.7761
H	4.6330	−1.0592	2.1507
C	5.6530	−1.6831	−2.9545
H	6.3439	−0.8370	−3.0132
H	5.9737	−2.4255	−3.6956
H	4.6587	−1.3288	−3.2423
C	7.0668	−2.7414	−1.1772
H	7.4516	−3.4522	−1.9182
H	7.7563	−1.8916	−1.1469
H	7.0936	−3.2327	−0.1999
C	4.7223	−3.5371	−1.5564
H	5.1007	−4.2863	−2.2628
H	4.6667	−4.0052	−0.5685
H	3.7059	−3.2669	−1.8535
C	1.9460	2.4648	−0.3480
H	2.9826	2.8176	−0.3888
H	1.6191	2.5055	0.7005
O	1.1351	3.2916	−1.1688
Si	0.0174	4.3684	−0.5354
C	−1.2383	3.4627	0.5229
H	−1.7983	2.7181	−0.0512
H	−0.7385	2.9591	1.3567
H	−1.9475	4.1762	0.9529
C	0.9116	5.6296	0.5251
H	1.3627	5.1452	1.3962
H	1.7065	6.1299	−0.0331
H	0.2203	6.3923	0.8942
C	−0.7957	5.1532	−2.0487
C	−1.9929	6.0102	−1.6132
H	−2.4449	6.4923	−2.4881
H	−2.7671	5.4057	−1.1318
H	−1.6971	6.8017	−0.9172
C	−1.2817	4.0431	−2.9914
H	−1.7851	4.4826	−3.8612
H	−0.4425	3.4415	−3.3506
H	−1.9933	3.3732	−2.4962
C	0.2230	6.0356	−2.7826
H	0.5434	6.8777	−2.1622
H	1.1123	5.4662	−3.0682
H	−0.2223	6.4461	−3.6969

Supplementary Table 6. Cartesian Coordinates from DFT calculations (in Å) of **18B′**

	18A	18A+2THF(I)	18A+2THF(II)
d [C10−N14] / Å	2.94	2.84	3.21
d [Li1−O(MeO_2C−)] / Å	2.04	1.99	5.58
d [Li1−O(THF1)] / Å		1.94	1.91
d [Li2−O(Boc)] / Å	1.89	1.90	1.98
d [Li2−O(THF2)] / Å		1.95	1.89

Supplementary Table 7. Geometrical parameters of **18A**, **18A+2THF(I)** and **18A+2THF(II)**.

	**Compound 21**
Formula	$C_{44}H_{70}F_3N_5O_9SSi_2$
Formula Weight	958.29
Crystal system	monoclinic
Space group	$P2_1/n$ (#14)
Lattice Type	Primitive
a, Å	13.0627(8)
b, Å	22.718(2)
c, Å	18.8564(9)
β	107.908(2)
V, $Å^3$	5324.6(5)
Z	4
d_{calc}, g cm^{-3}	1.195
μ(Mo Kα), cm^{-1}	1.686
Number of Observations	11799
Variables	647
T	173K
R_{int}	0.1118
R1	0.0943
wR2	0.2443
Goodness of Fit Indicator	1.091

Supplementary Table 8. Crystal Data of compound **21**

Supplementary Discussion

Investigation of the solvent effects on chelate formation

The effects of solvent molecules (THF) on the chelate formation are examined by adding explicitly two THF molecules to **18A** in the DFT calculations. This system is immersed in the self-consistent reaction field (continuous) model. We placed two THF molecules around the two lithium ions that are close to the amide and pyrrole anions and optimized the whole structure without any geometrical constraints. The optimized two lowest energy structures, which are denoted as **18A+2THF(I)** and **18A+2THF(II)**, are given in Supplementary Figure 50 and the geometrical parameters are shown in Supplementary Table 7. **18A+2THF(II)** is slightly more stable than **18A+2THF(I)** by 2.3 kcal/mol.

The structure of **18A** moiety in **18A+2THF(I)** is essentially similar to **18A** without any explicit solvent molecules, where one of the lithium ions (Li1) forms a coordination to the carbonyl group of methyl ester and the other (Li2) to the carbonyl group of the Boc group. The oxygen atoms of the two THF molecules, O(THF1) and O(THF2), are coordinates to Li1 and Li2, respectively, and the distance between the oxygen atoms of THF and lithium ions are around 1.94 Å. On the other hand, the structure of **18A+2THF(II)** does not exhibit a coordination of the lithium ion to the carbonyl group of methyl ester, and only the coordination to the carbonyl group of the Boc group is seen. As a result, the distance between C10 and N14 (3.21 Å) is longer than that of **18A+2THF(I)** (2.84 Å).

From these two structures, the potential energy profiles to **18B+2THF(I)** or **18B+2THF(II)** are investigated, and the results are shown in Supplementary Figure 51. As seen in the figure, the energy barrier from **18A+2THF(I)** to **18B+2THF(I)** is only 1.7 kcal/mol and it is quite close to those of **18A** → **18B** (1.5 kcal/mol). On the other hand, the energy barrier from **18A+2THF(II)** to **18B+2THF(II)** is appreciably higher. Considering that the energy difference between **18A+2THF(I)** and **18A+2THF(II)** is only 2.3 kcal/mol, the coordination of lithium ions to both the carbonyl group of methyl ester and the Boc group is a key intermediate step to the facile formation of the trans-bicyclo[3.3.0]octane skeleton.

Supplementary Method

General Procedures.

All the reaction were carried out in a round-bottomed flask with an appropriate number of necks and side arms connected to a three-way stopcock and /or a rubber septum cap under an argon atmosphere. All vessels were first evacuated by rotary pump and then flushed with argon prior to use. Solution and solvent were introduced by hypodermic syringe through a rubber septum. During the reaction, the vessel was kept under a positive pressure of argon. Dry THF was freshly prepared by distillation from benzophenone ketyl before use. Anhydrous CH_2Cl_2, DMF, ethanol, MeCN, methanol, pyridine and toluene were purchased from Kanto Chemical Co. Inc.

Infrared (IR) spectra were recorded on JASCO FT/IR-4100 spectrophotometer using 5 mm KBr plate. Wavelengths of maximum absorbance are quoted in cm^{-1}. ^{1}H-NMR spectra were recorded on a JEOL ECA–400 (400 MHz), JEOL ECA-500 (500 MHz), and Bruker AV–500 (500 MHz) in $CDCl_3$, *d*–MeCN and D_2O. Chemical shifts are reported in part per million (ppm), and signal are expressed as singlet (s), doublet (d), triplet (t), quartet (q), multiplet (m) and broad (br). ^{13}C-NMR spectra were recorded on a JEOL ECA–400 (100 MHz), Bruker AV–400N (100 MHz) and Bruker AV–500 (125 MHz) in $CDCl_3$, C_6D_6, CD_3CN and D_2O. Chemical shifts are reported in part per million (ppm). High resolution mass (HRMS) spectra were recorded on a Thermo Scientific Exactive, Instrumental Analysis Division, Equipment Manager Center Creative Research Institution, Hokkaido University and a Waters SYNAPT-G2 Si HDMS, Tokushima Bunri University. High performance liquid chromatography (HPLC) was recorded on a HITACHI D-2500 Chromato-Integrater. Analytical thin layer chromatography (TLC) was performed using 0.25 mm E. Merck Silica gel (60F-254) plates. Reaction components were visualized phosphomolybdic acid or ninhydrin or *p*-anisaldehyde in 10% sulfuric acid in ethanol. Kanto Chem. Co. Silica Gel 60N (particle size 0.040–0.050 mm) was used for column chromatography.

General procedure for preparation of intermediates and 1

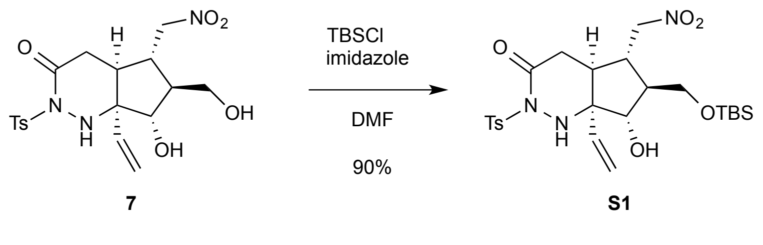

Compound **S1**:

To a solution of alcohol **7** (8.42 g, 19.8 mmol) in DMF (50 mL) were added imidazole (4.04 g, 59.7 mmol) and TBSCl (3.57 g, 23.7 mmol) at 0 ºC. After being stirred at 0 ºC for 1 h, a saturated aqueous NH_4Cl solution (200 mL) was added. The mixture was extracted with EtOAc (200 mL x3). The combined organic layers were dried over anhydrous $MgSO_4$, filtered, and concentrated under reduced pressure. The residue was purified by silica gel flash column chromatography (hexane/EtOAc = 10/1 to 1/1) to afford silyl ether **S1** (9.62 g, 17.8 mmol, 90%) as a white amorphous material. ^1H NMR (400 MHz, $CDCl_3$): δ 7.95 (d, *J* = 8.4 Hz, 2H), 7.34 (d, *J* = 8.0 Hz, 2H), 6.00 (dd, *J* = 17.6, 10.8 Hz, 1H), 5.43 (d, *J* = 10.8 Hz, 1H), 5.34 (d, *J* = 17.6 Hz, 1H), 4.68 (dd, *J* = 12.4, 4.0 Hz, 1H), 4.56 (dd, *J* = 12.0, 7.6 Hz, 1H), 4.40 (s, 1H), 3.93 (dd, *J* = 10.9, 5.2 Hz, 1H), 3.79 (dd, *J* = 10.4, 4.0 Hz, 1H), 3.68 (dd, *J* = 10.8, 5.6 Hz, 1H), 2.70–2.60 (m, 1H), 2.57 (dd, *J* = 15.8, 7.2 Hz, 1H), 2.44 (s, 3H), 2.41 (dt, *J* = 15.6, 4.0 Hz, 1H), 2.22–2.12 (m, 1H), 1.74 (tt, *J* = 10.8, 4.4 Hz, 1H), 0.90 (s, 9H), 0.08 (s, 3H), 0.07 (s, 3H); ^{13}C NMR (125 MHz, $CDCl_3$): δ 170.26, 145.43, 136.24, 134.81, 129.54, 128.84, 118.52, 77.09, 75.16, 69.25, 62.18, 46.39, 42.65, 40.99, 36.74, 25.85, 21.66, 18.18, –5.63, –5.70; IR (KBr): 3479, 3288, 2953, 2928, 2857, 1716, 1597, 1551, 1471, 1433, 1363, 1257, 1171, 1088, 1006, 911, 837, 814 cm^{-1} ; HRMS (ESI, *m/z*): $[M+Na]^+$ calcd for $C_{24}H_{37}O_7N_3NaSSi$, 562.2014; found, 562.2018.

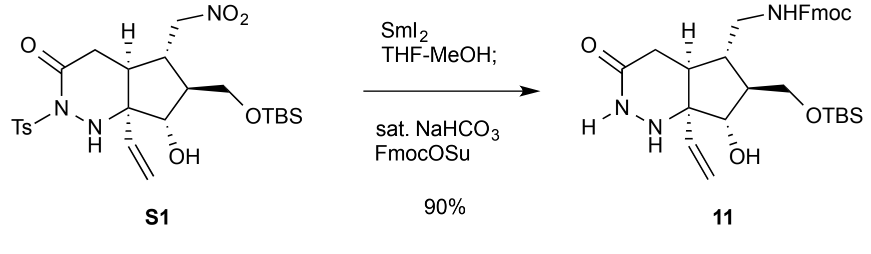

Compound **11**:

To a solution of SmI_2 in THF (0.1 M, 178 mmol, 1.78 L) were added MeOH (89 mL) and a solution of alcohol **S1** (9.62 g, 17.8 mmol) in THF (89 mL) at room temperature. After being stirred for 2 h,

the mixture was stirred under air atmosphere for 30 min until the color of solution was turned to yellow (ca. 30 min), and then a saturated aqueous $NaHCO_3$ solution (2 L) was added. After being stirred for 30 min, FmocOSu (9.00 g, 26.7 mmol) was added. The mixture was stirred for 30 min and the organic layer was separated. The aqueous layer was extracted with EtOAc (2 L x 3). The combined organic layers were dried over anhydrous $MgSO_4$, filtered, and concentrated under reduced pressure. The residue was purified by silica gel flash column chromatography (hexane/EtOAc = 2/1 to 0/1) to afford carbamate **11** (9.26 g, 16.0 mmol, 90%) as pare yellow amorphous material. ^{1}H NMR (400 MHz, $CDCl_3$): δ 7.76 (d, *J* = 7.6 Hz, 2H), 7.58 (d, *J* = 7.6 Hz, 2H), 7.40 (d, *J* = 7.2 Hz, 2H), 7.31 (t, *J* = 7.2 Hz, 2H), 6.78 (br s, 1H), 5.87 (dd, *J* = 18.2, 10.8 Hz, 1H), 5.40 (d, *J* = 10.8 Hz, 1H), 5.33 (d, *J* = 17.6 Hz, 1H), 5.28 (br s, 1H), 4.42 (qn, *J* = 7.6 Hz, 2H), 4.21 (t, *J* = 7.2 Hz, 1H), 4.04 (br s, 1H), 3.86–3.78 (m, 2H), 3.78–3.70 (m, 1H), 3.45–3.25 (m, 2H), 2.55–2.30 (m, 2H), 2.25–2.10 (m, 1H), 1.75–1.50 (m, 1H), 0.89 (s, 9H), 0.07 (s, 6H); ^{13}C NMR (125 MHz, $CDCl_3$): δ 175.87, 156.83, 143.85, 141.27, 137.58, 127.62, 126.99, 124.96, 119.93, 117.01, 76.32, 68.53, 66.62, 63.61, 48.01, 47.22, 43.94, 43.82, 41.83, 33.63, 25.85, 18.11, –5.58, –5.59; IR (KBr): 8261, 2928, 2857, 2360, 1705, 1673, 1519, 1449, 1252, 1106, 910, 837 cm^{-1}; HRMS (ESI, *m/z*): [M+Na]$^+$ calcd for $C_{32}H_{43}O_5N_3NaSi$, 600.2864; found, 600.2868.

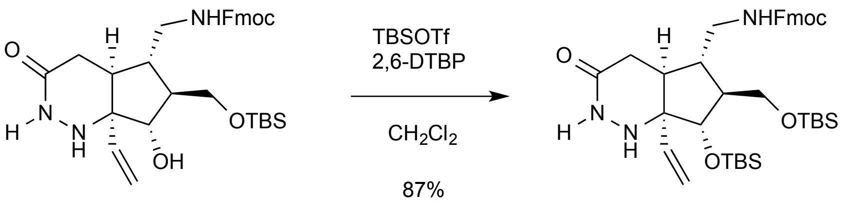

11 **S2**

Compound **S2**:

To a solution of carbamate **11** (9.26 g, 16.0 mmol) in CH_2Cl_2 (160 mL) were added 2,6-DTBP (20.8 mL, 96.0 mmol) and TBSOTf (11.0 mL, 48.0 mmol) at –78 ºC. After being stirred at –78 ºC for 3 h, the reaction was quenched with saturated aqueous $NaHCO_3$ solution (200 mL). The organic layer was separated, and the aqueous layer was extracted with EtOAc (200 mL x3). The combined organic layers were dried over anhydrous $MgSO_4$, filtered, and concentrated under reduced pressure. The residue was purified by silica gel flash column chromatography (hexane/EtOAc = 5/1 to 1/2) to afford silyl ether **S2** (9.63 g, 13.9 mmol, 87%) as a white amorphous material. ^{1}H NMR (400 MHz, $CDCl_3$): δ 7.76 (d, *J* = 7.6 Hz, 2H), 7.59 (d, *J* = 7.6 Hz, 2H), 7.40 (t, *J* = 7.6 Hz, 2H), 7.31 (t, *J* = 7.2 Hz, 2H), 6.63 (br s, 1H), 5.90 (dd, *J* = 17.6, 11.2 Hz, 1H), 5.48 (br s, 1H), 5.24 (d, *J* = 11.2 Hz, 1H),

5.17 (d, J = 17.6 Hz, 1H), 4.40 (t, J = 6.8 Hz, 2H), 4.21 (t, J = 6.8 Hz, 1H), 3.98 (s, 1H), 3.84 (d, J = 10.8 Hz, 1H), 3.77 (d, J = 10.0 Hz, 1H), 3.52 (t, J = 10.0 Hz, 1H), 3.48–3.38 (m, 1H), 3.32–3.22 (m, 1H), 2.52–2.38 (m, 2H), 1.72–1.60 (m, 1H), 1.60–1.45 (m, 1H), 0.90 (s, 9H), 0.89 (s, 9H), 0.08 (s, 3H), 0.06 (s, 6H), 0.03 (s, 3H); ^{13}C NMR (100 MHz, $CDCl_3$): δ 175.66, 157.18, 144.32, 144.28, 141.62, 138.32, 127.93, 127.32, 125.33, 120.25, 115.73, 76.79, 68.50, 66.93, 63.30, 49.28, 47.62, 44.24, 43.50, 41.87, 33.66, 26.21, 26.16, 18.49, 18.35, –3.49, –4.45, –5.23; IR (KBr): 3734, 3271, 2953, 2928, 2856, 2360, 2341, 1714, 1682, 1520, 1471, 1252, 1132, 837 cm^{-1}; HRMS (ESI, *m/z*): $[M+Na]^+$ calcd for $C_{38}H_{57}O_5N_3NaSi_2$, 714.3729; found, 714.3734.

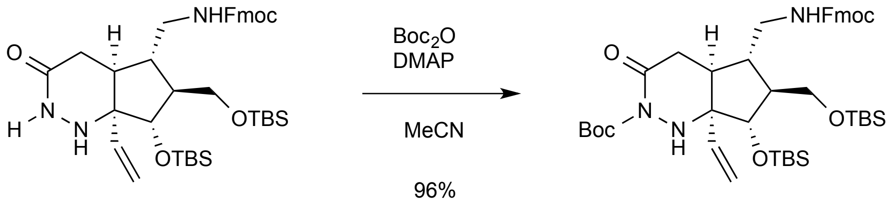

S2 **12**

Compound **12**:

To a solution of silyl ether **S2** (9.63 g, 13.9 mmol) in MeCN (139 mL) were added Boc_2O (3.64 mL, 16.7 mmol) and DMAP (170 mg, 1.39 mmol) at 0 ºC. After being stirred 0 ºC for 2 h, the reaction was quenched with brine (150 mL). The mixture was extracted with EtOAc (200 mL x3). The combined organic layers were dried over anhydrous $MgSO_4$, filtered, and concentrated under reduced pressure. The residue was purified by silica gel flash column chromatography (hexane/EtOAc = 8/1 to 2/1) to afford **12** (10.5 g, 13.3 mmol, 96%) as a white amorphous material. ^1H NMR (400 MHz, $CDCl_3$): δ 7.76 (d, J = 7.6 Hz, 2H), 7.56 (d, J = 7.6 Hz, 2H), 7.39 (t, J = 7.4 Hz, 2H), 7.31 (t, J = 7.4 Hz, 2H), 5.99 (dd, J = 17.6, 11.2 Hz, 1H), 5.45 (br t, 1H), 5.23 (s, 1H), 5.21 (d, J = 11.2 Hz, 1H), 5.18 (d, J = 17.6 Hz, 1H), 4.41 (dd, J = 13.6, 11.2 Hz, 2H), 4.21 (t, J = 11.2 Hz, 1H), 3.79 (d, J = 10.0 Hz, 1H), 3.79 (d, J = 10.0 Hz, 1H), 3.51 (dd, J = 10.0, 6.4 Hz, 1H), 3.45–3.35 (m, 1H), 3.30–3.15 (m, 1H), 2.64 (dd, J = 14.4, 5.6 Hz, 1H), 2.38 (dd, J = 14.4, 8.0 Hz, 1H), 2.24–2.18 (br dd, J = 14.4, 8.0 Hz, 1H), 1.70–1.60 (m, 1H), 1.60–1.50 (m, 1H), 1.54 (s, 9H), 0.92 (s, 9H), 0.89 (s, 9H), 0.19 (s, 3H), 0.07 (s, 3H), 0.06 (s, 6H); ^{13}C NMR (100 MHz, $CDCl_3$): δ 170.16, 156.72, 151.05, 143.91, 141.29, 137.49, 127.61, 127.00, 125.01, 119.93, 115.40, 84.18, 68.51, 66.53, 62.02, 50.04, 47.29, 43.91, 42.28, 41.97, 38.03, 28.01, 25.91, 25.82, 18.19, 18.00, –3.78, –4.97, –5.47, –5.55 (one peak missing in $CDCl_3$); IR (KBr): 3734, 2929, 2360, 2341, 1771, 1717, 1522, 1472, 1252, 1152, 837 cm^{-1}: HRMS (ESI, *m/z*): $[M+Na]^+$ calcd for $C_{43}H_{65}O_7N_3NaSi_2$, 814.4253; found, 814.4251.

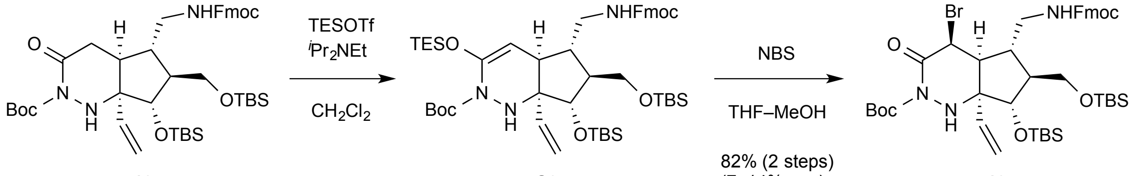

12 S3 13

Compound **13**:

To a solution of **12** (10.5 g, 13.3 mmol) in CH_2Cl_2 (132 mL) were added ^{i}Pr_2NEt (23.6 mL, 132 mmol) and TESOTf (11.6 mL, 66.0 mmol) at –78 ºC. After being stirred at –78 ºC for 3 h, the reaction was quenched with a saturated aqueous $NaHCO_3$ solution (100 mL). The organic layer was separated, and the aqueous layer was extracted with CH_2Cl_2 (150 mL x3). The combined organic layers were dried over anhydrous $MgSO_4$, filtered, and concentrated under reduced pressure to give crude silyl ketene aminal **S3**. The crude **S3** was used for next step without purification. To a solution of residue in THF (66 mL) were added MeOH (66 mL) and NBS (3.05 mL, 17.2 mmol) at –78 ºC. After being stirred at –78 ºC for 1 h, the reaction was quenched with a saturated aqueous $NaHCO_3$ solution (50 mL). The organic layer was separated, and the aqueous layer was extracted with EtOAc (50 mL x3). The combined organic layers were dried over anhydrous $MgSO_4$, filtered, and concentrated under reduced pressure. The residue was purified by silica gel flash column chromatography (hexane/EtOAc = 10/1 to 4/1) to afford bromide **13** (9.41 g, 10.8 mmol, 82% for 2 steps) as a white amorphous material and recovered **12** (1.46 g, 1.85 mmol, 14%).; ^{1}H NMR (400 MHz, $CDCl_3$): δ 7.76 (d, *J* = 7.2 Hz, 2H), 7.59 (dd, *J* = 7.6, 4.8 Hz, 2H), 7.40 (t, *J* = 7.6 Hz, 2H), 7.32 (tdd, *J* = 7.6, 2.8, 1.2 Hz, 2H), 5.91 (dd, *J* = 18.0, 11.2 Hz, 1H), 5.25 (d, *J* = 10.8 Hz, 1H), 5.22 (d, *J* = 18.0 Hz, 1H), 5.14 (br s, 1H), 4.91 (s, 1H), 4.61 (d, *J* = 4.8 Hz, 1H), 4.44 (d, *J* = 6.8 Hz, 2H), 4.21 (t, *J* = 6.8 Hz, 1H), 3.89 (d, *J* = 10.4 Hz, 1H), 3.77 (br d, *J* = 10.0 Hz, 1H), 3.62 (dd, *J* = 10.6, 4.0 Hz, 1H), 3.42–3.28 (m, 2H), 2.68–2.58 (m, 1H), 2.32–2.18 (m, 1H), 1.76–1.62 (m, 1H), 1.52 (s, 9H), 0.91 (s, 9H), 0.90 (s, 9H), 0.21 (s, 3H), 0.09 (s, 3H), 0.06 (s, 6H); ^{13}C NMR (100 MHz, $CDCl_3$): δ 166.13, 156.96, 150.49, 143.95, 143.84, 141.33, 137.34, 127.66, 127.09, 127.07, 124.98, 119.95, 116.57, 84.11, 76.88, 69.17, 66.71, 60.12, 51.06, 48.94, 47.26, 44.64, 41.93, 40.27, 27.96, 25.96, 25.86, 18.22, 18.05, –3.77, –4.85, –5.42, –5.50; IR (KBr): 3734, 3649, 3373, 2928, 2856, 2360, 2341, 1770, 1718, 1523, 1472, 1370, 1253, 1150, 835 cm^{-1}HRMS (ESI, *m/z*): [M+Na]$^+$ calcd for $C_{43}H_{64}O_7N_3BrNaSi_2$, 892.3358; found, 892.3360.

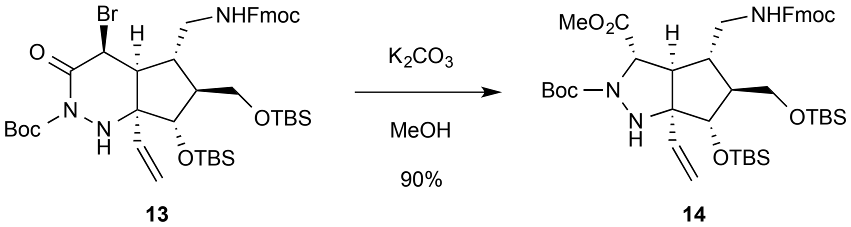

Compound **14**:

To a solution of bromide **13** (9.41 g, 10.8 mmol) in MeOH (108 mL) was added K_2CO_3 (1.64 g, 11.9 mmol) at 0 ºC. After being stirred at 0 °C for 10 min, the reaction was quenched with a saturated aqueous NH_4Cl solution (100 mL) was added. The mixture was extracted with EtOAc (100 mL). The combined organic layers were dried over anhydrous $MgSO_4$, filtered, and concentrated under reduced pressure. The residue was purified by silica gel flash column chromatography (hexane/EtOAc = 10/1 to 4/1) to afford ester **14** (7.99 g, 9.72 mmol, 90%) as a white amorphous material. ^{1}H NMR (400 MHz, $CDCl_3$): δ 7.76 (d, *J* = 7.6 Hz, 2H), 7.61 (d, *J* = 7.6 Hz, 2H), 7.39 (t, *J* = 7.6 Hz, 2H), 7.31 (t, *J* = 7.6 Hz, 2H), 6.10 (d, *J* = 18.0, 11.2 Hz, 1H), 5.61 (br s, 1H), 5.27 (d, *J* = 11.2 Hz, 1H), 5.20 (d, *J* = 18.0 Hz, 1H), 4.50–4.38 (m, 1H), 4.45 (dd, *J* = 10.4, 6.8 Hz, 1H), 4.40 (dd, *J* = 10.4, 6.8 Hz, 1H), 4.21 (m, 1H), 4.21 (s, 1H), 3.75 (dd, *J* = 10.4, 2.4 Hz, 1H), 3.67 (d, *J* = 11.6 Hz, 1H), 3.65 (s, 3H), 3.52 (dd, *J* = 10.4, 6.4 Hz, 1H), 3.45–3.20 (m, 2H), 2.43 (dd, *J* = 6.4, 2.8 Hz, 1H), 1.90–1.75 (m, 1H), 1.65–1.59 (m, 1H), 1.48 (s, 9H), 0.90 (s, 9H), 0.89 (s, 9H), 0.22 (s, 3H), 0.09 (s, 3H), 0.06 (s, 3H), 0.05 (s, 3H); ^{13}C NMR (100 MHz, $CDCl_3$): δ 171.99, 156.58, 144.07, 143.93, 141.32, 141.29, 135.55, 127.62, 127.00, 125.00, 119.91, 116.39, 80.88, 76.32, 66.50, 61.95, 56.58, 52.28, 50.70, 47.31, 44.34, 44.22, 28.30, 25.93, 25.88, 18.23, 17.99, –3.58, –5.26, –5.47, –5.55; IR (KBr): 3724, 3343, 2953, 2929, 2895, 2857, 2360, 2341, 1698, 1521, 1472, 1389, 1366, 1252, 1136, 1005, 938, 837 cm^{-1}; HRMS (ESI, *m/z*): $[M+Na]^+$ calcd for $C_{44}H_{67}O_8N_3NaSi_2$, 844.4359; found, 844.4368.

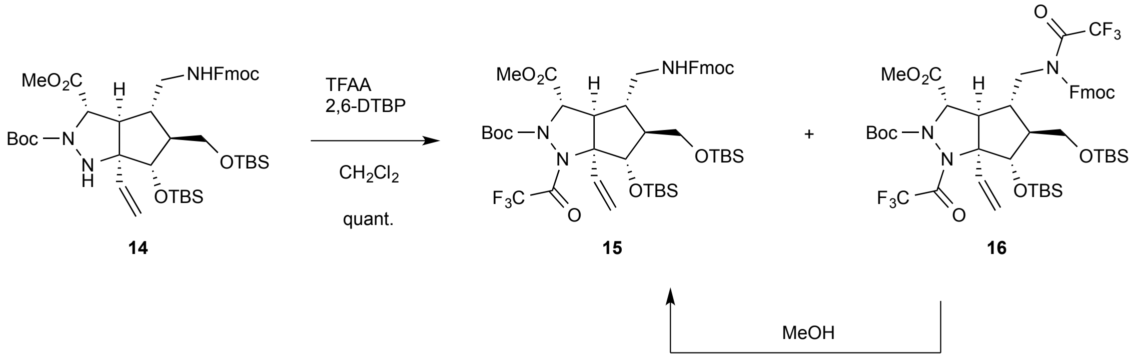

Compound **15**;

To a solution of ester **14** (7.99 g, 9.72 mmol) in CH_2Cl_2 (97 mL) were added 2,6-DTBP (31.5 mL, 145.8 mmol) and TFAA (13.6 mL, 97.2 mmol) at 0 ºC. After being stirred 0 °C for 1 h, a saturated aqueous $NaHCO_3$ solution (100 mL) was added. The organic layer was separated, and the aqueous layer was extracted with EtOAc (100 mL x3). The combined organic layers were dried over anhydrous $MgSO_4$, filtered, and concentrated under reduced pressure. The residue was purified by silica gel flash column chromatography (hexane/EtOAc = 10/1 to 4/1) to afford a mixture of trifluoroacetamide **15** and di-trifluoroacetamide **16**. The mixture of **15** and **16** in MeOH (200 mL) was heated to 40 ºC for 3 h and concentrated under reduced pressure to afford trifluoroacetamide **15** (8.93 g, 9.72 mmol, quant.) as a white amorphous material. ^{1}H NMR (400 MHz, $CDCl_3$, 60 ºC): δ 7.76 (d, *J* = 7.6 Hz, 2H), 7.58 (dd, *J* = 7.2, 4.0 Hz, 2H), 7.39 (t, *J* = 7.6 Hz, 2H), 7.29 (t, *J* = 7.6 Hz, 2H), 6.48 (dd, *J* = 17.6, 11.2 Hz, 1H), 5.65 (br s, 1H), 5.24 (d, *J* = 11.2 Hz, 1H), 5.16 (d, *J* = 17.6 Hz, 1H), 4.83 (br s, 1H), 4.45 (dd, *J* = 17.2, 6.8 Hz, 1H), 4.38 (dd, *J* = 17.2, 7.2 Hz, 1H), 4.22 (t, *J* = 7.2 Hz, 1H), 3.98 (d, *J* = 10.0 Hz, 1H), 3.92 (dd, *J* = 10.4, 2.8 Hz, 1H), 3.69 (s, 3H), 3.52–3.33 (m, 3H), 3.07 (d, *J* = 10.0 Hz, 1H), 1.78–1.65 (m, 1H), 1.55–1.45 (m, 1H), 1.50 (s, 9H), 0.95 (s, 9H), 0.91 (s, 9H), 0.19 (s, 3H), 0.08 (s, 9H); ^{13}C NMR (125 MHz, $CDCl_3$, 60 ºC): δ 169.14, 156.58, 156.28, 155.02 (q, *J* = 35.6 Hz), 144.19, 144.10, 141.48, 141.45, 132.41, 127.72, 127.06, 125.05, 124.98, 120.01, 116.40, 116.01 (q, *J* = 286.9 Hz), 85.00, 79.45, 79.30, 67.01, 65.42, 63.82, 55.89, 53.13, 52.46, 47.50, 44.74, 43.24, 27.89, 26.11, 26.00, 18.33, 18.25, –4.08, –4.58, –5.35, –5.36; IR (KBr): 3734, 2929, 2857, 2360, 2342, 1717, 1508, 1472, 1371, 1253, 1205, 1155, 838 cm^{-1}; HRMS (ESI, *m/z*) [M+Na]$^+$ calcd for $C_{46}H_{66}O_9N_3F_3NaSi_2$, 940.4182; found, 940.4187.

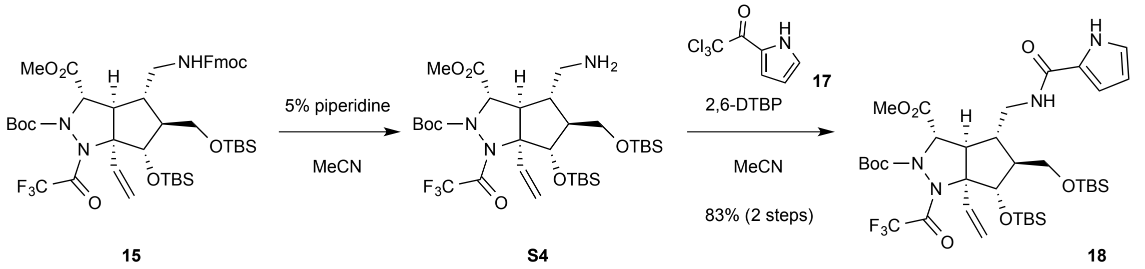

Compound **18**;

To a solution of 5% piperidine in MeCN (194 mL) was added trifluoroacetamide **15** (8.93 g, 9.72 mmol) at room temperature. After being stirred for 10 min, water (200 mL) was added. The mixture was extracted with EtOAc (200 mL x3). The combined organic layers were washed with brine (300 mL), dried over $MgSO_4$, filtered, and concentrated under reduced pressure to give crude **S4**. To a solution of crude **S4** in MeCN (49 mL) were added 2,6-DTBP (5.45 mL, 25.2 mmol) and 2-(trichloroacetyl)pyrrole **17** (2.67 g, 12.6 mmol) at room temperature. The mixture was stirred for 48 h and concentrated under reduced pressure. The residue was purified by silica gel flash column chromatography (hexane/EtOAc = 10/1 to 1/1) to afford pyrrole **18** (6.37 g, 8.07 mmol, 83% for 2 steps) as a white solid material. ^{1}H NMR (400 MHz, $CDCl_3$, 60 ºC): δ 9.23 (br s, 1H), 6.90 (s, 1H), 6.51 (s, 2H), 6.54 (dd, *J* = 17.6, 11.2 Hz, 1H), 6.23 (br d, *J* = 2.3 Hz, 1H), 5.26 (d, *J* = 11.2 Hz, 1H), 5.19 (d, *J* = 17.6 Hz, 1H), 4.85 (br s, 1H), 3.99 (d, *J* = 9.6 Hz, 1H), 3.93 (d, *J* = 10.8 Hz, 1H), 3.85–3.75 (m, 1H), 3.71(s, 3H), 3.51 (dd, *J* = 10.8, 6.8 Hz, 1H), 3.44 (dd, *J* = 12.8, 6.0 Hz, 1H), 3.14 (d, *J* = 8.4 Hz, 1H), 1.75–1.60 (m, 2H), 1.49 (s, 9H), 0.94 (s, 9H), 0.93 (s, 9H), 0.19 (s, 3H), 0.12 (s, 6H), 0.08 (s, 3H); ^{13}C NMR (125 MHz, $CDCl_3$, 60 ºC): δ 169.63, 161.32, 156.14, 155.00 (q, *J* = 37.1 Hz), 132.46, 126.06, 121.50, 116.49, 115.99 (q, *J* = 287.5 Hz), 109.81, 108.98, 84.97, 79.57, 78.85, 65.65, 63.36, 56.12, 52.54, 52.40, 44.16, 41.31, 27.98, 26.08, 26.07, 18.41, 18.22, –4.15, –4.54, –5.24, –5.28; IR (KBr): 3734, 3257, 2954, 2930, 2858, 2360, 2342, 1743, 1636, 1559, 1520, 1472, 1437, 1372, 1254, 1205, 1155, 838 cm^{-1}; HRMS (ESI, *m/z*): $[M+Na]^+$ calcd for $C_{36}H_{59}O_8N_4F_3NaSi_2$, 811.3716; found, 811.3714.

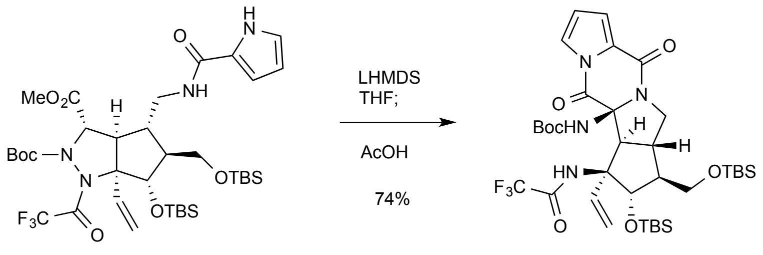

18 **19**

Compound **19**:

To a solution of pyrrole **18** (120 mg, 0.152 mmol) in THF (7.6 mL) was added 1.0 M THF solution of LHMDS (464 μL, 0.464 mmol) at –78 ºC. The mixture was warmed up to 0 °C and the resulting yellow solution was further stirred at this temperature for 10 min. After the mixture was cooled to –78 ºC, 1.0 M THF solution of AcOH (152 μL, 0.152 mmol) was slowly added. The mixture was stirred at room temperature for 3 h. The reaction was quenched with 1.0 M THF solution of AcOH (319 μL, 0.319 mmol), and to the mixture was added brine (10 mL). The organic layer was separated, and the aqueous layer was extracted with EtOAc (10 mL x3). The combined organic layer was dried over anhydrous $MgSO_4$, filtered, and concentrated under reduced pressure. The residue was purified by silica gel flash column chromatography (hexane/EtOAc = 10/1 to 2/1) to afford tetracyclic compound **19** (85.1 mg, 0.112 mmol, 74%) as a white amorphous material. ^{1}H NMR (500 MHz, $CDCl_3$): δ 7.43 (dd, *J* =3.0, 1.5 Hz, 1H), 7.29 (br s, 1H), 7.08 (dd, *J* = 3.0, 1.5 Hz, 1H), 6.48 (t, *J* = 3.0 Hz, 1H), 5.91 (dd, *J* = 17.5, 11.0 Hz, 1H), 5.56 (br s, 1H), 5.31 (d, *J* = 11.0 Hz, 1H), 5.08 (d, *J* = 17.5 Hz, 1H), 4.98 (d, *J* = 7.5 Hz, 1H), 3.88 (dd, *J* = 11.5, 8.0 Hz, 1H), 3.88 (dd, *J* = 10.3, 7.5 Hz, 1H), 3.61 (dd, *J* = 10.3, 7.5 Hz, 1H), 3.45 (dd, *J* = 11.5, 10.0 Hz, 1H), 2.87-2.75 (m, 1H), 2.45 (d, *J* = 14.5 Hz, 1H), 1.88 (dtd, *J* = 11.0, 7.5, 3.5 Hz, 1H), 1.30 (s, 9H), 0.91 (s, 9H), 0.86 (s, 9H), 0.07 (s, 3H), 0.07 (s, 3H), 0.06 (s, 3H), 0.04 (s, 3H); ^{13}C NMR (125 MHz, $CDCl_3$): δ 161.85, 157.47 (q, *J* = 36.4 Hz), 156.61, 153.21, 136.43, 126.34, 120.02, 118.72, 115.73 (q, *J* = 287.4 Hz), 115.67, 113.89, 83.01, 82.23, 74.16, 65.16, 62.99, 60.86, 52.89, 46.54, 40.82, 27.76, 25.82, 25.75, 18.22, 17.97, –4.04, –5.20, –5.45, –5.54; IR (KBr): 3372, 2954, 2930, 2858, 2360, 2342, 1734, 1653, 1472, 1419, 1369, 1254, 1158, 1108, 1107, 837 cm^{-1}; HRMS (ESI, *m/z*): [M–H] calcd for $C_{35}H_{54}O_7N_4F_3Si_2$, 755.3489; found, 755.3499.

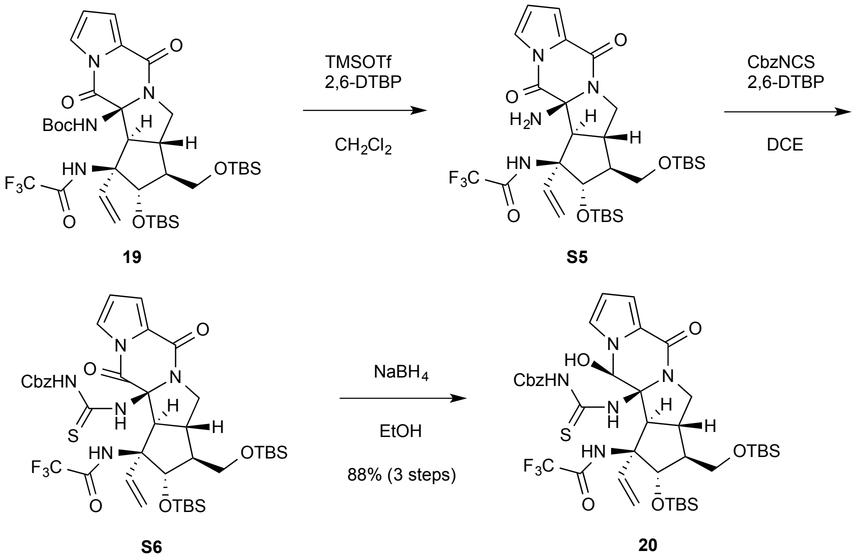

Compound **20**:

To a solution of tetracyclic compound **19** (681 mg, 0.900 mmol) in CH_2Cl_2 (18 mL) were added 2,6-DTBP (1.17 mL, 5.40 mmol) and TMSOTf (812 μL, 4.50 mmol) at 0 ºC. After being stirred for 30 min at room temperature, the reaction was quenched with a saturated aqueous $NaHCO_3$ solution (20 mL). The organic layer was separated, and the aqueous layer was extracted with EtOAc (20 mL x3). The combined organic layers were dried over anhydrous $MgSO_4$, filtered, and concentrated under reduced pressure to give crude **S5**. The crude amine **S5** was used for the next step without purification due to its instability. To a solution of amine **S5** in DCE (18 mL) were added 2,6-DTBP (389 μL, 1.8 mmol) and CbzNCS (869 mg, 4.50 mmol) at room temperature. The mixture was heated to 70 ºC for 12 h and concentrated under reduced pressure to give crude **S6**. The crude thiourea **S6** was also used for the next step without purification. A solution of thiourea **S6** in EtOH (18 mL) was stirred at room temperature until remaining excess CbzNCS disappeared. The mixture was cooled to 0 ºC and $NaBH_4$ (37.0 mg, 0.990 mmol) was added. After being stirred for 30 min, brine (20 mL) was added. The mixture was extracted with EtOAc (20 mL x3). The combined organic layers were dried over anhydrous $MgSO_4$, filtered, and concentrated under reduced pressure. The residue was purified by silica gel flash column chromatography ($CHCl_3$/EtOAc = 1/0 to 4/1) to afford alcohol **20** (675 mg, 0.792 mmol, 88%) as a white amorphous material. ^1H NMR (500 MHz, $CDCl_3$): δ 10.80 (s, 1H), 7.89 (s, 1H), 7.39–7.34 (m, 3H), 7.32–7.26 (m, 2H), 7.17 (s, 1H), 7.02 (dd, *J* = 2.5, 1.5 Hz, 1H), 6.92 (dd, *J* = 3.5, 1.5 Hz, 1H), 6.26 (dd, *J* = 17.5, 11.0 Hz, 1H), 6.24 (dd, *J* = 3.5, 2.5 Hz, 1H), 5.70 (d, *J* = 2.5 Hz, 1H), 5.53 (d, *J* = 11.0 Hz, 1H), 5.25 (d, *J* = 17.5 Hz, 1H), 5.15 (d, *J* = 12.0 Hz, 1H), 5.10 (d, *J* = 12.0 Hz, 1H), 4.65 (d, *J* = 4.5 Hz, 1H), 4.23 (dd, *J* = 10.0, 8.0 Hz, 1H), 4.13 (br s, 1H), 3.74 (dd, *J* = 10.5, 3.5 Hz, 1H), 3.63 (dd, *J* = 10.0, 5.0 Hz, 1H), 3.40–3.20 (m,

1H), 3.13 (t, J = 10.0 Hz, 1H), 2.69 (d, J = 14.0 Hz, 1H), 1.90–1.83 (m, 1H), 0.91 (s, 9H), 0.87 (s, 9H), 0.14 (s, 3H), 0.09 (s, 3H), 0.05 (s, 3H), 0.05 (s, 3H); ^{13}C NMR (125 MHz, $CDCl_3$): δ 178.39, 157.17, 155.94 (q, J = 35.1 Hz), 152.24, 135.84, 133.96, 128.88, 128.71, 128.69, 128.21, 122.28, 121.57, 121.55, 115.51 (q, J = 116.3 Hz), 110.71, 85.19, 82.94, 77.62, 68.44, 66.02, 63.94, 61.46, 53.38, 47.49, 40.65, 25.85, 25.84, 18.32, 17.94, –4.03, –5.17, –5.60, –5.61; IR (KBr): 3734, 2930, 2857, 2360, 2341, 1732, 1623, 1541, 1471, 1417, 1210, 835 cm^{-1}; HRMS (APCI, *m/z*): [M–H] calcd for $C_{39}H_{55}O_7N_5F_3SSi_2$, 850.3318; found, 850.3335.

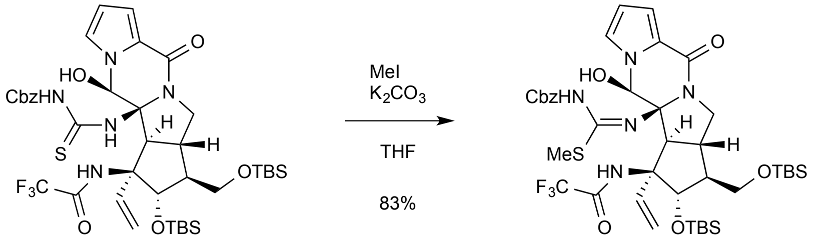

20 **21**

Compound **21**;

To a solution of **20** (675 mg, 0.792 mmol) in THF (7.9 mL) were added MeI (297 μL, 4.75 mmol) and K_2CO_3 (656 mg, 4.75 mmol) at 0 ºC. After being stirred for 1 h, the reaction was quenched with brine (10 mL). The mixture was extracted with EtOAc (10 mL x3). The combined organic layers were dried over anhydrous $MgSO_4$, filtered, and concentrated under reduced pressure. The residue was purified by silica gel flash column chromatography (hexane/EtOAc = 5/1 to 1/1) to afford isothiourea **21** (569 mg, 0.657 mmol, 83%) as a white crystal. mp (from ethanol): 151–153 ºC; ^1H NMR (500 MHz, $CDCl_3$): δ 8.51 (br s, 1H), 7.45–7.30 (m, 5H), 7.08 (dd, J = 3.0, 1.5 Hz, 1H), 6.88 (dd, J = 3.5, 1.5 Hz, 1H), 6.81 (br s, 1H), 6.21 (dd, J = 3.5, 3.0 Hz, 1H), 5.99 (dd, J = 17.5, 11.0 Hz, 1H), 5.57 (d, J = 12.5 Hz, 1H), 5.53 (d, J = 12.5 Hz, 1H), 5.34 (d, J = 11.0 Hz, 1H), 5.13 (d, J = 1.5 Hz, 1H), 5.08 (d, J = 17.5 Hz, 1H), 4.77 (d, J = 5.0 Hz, 1H), 3.85 (dd, J = 11.0, 8.0 Hz, 1H), 3.77 (dd, J = 10.0, 3.5 Hz, 1H), 3.58 (dd, J =10.0, 5.5 Hz, 1H), 3.23 (t, J = 11.0 Hz, 1H), 2.56 (d, J = 14.0 Hz, 1H), 2.48–2.35 (m, 1H), 2.29 (s, 3H), 1.87 (dddd, J = 10.5, 5.5, 5.0, 3.5 Hz, 1H), 0.89 (s, 9H), 0.86 (s, 9H), 0.15 (s, 3H), 0.07 (s, 3H), 0.03 (s, 6H); ^{13}C NMR (125 MHz, $CDCl_3$): δ 157.72, 155.97 (q, J = 36.0 Hz), 151.54, 151.13, 134.21, 134.15, 128.99, 128.84, 128.74, 122.98, 121.75, 115.83 (q, J = 288.0 Hz), 114.52, 114.14, 110.43, 84.76, 83.37, 80.65, 68.54, 66.19, 62.61, 61.79, 53.44, 46.17, 40.19, 25.82, 25.68, 18.13, 17.99, 14.61, –3.95, –5.41, –5.53, –5.69; IR (KBr): 3220, 2953, 2857, 2360, 2410, 1732, 1645, 1542, 1472, 1416, 1387, 1222, 1005, 835 cm^{-1}; HRMS (ESI, *m/z*): $[M+H]^+$ calcd for $C_{40}H_{58}O_7N_5F_3NaSSi_2$, 888.3440; found, 888.3446.

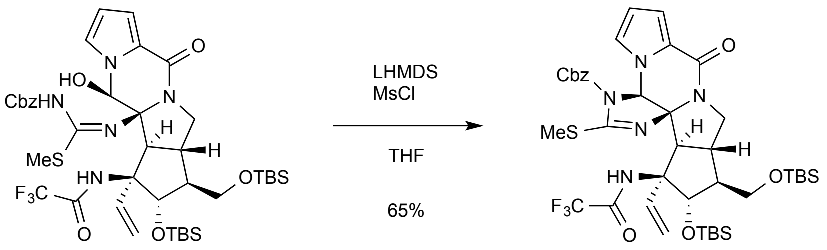

21 22

Compound **22**;

To a solution of isothiourea **21** (569 mg, 0.657 mmol) in THF (6.6 mL) was added 1.0 M THF solution of LHMDS (2.63 mL, 2.63 mmol) at –78 ºC. After being stirred for 30 min, MsCl (236 μL, 2.63 mmol) was added. After being stirred for 1 h at –40 ºC, the reaction was quenched with a saturated aqueous $NaHCO_3$ solution (10 mL). The mixture was extracted with EtOAc (10 mL x3). The combined organic layers were washed with brine (20 mL), dried over anhydrous $MgSO_4$, filtered, and concentrated under reduced pressure. The residue was purified by silica gel flash column chromatography (hexane/EtOAc = 10/1 to 2/1) to afford pentacyclic **22** (362 mg, 0.427 mmol, 65%) as a white amorphous material. ^{1}H NMR (500 MHz, $CDCl_3$, 60 ºC, as a mixture of romater): δ 7.48–7.38 (m, 5H), 7.36 (s, 1H), 6.94 (br s, 1H), 6.92 (dd, *J* = 3.5 1.5 Hz, 1H), 6.25 (t, *J* = 3.5 Hz, 1H), 6.15 (s, 1H), 5.90 (dd, *J* = 17.5, 11.0 Hz, 1H), 5.35 (d, *J* = 12.0 Hz, 1H), 5.29 (d, *J* = 12.0 Hz, 1H), 4.77 (d, *J* = 10.5 Hz, 1H), 4.76 (d, *J* = 18.0 Hz, 1H), 4.58 (d, *J* = 3.0 Hz, 1H), 4.19 (dd, *J* = 11.0, 8.5 Hz, 1H), 3.83 (dd, *J* =10.5, 4.5 Hz, 1H), 3.52 (dd, *J* = 10.0, 7.5 Hz, 1H), 3.31 (t, *J* = 11.0 Hz, 1H), 3.01–2.89 (m, 1H), 2.72 (d, *J* = 14.0 Hz, 1H), 2.41 (s, 3H), 1.93–1.85 (m, 1H), 0.92 (s, 9H), 0.90 (s, 9H), 0.11 (s, 3H), 0.08 (s, 3H), 0.08 (s, 3H), 0.07 (s, 3H); ^{13}C NMR (125 MHz, $CDCl_3$, 60 ºC, as a mixture of rotamer): δ 163.23, 156.27, 155.94 (q, *J* = 36.4 Hz), 149.78, 134.16, 133.01, 129.25, 128.92, 128.85, 124.15, 122.64, 115.71 (q, *J* = 287.9 Hz), 114.13, 113.61, 112.36, 87.41, 84.52, 71.91, 69.52, 66.20, 63.77, 63.33, 54.08, 46.88, 41.30, 25.92, 25.82, 18.37, 17.89, 15.19, –4.27, –4.96, –5.50, –5.54 (some peaks are broadened due to the rotamer.); IR (KBr): 2930, 2360, 2342, 1733, 1654, 1559, 1472, 1421, 1388, 1287, 1158, 837 cm^{-1}; HRMS (ESI, *m/z*): $[M+Na]^+$ calcd for $C_{40}H_{56}O_6N_5F_3NaSSi_2$, 870.3334; found, 870.3348.

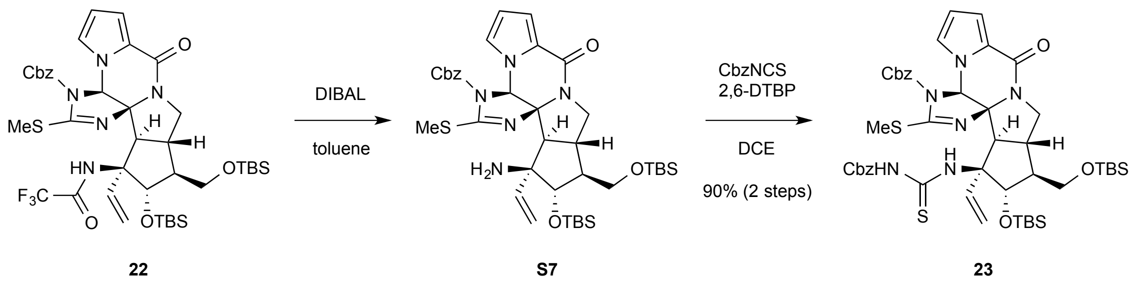

Compound **23**;

To a solution of pentacyclic **22** (362 mg, 0.427 mmol) in toluene (8.5 mL) was added 1.0 M toluene solution of DIBAL (897 μL, 0.897 mmol) at –78 ºC. After being stirred for 10 min, a saturated aqueous NH_4Cl solution (900 μL) and ether (20 mL) were added. After being stirred at room temperature for 1 h, to the mixture was added anhydrous $MgSO_4$. The mixture was vigorously stirred for 1 h, filtered, and concentrated under reduced pressure to give a crude **S7**, which was used for the next step without purification. To a solution of crude **S7** in DCE (8.5 mL) were added CbzNCS (165 mg, 0.854 mmol) and 2,6-DTBP (92 μL, 0.427 mmol) at room temperature. The mixture was stirred for 2 h and concentrated under reduced pressure. The residue was purified by silica gel, previously treated with *N*,*N*-dimethylaniline, column chromatography (hexane/EtOAc = 10/1 to 2/1) to afford thiourea **23** (363 mg, 0.384 mmol, 90% for 2 steps) as a yellow amorphous material. The thioura **23** was needed to immediately use for the next step due to its instability. ^{1}H NMR (500 MHz, C_6D_6, 60 ºC, as a mixture of romater): δ 9.78 (s, 1H), 7.50 (s, 1H), 7.32–6.95 (m, 12H), 6.17 (dd, *J* = 18.0, 11.5 Hz, 1H), 6.13 (t, *J* = 3.0 Hz, 1H), 5.29 (d, *J* = 2.5 Hz, 1H), 5.14 (d, *J* = 18.0 Hz, 1H), 5.05–4.92 (m, 4H), 4.78 (d, *J* = 12.0 Hz, 1H), 4.71 (d, *J* = 12.5 Hz, 1H), 4.38 (dd, *J* =10.5, 8.0 Hz, 1H), 3.85 (dd, *J* = 9.5, 5.0 Hz, 1H), 3.62 (dd, *J* = 9.5, 8.5 Hz, 1H), 3.30 (t, *J* = 10.5 Hz, 1H), 3.28–3.15 (m, 1H), 2.55 (d, *J* = 14.0 Hz, 1H), 1.95 (s, 3H), 1.04 (s, 9H), 0.92 (s, 9H), 0.37 (s, 3H), 0.28 (s, 3H), 0.05 (s, 3H), 0.04 (s, 3H); IR (KBr): 3734, 3628, 2929, 2360, 2341, 1732, 1646, 1591, 1558, 1522, 1388, 1348, 1286, 1103, 837 cm^{-1};HRMS (ESI, *m/z*): [M–H] calcd for $C_{47}H_{63}O_7N_6S_2Si_2$, 943.3744; found, 943.3763. Since thiourea **23** was gradually decomposed during the NMR experiment at 60 °C, the spectra of time-consuming ^{13}C-NMR was difficult to obtain. Thus, we added a direct chart of HRMS.

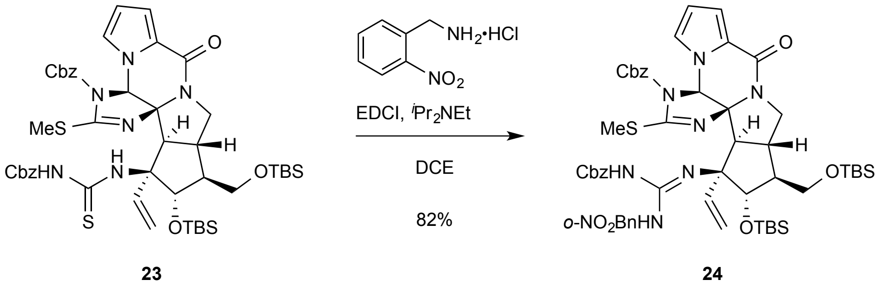

Compound **24**;

To a solution of thiourea **23** (363 mg, 0.384 mmol) in DCE (7.7 mL) were added $^{i}Pr_2NEt$ (336 μL, 2.30 mmol), *o*-nitrobenzylaminehydrochloride (350 mg, 2.30 mmol) and EDCI (440 mg, 2.30 mmol) in this order at room temperature. After being stirred at 50 ºC for 12 h, the reaction was cooled to room temperature and quenched with a saturated aqueous NH_4Cl solution (10 mL). The mixture was extracted with EtOAc (10 mL x3). The combined organic layers were dried over anhydrous $MgSO_4$, filtered, and concentrated under reduced pressure. The residue was purified by silica gel flash column chromatography (hexane/EtOAc = 10/1 to 1/1) to afford guanidine **24** (335 mg, 0.315 mmol, 82%) as a white amorphous material. ^{1}H NMR (500 MHz, $CDCl_3$, 60 ºC, as a mixture of rotamer): δ 9.26 (br s, 1H), 8.03 (d, *J* = 8.0 Hz, 1H), 7.76 (d, *J* = 7.5 Hz, 1H), 7.60 (t, *J* = 7.5 Hz, 1H), 7.47 (t, *J* = 7.0 Hz, 1H), 7.45–7.35 (m, 7H), 7.32 (t, *J* = 7.5 Hz, 2H), 7.26 (t, *J* = 7.5 Hz, 1H), 6.93–6.87 (m, 2H), 6.21 (t, *J* = 3.5 Hz, 1H), 6.18 (dd, *J* = 18.0, 11.0 Hz, 1H), 6.02 (s, 1H), 5.30 (d, *J* = 13.0 Hz, 1H), 5.27 (d, *J* = 12.0 Hz, 1H), 5.19 (d, *J* = 13.0 Hz, 1H), 5.11 (d, *J* = 11.0 Hz, 1H), 5.09 (d, *J* = 13.0 Hz, 1H), 5.02 (d, *J* = 18.0 Hz, 1H), 4.93 (br d, *J* = 10.5 Hz, 1H), 4.82 (dd, *J* = 14.5, 7.0 Hz, 1H), 4.66 (dd, *J* = 14.5, 5.0 Hz, 1H), 4.30–4.20 (m, 2H), 3.64 (dd, *J* = 10.0, 8.0 Hz, 1H), 3.56 (dd, *J* = 10.0, 8.5 Hz, 1H), 3.28 (t, *J* = 10.5 Hz, 1H), 3.24–3.14 (m, 1H), 2.73 (d, *J* = 14.0 Hz, 1H), 2.33 (s, 3H), 1.98–1.89 (m, 1H), 0.92 (s, 9H), 0.88 (s, 9H), 0.07 (s, 3H), 0.07 (s, 3H), 0.03 (s, 3H), 0.02 (s, 3H); ^{13}C NMR (125 MHz, $CDCl_3$, 60 ºC, as a mixture of rotamer): δ 163.61, 159.44, 156.40, 150.10, 148.99, 137.89, 134.66, 134.44, 133.89, 133.72, 133.46, 129.21, 129.07, 128.99, 128.93, 128.89, 128.34, 127.67, 127.55, 124.94, 124.44, 122.56, 117.78, 113.35, 112.14, 87.11, 85.70, 72.04, 69.23, 66.42, 66.40, 65.60, 64.60, 53.31, 47.41, 42.61, 41.85, 26.16, 25.86, 18.52, 17.94, 14.95, –4.20, –4.97, –5.25, –5.35 (some peaks are broadened due to the rotamer.); IR (KBr): 3734, 3628, 2929, 2360, 2341, 1732, 1646, 1591, 1558, 1522, 1388, 1348, 1286, 1103, 837 cm^{-1}; HRMS (ESI, *m/z*): $[M+H]^+$ calcd for $C_{54}H_{71}O_9N_8SSi_2$, 1063.4598; found, 1063.4606.

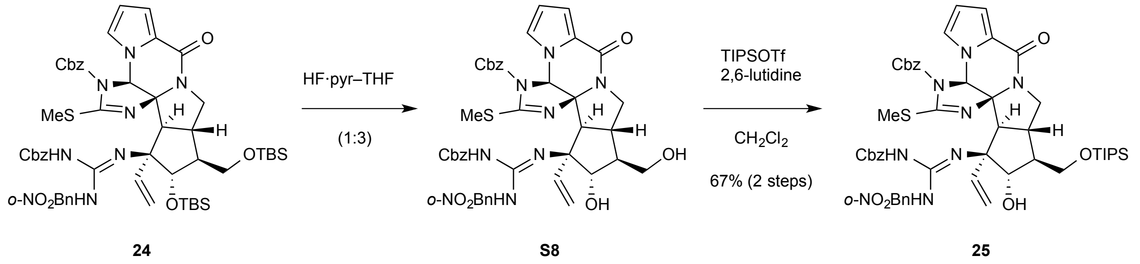

Compound **25**;

To a mixture of HF·pyr–THF (1:3, 6.3 mL) was added guanidine **24** (335 mg, 0.315 mmol) at room temperature. The mixture was stirred for 50 h and cooled to 0 ºC. After the addition of TMSOMe (12 mL), the mixture was concentrated under reduced pressure. The crude diol **S8** was used for the next step without purification. To the solution of crude **S8** in CH_2Cl_2 (6.3 mL) were added 2,6-lutidine (142 μL, 1.26 mmol) and TIPSOTf (169 μL, 0.630 mmol) at 0 ºC. After being stirred at room temperature for 1 h, a saturated aqueous $NaHCO_3$ solution (10 mL) was added. The mixture was extracted with EtOAc (10 mL x3). The combined organic layers were dried over anhydrous $MgSO_4$, filtered, and concentrated under reduced pressure. The residue was purified by silica gel flash column chromatography (hexane/EtOAc = 4/1 to 1/2) to afford silyl ether **25** (209 mg, 0.211 mmol, 67% for 2 steps) as a yellow amorphous material. ^{1}H NMR (500 MHz, CD_3CN): δ 9.20 (s, 1H), 8.04 (d, J = 8.0 Hz, 1H), 7.66 (t, J = 7.5 Hz, 1H), 7.57–7.47 (m, 4H), 7.48–7.38 (m, 3H), 7.37–7.26 (m, 5H), 6.95 (br s, 1H), 6.71 (dd, J = 4.0, 1.5 Hz, 1H), 6.23 (br s, 1H), 6.18–6.05 (m, 1H), 6.14 (s, 1H), 5.51 (t, J = 6.0 Hz, 1H), 5.33 (d, J = 12.0 Hz, 1H), 5.29 (d, J = 12.0 Hz, 1H), 5.02 (d, J = 13.0 Hz, 1H), 4.99 (d, J = 13.0 Hz, 1H), 5.02–4.95 (m, 2H), 4.80 (dd, J = 15.5, 7.0 Hz, 1H), 4.62 (dd, J = 15.5, 5.0 Hz, 1H), 4.07 (t, J = 4.0 Hz, 1H), 3.97 (dd, J = 10.5, 8.0 Hz, 1H), 3.80–3.75 (m, 2H), 3.58 (t, J = 9.5 Hz, 1H), 3.25 (t, J = 10.5 Hz, 1H), 3.07–2.95 (m, 1H), 2.84 (d, J = 14.5 Hz, 1H), 2.30 (s, 3H), 1.93–1.85 (m, 1H), 1.12–1.03 (m, 21H); ^{13}C NMR (125 MHz, CD_3CN): δ 164.00, 160.09, 156.71, 148.94, 138.67, 135.53, 135.33, 134.91, 134.37, 131.72, 129.81, 129.60, 129.39, 129.20, 129.03, 128.21, 128.16, 125.54, 124.86, 123.34, 118.32, 117.51, 112.94, 112.43, 88.05, 86.48, 72.32, 69.68, 66.62, 66.03, 65.47, 64.29, 53.63, 47.47, 42.80, 41.48, 18.17, 14.93, 12.39 (one peak missing in CD_3CN) (some peaks are broadened due to the rotamer); IR (KBr): 3734, 3628, 2942, 2360, 2342, 1733, 1646, 1590, 1558, 1523, 1388, 1348, 1288, 1093 cm^{-1}; HRMS (ESI, *m/z*): $[M+H]^+$ calcd for $C_{51}H_{63}O_9N_8SSi$, 991.4203; found, 991.4212.

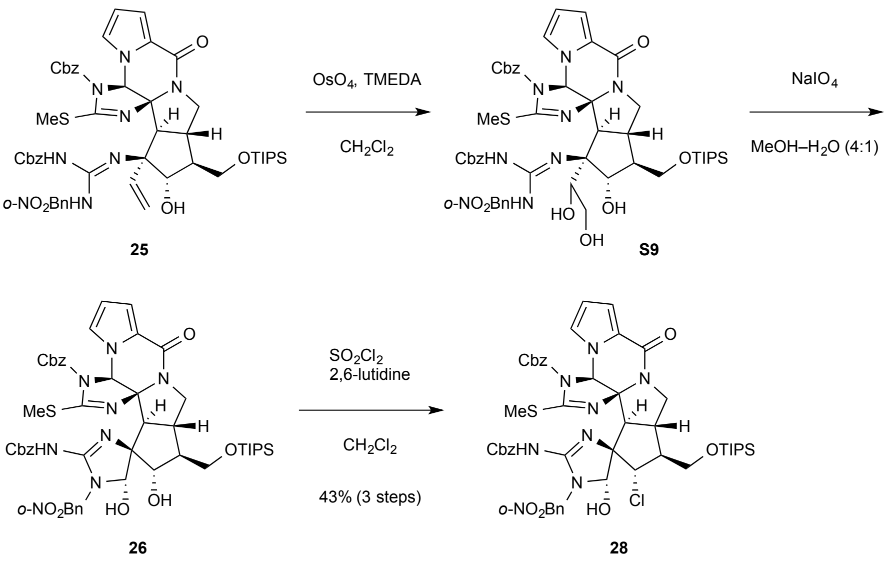

Compound **28**;

To a solution of silyl ether **25** (44.0 mg, 44.4 μmol) were added 0.40 M CH_2Cl_2 solution of OsO_4 (121 μL, 48.4 μmol) and TMEDA (7.2 μL, 48.4 μmol) at –78 ºC. The mixture was stirred for 10 min and warmed to room temperature. To the mixture were added MeOH (1 mL) and 1N HCl (0.2 mL). The mixture was stirred, in the flask wrapped with foil, for 3 h. To the mixture was added 1M solution of Na_2SO_3, and the mixture was extracted with EtOAc (2 mL x3). The combined organic layers were washed with saturated aqueous $NaHCO_3$ (2 mL), dried over anhydrous $MgSO_4$, filtered, and concentrated under reduced pressure to give crude diol **S9**, which was used for the next step without purification. To a solution of crude **S9** in MeOH (2.0 mL) and H_2O (0.5 mL) was added $NaIO_4$ (47.5 mg, 222 μmol) at room temperature. After being stirred under dark condition for 1 h, a saturated aqueous $NaHCO_3$ solution (2 mL) was added. The mixture was extracted with EtOAc (2 mL x3). The combined organic layers were dried over anhydrous $MgSO_4$, filtered, and concentrated under reduced pressure. Azeotropic treatment with hexane provided crude cyclic hemi-aminal **26** (35.2 mg) and it was used for the next step without purification. To a solution of cyclic hemi-aminal **26** in CH_2Cl_2 (1.8 mL) were slowly added 2,6-lutidine (12.3 μL, 106 μmol) and 0.50 M of solution of SO_2Cl_2 (78.0 μL, 39.0 μmol) at 0 ºC, successively. After being stirred for 10 min, a saturated aqueous $NaHCO_3$ solution (2 mL) was added. The mixture was extracted with EtOAc (2 mL x3). The combined organic layers were dried over anhydrous $MgSO_4$, filtered, and concentrated under reduced pressure. The residue was purified by silica gel, previously treated with *N*,*N*-dimethylaniline, flash column chromatography (hexane/EtOAc = 4/1 to 1/2) to afford chloride **28** (18.9 mg, 18.6 μmol, 42% for 3 steps) along with inseparable minor diastereomer at C20 position as a pale yellow amorphous material. ^1H NMR (500 MHz, CD_3CN, 60 ºC): δ 7.99 (d, *J* = 8.0 Hz, 1H), 7.62–7.25 (m,

13H), 6.99 (brs, 1H), 6.94 (br s, 1H), 6.67 (dd, *J* = 3.5, 1.5 Hz, 1H), 6.60 (s,1H), 6.19 (t, *J* = 3.5 Hz, 1H), 5.95 (s,1H), 5.35 (d, *J* = 12.5 Hz, 1H), 5.26 (d, *J* = 12.5 Hz, 1H), 5.24–4.95 (m, 4H), 4.49 (br d, *J* = 13.5 Hz, 1H), 4.02–3.78 (m, 3H), 3.74 (dd, *J* = 10.0, 6.0 Hz, 1H), 3.25–3.12 (m, 2H), 2.75 (d, *J* = 13.5 Hz, 1H), 2.32 (s, 3H), 2.10–2.05 (m, 1H), 1.12–0.98 (s, 21H); IR (KBr): 3734, 3628, 3383, 2961, 2865, 2360, 2342, 1717, 1636, 1577, 1556, 1523, 1427, 1395, 1351, 1289, 11367, 1099, 1023, 881, 801 cm^{-1}; HRMS (ESI, *m/z*): $[M+H]^+$ calcd for $C_{50}H_{60}O_9N_8ClSSi$, 1011.3656; found, 1011.3664. Since nitrobenzyl group of **28** was readily removed during the NMR experiment at 60 °C, the spectra of time-consuming ^{13}C NMR was difficult to obtain. Thus, we added a chart of HRMS.

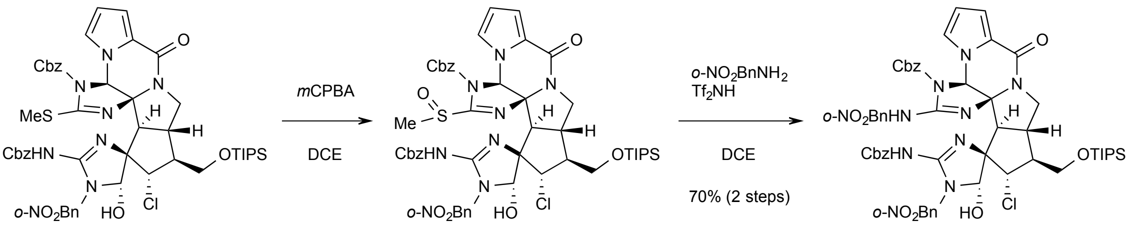

28 **29** **30**

Compound **30**;

To a solution of chloride **28** (18.9 mg, 18.6 μmol) in DCE (370 μL) was added 0.50 M CH_2Cl_2 solution of *m*CPBA (washed with phosphate buffer, 74.4 μL, 37.2 μmol) at 0 ºC. The reaction flask was wrapped with foil to protect nitrobenzyl group. After being stirred at 0 ºC for 3 h, 1M solution of Na_2SO_3 (3 drops) was added. After being stirred for 10 min, a saturated aqueous $NaHCO_3$ solution (1 mL) was added. The mixture was extracted with EtOAc (1 mL x3). The combined organic layers were dried over anhydrous $MgSO_4$, filtered, and concentrated under reduced pressure. The crude sulfoxide **29** was used for the next step without purification. To a solution of crude **29** in DCE (0.9 mL) were slowly added a 0.5 M DCE solution of *o*-NO_2BnNH_2 (112 μL, 55.8 μmol) and a 0.5 M DCE solution of Tf_2NH (112 μL, 55.8 mmol) at 0 ºC. The reaction flask was wrapped with foil to exclude light. After being stirred at 50 ºC for 2 h, a saturated aqueous $NaHCO_3$ solution (1 mL) was added. The mixture was extracted with EtOAc (1 mL x3). The combined organic layers were dried over anhydrous $MgSO_4$, filtered, and concentrated under reduced pressure. The residue was purified by silica gel, previously treated with *N*,*N*-dimethylaniline, flash column chromatography (hexane/EtOAc = 4/1 to 1/2) afforded guanidine **30** (14.5 mg, 13.0 μmol, 70%) along with inseparable minor diastereomer at C20 position as a pale yellow amorphous material. ^1H NMR (500 MHz, CD_3CN, 60 ºC): δ 7.97 (d, *J* = 8.0 Hz, 1H), 7.89 (d, *J* = 8.0 Hz, 1H), 7.65–7.30 (m, 16H), 7.00 (brs, 1H), 6.89 (br s, 1H), 6.61 (dd, *J* = 4.0, 1.5 Hz, 1H), 6.57 (s, 1H), 6.17 (t, *J* = 3.0 Hz,

1H), 5.91 (d, J = 5.0 Hz, 1H), 5.38–5.20 (m, 2H), 5.35 (d, J = 12.5 Hz, 1H), 5.24 (d, J = 12.5 Hz, 1H), 5.16–5.08 (m, 2H), 5.00–4.89 (m, 2H), 4.86 (dd, J = 16.0, 6.5 Hz, 1H), 4.56 (dd, J = 16.5, 5.5 Hz, 1H), 3.93–3.88 (m, 2H), 3.83 (d, J = 3.0 Hz, 1H), 3.79 (d, J = 10.0, 6.0 Hz, 1H), 3.22–3.12 (m, 2H), 2.70 (d, J = 13.0 Hz, 1H), 2.10–1.90 (m, 1H), 1.14–1.05 (m, 21H); IR (KBr): 3734, 3638, 3383, 2961, 2865, 2360, 2342, 1730, 1635, 1523, 1396, 1339, 1289, 1261, 1101, 1017, 800 cm^{-1}; HRMS (ESI, *m/z*): $[M+H]^+$ calcd for $C_{56}H_{64}O_{11}N_{10}ClSi$, 1115.4208; found, 1115.4208. Since nitrobenzyl group of **30** was readily removed during the NMR experiment at 60 °C, the spectra of time-consuming ^{13}C NMR was difficult to obtain. Thus, we added a chart of HRMS.

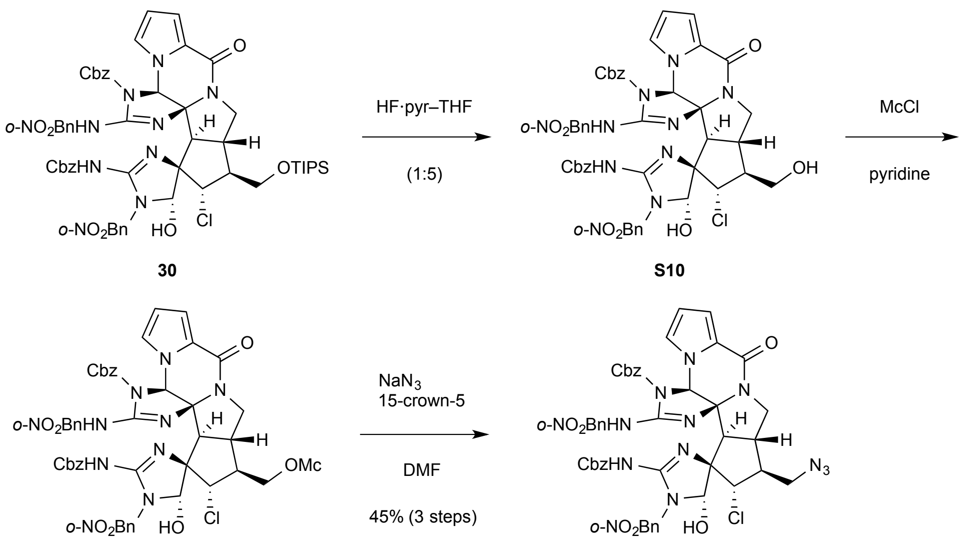

30 **S10** **31** **32**

Compound **32**;

Guanidine **30** (14.5 mg, 13.0 μmol) was added HF·pyr–THF (1:5, 260 μL) at room temperature. The reaction flask was rapped with foil to protect nitrobenzyl group. The mixture was stirred for 3 h and cooled to 0 ºC. After the addition of TMSOMe (1 mL), the mixture was concentrated under reduced pressure. The crude alcohol **S10** was used for the next step without purification. To a solution of crude **S10** in pyridine (260 μL) was slowly added chloromethylsulfonyl chloride (2.3 μL, 26.0 μmol) at 0 ºC. After being stirred at 0 ºC for 10 min, a saturated aqueous $NaHCO_3$ solution (1 mL) was added. The mixture was extracted with EtOAc (1 mL x3). The combined organic layers were dried over anhydrous $MgSO_4$, filtered, and concentrated under reduced pressure. The crude chloromethanesulfonylate **31** was used for the next step without purification. To a solution of crude **31** in DMF (260 μL) were slowly added a 1.0 M DMSO solution of NaN_3 (52.0 μL, 26.0 μmol) and 15-crown-5 (5.1 μL, 26.0 μmol) at room temperature. The reaction flask was wrapped with foil to

exclude light. After being stirred for 15 h, a saturated aqueous $NaHCO_3$ solution (1 mL) was added. The mixture was extracted with EtOAc (1 mL x3). The combined organic layers were dried over anhydrous $MgSO_4$, filtered, and concentrated under reduced pressure. The residue was purified by PTLC (hexane/EtOAc = 1/4) to afforded azide **32** (5.7 mg, 58.5 μmol, 45% for 3 steps) as a pale yellow amorphous material. ^{1}H NMR (500 MHz, CD_3CN, 60 ºC): δ 7.99 (d, J = 8.5 Hz, 1H), 7.95 (d, J = 7.5 Hz, 1H), 7.73–7.60 (m, 2H), 7.60–7.32 (m, 14H), 7.09 (br s, 1H), 6.92 (br s, 1H), 6.65 (d, J = 2.0 Hz, 1H), 6.57 (s, 1H), 6.19 (s, 1H), 5.90 (d, J = 4.5 Hz, 1H), 5.45–5.25 (m, 2H), 5.37 (d, J = 12.5 Hz, 1H), 5.27 (d, J = 12.5 Hz, 1H), 5.20–5.05 (m, 2H), 5.02 (br s, 1H), 4.98–4.85 (m, 2H), 4.60 (dd, J = 16.0, 4.0 Hz, 1H), 3.92 (dd, J = 10.0, 7.5 Hz, 1H), 3.71 (d, J = 6.5 Hz, 1H), 3.56 (dd, J = 12.5, 6.0 Hz, 1H), 3.47 (dd, J = 12.0, 7.5 Hz, 1H), 3.18 (t, J = 10.5 Hz, 1H), 3.15–3.02 (m, 1H), 2.75 (d, J = 13.5 Hz, 1H), 2.28–2.18 (m, 1H); IR (KBr): 3380, 2920, 2360, 2102, 1727, 1633, 1523, 1323, 1397, 1352, 1289, 1192, 1136 cm^{-1};HRMS (ESI, *m/z*): $[M+H]^+$ calcd for $C_{47}H_{43}O_{10}N_{13}Cl$, 984.2939; found, 984.2948. Since nitrobenzyl group of **32** was readily removed during the NMR experiment at 60 °C, the spectra of time-consuming ^{13}C NMR was difficult to obtain. Thus, we added a chart of HRMS.

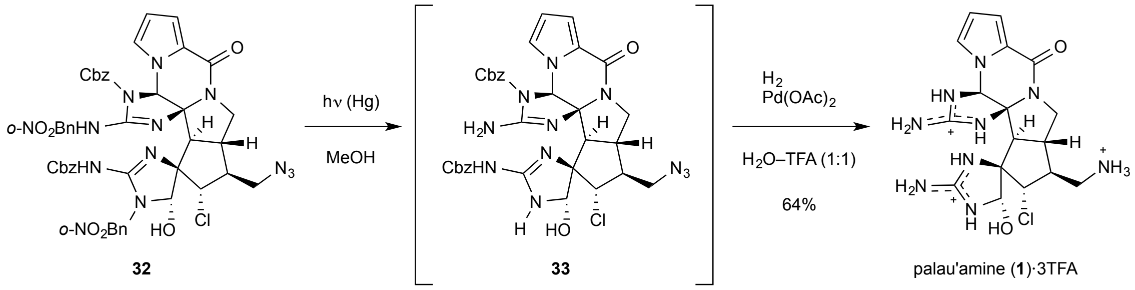

Palau’amine **1**;

A solution of azide **32** (2.5 mg, 2.5 μmol) in MeOH (1.0 mL) was irradiated by Hg-lamp (400 W) at room temperature. After being stirred for 1.5 h, to the reaction mixture were added H_2O (0.5 mL), TFA (0.5 mL) and $Pd(OAc)_2$ (0.9 mg, 3.8 μmol). Then hydrogen gas was bubbled through the mixture for 10 min. After being stirred under hydrogen atmosphere (balloon) at room temperature for 1.5 h, the reaction mixture was filtered through a Cosmonice Filter S (pore size: 0.45 μm, filter diameter 13 mm). The filtrate was concentrated under reduced pressure. The residue was purified by semi-preparative HPLC (Atlantis dC18, 5 μm, 250 x 4.6 mm, 100% H_2O (0.1% HCO_2H), 1 mL/min, R_T = 5.0 min) to give pure palau’amine (**1**) as a formate salt. Azeotropic treatment with TFA provided pure palau’amine (**1**)·3TFA (1.2 mg, 1.6 μmol, 64%) as a off-white solid. ^{1}H NMR (500

MHz, D_2O): δ 7.01 (dd, *J* = 2.5, 1.5 Hz, 1H), 6.87 (dd, *J* = 4.0, 1.5 Hz, 1H), 6.37 (dd, *J* = 4.0, 2.5 Hz, 1H), 6.36 (s, 1H), 5.96 (s, 1H), 4.33 (d, *J* = 7.5 Hz, 1H), 3.95 (dd, *J* = 10.0, 7.0 Hz, 1H), 3.31 (dd, *J* = 13.2, 6.5 Hz, 1H), 3.29 (t, *J* = 10.2 Hz, 1H), 3.26 (dd, *J* = 13.2, 6.5 Hz, 1H), 3.09 (d, *J* = 14.0 Hz, 1H), 2.49 (m, 1H), 2.47 (m, 1H); ^{13}C NMR (125 MHz, D_2O): δ 159.57, 157.94, 157.83, 125.21, 122.50, 115.70, 113.89, 83.76, 80.77, 74.03, 72.06, 69.02, 56.35, 48.58, 46.03, 41.87, 41.87; HRMS (ESI–TOF, *m/z*): $[M+H]^+$ calcd for $C_{17}H_{23}O_2N_9Cl$, 420.1663; found, 420.1663.

ORTEPS drawing of 21 from X-ray crystallographic analysis

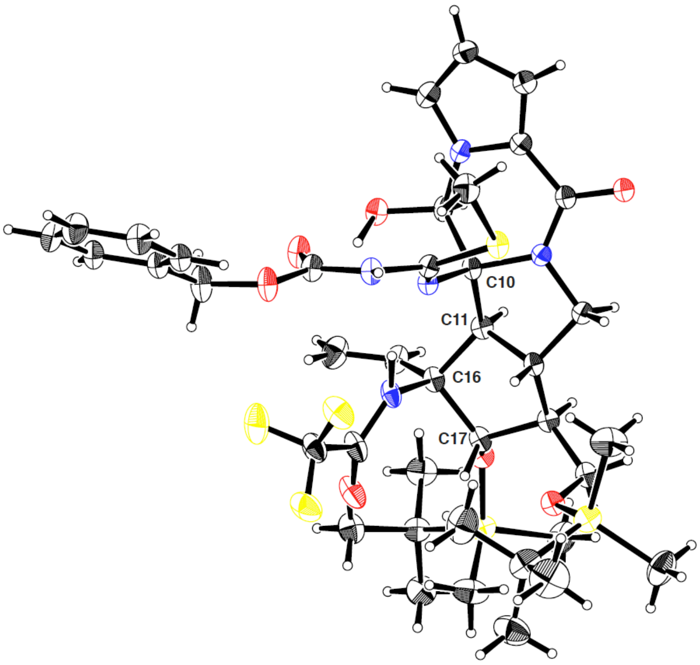

Supplementary Dataset 1

Compound **21** CCDC 1417980

X-ray Structure Report for Compound 21

Experimental

<u>Data Collection</u>

A colorless block crystal of $C_{44}H_{70}F_3N_5O_9SSi_2$ having approximate dimensions of 0.600 x 0.400 x 0.300 mm was mounted on a glass fiber. All measurements were made on a Rigaku R-AXIS RAPID diffractometer using graphite monochromated Mo-Kα radiation.

The crystal-to-detector distance was 127.40 mm.

Cell constants and an orientation matrix for data collection corresponded to a primitive monoclinic cell with dimensions:

a = 13.0627(8) Å

b = 22.718(2) Å   b = 107.908(2)^{o}

c = 18.8564(9) Å

V = 5324.6(5) Å^{3}

For Z = 4 and F.W. = 958.29, the calculated density is 1.195 g/cm^{3}. The reflection conditions of:

h0l: h+l = 2n

0k0: k = 2n

uniquely determine the space group to be:

$P2_1/n$ (#14)

The data were collected at a temperature of -100 $\pm$ 1°C to a maximum 2θ value of 54.9^{o}. A total of 75 oscillation images were collected. A sweep of data was done using w scans from 130.0 to 190.0^{o} in 3.0^{o} step, at χ=45.0^{o} and ϕ = 70.0^{o}. The exposure rate was 120.0 [sec./^{o}]. A second sweep was performed using ω scans from 0.0 to 165.0^{o} in 3.0^{o} step, at χ=45.0^{o} and ϕ = 250.0^{o}. The exposure rate was 120.0 [sec./^{o}]. The crystal-to-detector distance was 127.40 mm. Readout was performed in the 0.100 mm pixel mode.

Data Reduction

Of the 44677 reflections that were collected, 11824 were unique (R_{int} = 0.1118); equivalent reflections were merged.

The linear absorption coefficient, μ, for Mo-Kα radiation is 1.686 cm^{-1}. An empirical absorption correction was applied which resulted in transmission factors ranging from 0.457 to 0.951. The data were corrected for Lorentz and polarization effects.

Structure Solution and Refinement

The structure was solved by direct methods[7] and expanded using Fourier techniques. The non-hydrogen atoms were refined anisotropically. Hydrogen atoms were refined using the riding model. The final cycle of full-matrix least-squares refinement[8] on F^{2} was based on 11799 observed reflections and 647 variable parameters and converged (largest parameter shift was 0.00 times its esd) with unweighted and weighted agreement factors of:

$$R1 = \Sigma ||Fo| - |Fc|| / \Sigma |Fo| = 0.0943 \quad (1)$$

$$wR2 = [\Sigma (w (Fo^2 - Fc^2)^2)/ \Sigma w(Fo^2)^2]^{1/2} = 0.2443 \quad (2)$$

The standard deviation of an observation of unit weight[9] was 1.09. A Sheldrick weighting scheme was used. Plots of $\Sigma w (|Fo| - |Fc|)^2$ versus |Fo|, reflection order in data collection, sin è/ë and various classes of indices showed no unusual trends. The maximum and minimum peaks on the final difference Fourier map corresponded to 1.18 and -0.68 e/Å^3, respectively.

Neutral atom scattering factors were taken from Cromer and Waber[10]. Anomalous dispersion effects were included in Fcalc[11]; the values for Δf' and Δf" were those of Creagh and McAuley[12]. The values for the mass attenuation coefficients are those of Creagh and Hubbell[13]. All calculations were performed using the CrystalStructure[14-15] crystallographic software package.

Supplementary References

1. Namba, K. *et al.* Toward palau'amine: $Hg(OTf)_2$-catalyzed synthesis of the cyclopentane core. *Chem. Eur. J.* **15**, 6560-6563 (2009).
2. Kinnel, R. B., Gehrken, H.-P. & Scheuer, P. J. Palau'amine: a cytotoxic and immunosuppressive hexacyclic bisguanidine antibiotic from the sponge Stylotella agminata. *J. Am. Chem. Soc.* **115**, 3376-3377 (1993).
3. Kinnel, R. B., Gehrken, H.-P., Swali, R., Skoropowski, G. & Scheuer, P. J. Palau'amine and its congeners: a family of bioactive bisguanidines from the marine sponge Stylotella aurantium. *J. Org. Chem.* **63**, 3281-3286 (1998).
4. Buchanan, M. S. Carroll, A. R. & Quinn, R. J. Revised structure of palau'amine. *Tetrahedron Lett.* **48**, 4573-4574 (2007).
5. Seiple, I. B., Su, S., Young, I. S., Lewis, C. A., Yamaguchi, J. & Baran, P. S. Total synthesis of palau'amine. *Angew. Chem. Int. Ed.* **49**, 1095-1098 (2010).
6. Seiple, I. B. *et al.* Enantioselective total synthesis of (-)-palau'amine, (-)-axinellamines, and (-)-massadines. *J. Am. Chem. Soc.* **133**, 14710-14726 (2011).
7. Altomare, A. *et al.* a new tool for crystal structure determination and refinement. *J. Appl. Cryst.* **32**, 115-119 (1999).
8. Least Squares function minimized: $\Sigma w(F_o^2-F_c^2)^2$ where w = Least Squares weights.
9. Standard deviation of an observation of unit weight: $[\Sigma w(F_o^2-F_c^2)^2/(N_o-N_v)]^{1/2}$, where: N_o = number of observations, N_v = number of variables

10. Cromer, D. T. & Waber, J. T. International tables for x-ray crystallography. *The Kynoch Press, Birmingham, England*, **4**, Table 2.2 A (1974).

11. Ibers, J. A. & Hamilton, W. C. *Acta Crystallogr*. **17**, 781 (1964).

12. Creagh, D. C. & McAuley, W. *J. International tables for crystallography*. Vol C, (A.J.C. Wilson, ed.), Kluwer Academic Publishers, Boston, Table 4.2.6.8, 219-222 (1992).

13. Creagh, D. C. & Hubbell, J. H. *International tables for crystallography*. Vol C, (A.J.C. Wilson, ed.), Kluwer Academic Publishers, Boston, Table 4.2.4.3, 200-206 (1992).

14. CrystalStructure 4.0: Crystal Structure Analysis Package, Rigaku Corporation (2000-2010). Tokyo 196-8666, Japan.

15. CRYSTALS Issue 11: Carruthers, J. R. *et al*. Chemical Crystallography Laboratory, Oxford, UK. (1999)
